# Cryo-EM Structure of the TRPC1/5 Heteromer Enables Design of Antidepressant and Anxiolytic Drug with Reduced Side Effects

**DOI:** 10.1038/s41467-026-73409-1

**Published:** 2026-05-23

**Authors:** Yixiang Chen, Tong Che, Xinyu Cheng, Xiaoqiang Yang, Xiaojing Song, Juncheng Li, Ying Fu, Wei Zhang, Sijia Lv, Tingting Yang, Qi Peng, Weiwei Nan, Shuangyan Wan, Yaoguang Hua, Xiaoyun Wu, Han Hu, Yuting Zhang, Yinzhen Liu, Mingxing Yang, Shuqi Zeng, Ougen Liu, Bo Yu, Jingjing Duan, Jian Li, Bing Xiong, Jin Zhang

**Affiliations:** 1 The MOE Basic Research and Innovation Center for the Targeted Therapeutics of Solid Tumors, School of Basic Medical Sciences; The Second Affiliated Hospital, Jiangxi Medical College, Nanchang University, Nanchang, Jiangxi China; 2 Jiangxi Province Key Laboratory of Pharmacology of Traditional Chinese Medicine, School of Pharmacy, First Affiliated Hospital, Gannan Medical University, Ganzhou, Jiangxi 341000 China; 3 Shenzhen Crystalo Biopharmaceutical Co., Ltd, Shenzhen, Guangdong, 518118 China; 4 Key Laboratory of Infection and Immunity, Health Commission of Jiangxi Province & School of Basic Medicine, Nanchang Medical College, Nanchang, 330052 P. R. China; 5 Department of Dermatology, The Second Affiliated Hospital of Nanchang University, Nanchang, Jiangxi China; 6 Sphingolipid Metabolism and Aging, Human Aging Research Institute (HARI) and School of Life Science, Nanchang University, Jiangxi Key Laboratory of Aging and Disease, Nanchang, Jiangxi 330031 China; 7 State Key Laboratory of Chemical Biology, Shanghai Institute of Materia Medica, Chinese Academy of Sciences, Shanghai, 201203 P. R. China

**Keywords:** Transient receptor potential channels, Cryoelectron microscopy

## Abstract

The TRPC1/5 heteromer exhibits electrophysiological and ligand-binding properties distinct from TRPC5 homomers, enabling tissue-specific cellular regulation. Here we present the cryo-EM structure of the TRPC1/5 heterotetramer at 2.8 Å resolution, revealing an asymmetric assembly of three TRPC5 subunits around one TRPC1 subunit. TRPC1 contributes a unique pore-loop configuration and specialized inter-subunit interfaces that sculpt an asymmetrical ion conduction pathway, altering gating and ion selectivity. The heteromer harbors a ligand-binding pocket at the TRPC1-TRPC5 interface absent in homomeric channels. Using this insight, we design JD03-02, a high-affinity antagonist preferentially targeting this pocket with >10,000-fold selectivity for TRPC1/5 over TRPC5 homomers. In mouse models, JD03-02 produces potent anxiolytic and antidepressant effects with reduced off-target activity. These findings elucidate the structural basis of TRPC1/5 function and can guide precision drug design targeting heteromeric ion channels in neuropsychiatric disorders.

## Abstract (web summary)

Using cryo-EM, researchers solve the structure of the TRPC1/5 ion channel complex, revealing a unique drug-binding pocket. This enables design of JD03-02, a selective inhibitor with potential to treat anxiety and depression.

## Introduction

Ion channels are integral to various physiological functions and can form either homomers or heteromers, each exhibiting distinct biophysical properties and functional roles. While ligand-gated channels, like NMDA receptors, are known to form heteromers^1–3^, TRP channels, can function as both homomers and heteromers^4–8^. Among these, the transient receptor potential canonical (TRPC) channels, a subgroup of the TRP family^9^, are essential cellular sensors involved in various physiological processes, including neuronal excitability and synaptic transmission^10–14^. TRPC1, the first mammalian TRP channel to be cloned, is ubiquitously expressed and can form heteromeric channels with members of both the same and different TRP subfamilies, such as TRPC4 and TRPC5^8,15–19^. Yet, despite substantial progress in elucidating TRP homomer structures and functions, the architecture and pharmacology of TRP heteromers remain poorly characterized, limiting our ability to develop subunit-selective modulators.

Within the canonical TRP (TRPC) subfamily, TRPC1, TRPC4, and TRPC5 are highly expressed in key brain regions—particularly the hippocampus, amygdala, and hypothalamus—where they predominantly form heteromeric assemblies^20–25^. In rodents, TRPC1/4 and TRPC1/5 heteromers constitute ~80% of TRPC channels in neurons, with defined stoichiometries (1:3 for TRPC1/4 or TRPC1/5, and 1:2:1 for TRPC1/4/5) that confer unique activation mechanisms and ion-conductance profiles^26^. Gene-knockout studies in mice have revealed contrasting phenotypes for homomeric versus heteromeric channels: TRPC5^−/−^ and TRPC4^−/^^−^ animals exhibit reduced depressive- and anxiety-like behaviors^27,28^, whereas deletion of TRPC1 or TRPC4 individually yields more modest effects but, in combination, profoundly alters seizure susceptibility and cognition^11,24,29,30^.

The functional diversity of TRPC1/4/5 heteromers remains largely unexplored, although recent studies suggest that the presence of these heteromers in different neuronal populations may lead to distinct regulatory roles in various signaling pathways. This hypothesis is supported by findings indicating that the loss of TRPC5 in oxytocin neurons of the hypothalamic paraventricular nucleus results in sex-specific behavioral changes. Male TRPC5 carriers exhibit increased appetite, obesity, and anxiety, whereas female TRPC5 carriers display severe postpartum depression. These phenotypic changes have been validated in mouse models. Notably, overexpression of TRPC5 in oxytocin neurons of these mice reverses the behavioral alterations, further highlighting the unique roles that TRPC5 homomers and heteromers may play in different brain regions^31^. While previous research on TRPC5 knockout mice suggested that inhibition of TRPC5 could yield antidepressant or anxiolytic effects^28,32^, the present study proposes that activation of TRPC5 in oxytocin neurons can reverse depressive-like behaviors. This discrepancy raises the possibility that TRPC1/4/5 heteromers or homomers may have region- and cell-specific roles, and their modulation could offer more precise control over neuronal signaling, especially in the context of psychiatric disorders such as anxiety and depression.

The idea that TRPC1/4/5 heteromers might provide a more refined mechanism for regulating neuronal activity opens up new avenues for drug development. Existing TRPC1/4/5-targeting small molecules, such as HC070, Pico145, have shown promise in treating anxiety and depression^32–34^. However, these compounds lack the necessary selectivity between homomers and heteromers, may resulting in undesirable side effects. Thus, there is a clear need for more selective targeting of TRPC1/4/5 heteromers to improve therapeutic outcomes while minimizing side effects.

Since 2018, high-resolution cryo-EM structures of TRPC4 and TRPC5 homotetramers have been resolved, delineating critical architectural features—conserved lipid-binding pockets and the signature LFW motif—that have guided rational inhibitor design^35–41^. More recently, Won et al. reported the human TRPC1/C4 heterotetramer at near-atomic resolution, revealing a 1:3 TRPC1:TRPC4 stoichiometry and showing that TRPC1 incorporation reshapes monovalent cation selectivity and Ca²⁺ permeability via specific selectivity-filter and central-cavity residues^42^. Despite these advances, the quaternary assembly, gating mechanisms, and ligand ability of TRPC1/5 heteromers remain uncharacterized, and no structure-guided heteromer-selective modulators have yet been developed.

Here, we show the 2.8 Å cryo-EM structure of the TRPC1/5 heterotetramer, revealing an inter-subunit binding pocket absent in TRPC5 homomers. Guided by this structural insight, we design and synthesize JD03-02, a small-molecule inhibitor exhibiting >10,000-fold selectivity for TRPC1/5 heteromers over TRPC5 homomers. In preclinical anxiety and depression models, JD03-02 delivers robust anxiolytic and antidepressant efficacy without the obesogenic or behavioral side effects typical of non-selective TRPC1/4/5 inhibitors. Structural and mutagenesis analyses identify critical residues—Y541/Y542 in TRPC5 and the extracellular pore loop in TRPC1—that govern heteromer assembly and function. These findings elucidate TRPC1/5 heteromer mechanisms and contribute to a framework for targeted drug development. By combining high-resolution structural biology with rational medicinal chemistry, our study not only illuminates the molecular underpinnings of TRPC1/5 heteromer function but also supports the development of a frameowork for precision targeting of ion-channel heteromers. These findings will aid design of next-generation neuropsychiatric therapies with enhanced efficacy and safety profiles.

## Results

### Biochemistry and electrophysiology of TRPC1/C5 heterotetramer

**Fig. 1:**
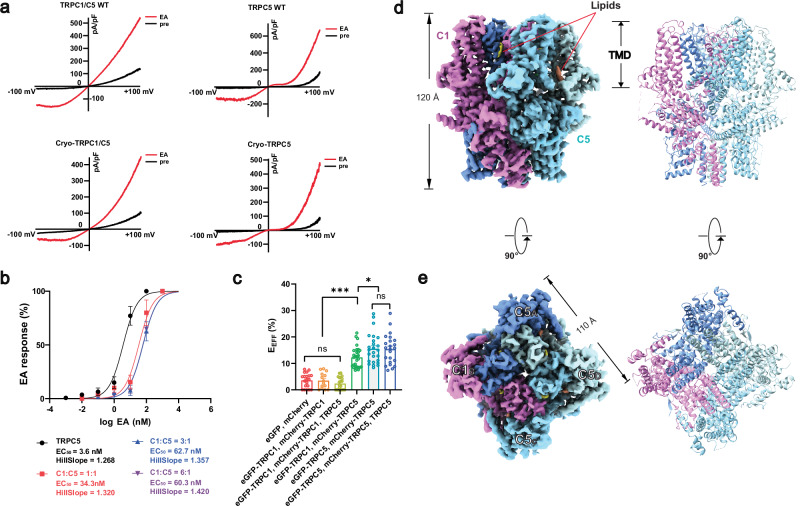
Stoichiometric composition and overall structure of TRPC1/5 heteromer. **a** Representative whole-cells currents in HEK293T cells transfected with either full length hTRPC5 (TRPC5 wild type (WT)) or truncated mTRPC5 (Cryo-TRPC5) constructs alone or co-transfected with recombinant plasmids hTRPC1 (Cryo-TRPC1) and mTRPC5 (Cryo-TRPC5) or wild type plasmids full-length hTRPC1 (WT) and full-length hTRPC5 (WT), described as Cryo-TRPC1/C5 and TRPC1/C5 WT respectively. Currents were recorded before (pre) (black) and after application of 100 nM EA (red). **b** Dose response of EA with EC_50_ resulting from Hill fits (lines) listed. Data are represented as mean ± SEM from *n* = 5 independent experiments (each using a different cell patch). **c** FRET efficiency (*E*_EFF_) summary of the of each expression pair. *n* represents the number of independent cells analyzed from at least three independent transfections: eGFP+mCherry, *n* = 20 cells; eGFP-TRPC1+mCherry-TRPC1, *n* = 14 cells; eGFP-TRPC1+mCherry-TRPC1 + TRPC5, *n* = 21 cells; eGFP-TRPC1+mCherry-TRPC5, *n* = 29 cells; eGFP-TRPC5+mCherry-TRPC5, *n* = 25 cells; TRPC5+eGFP-TRPC5+mCherry-TRPC5, *n* = 23 cells. Data are represented as mean ± SEM. Statistical significance was determined by one-way ANOVA with Waller-Duncan post-hoc test for multiple comparisons. *p < 0.05, ***p < 0.001, ns, not significant. Exact *p* values, *F* statistic (*F* (5,126) = 46.895), and degrees of freedom are provided in Source Data. **d, e** Overall structure of TRPC1/5 heterotetramer. Side and top views of the cryo-EM density map of TRPC1/5 at 2.84 Å overall resolution. TRPC1 subunit represented in pink and TRPC5 subunit represented in light blue, sky blue, and light cyan.

Heteromeric ion channel complexes are challenging to isolate due to low expression levels. We co-expressed Strep-tagged TRPC1 and Flag-tagged TRPC5 in a heterologous system, and successfully purified the TRPC1/5 heteromer using tandem affinity purification. Finally, the structure of the complex was determined at an overall resolution of 2.8 Å using single-particle cryo-electron microscopy (cryo-EM) (Fig. 1d, e and Supplementary Fig. 1a, b).

Transient transfection of these constructs into HEK293T cells enabled robust expression and purification of the TRPC1/5 complex. Importantly, whole-cell patch-clamp recordings confirmed that the functional properties of both heteromeric and homomeric channels were preserved (Fig. 1a), exhibiting current characteristics consistent with wild-type (WT) TRPC1/5 and TRPC5 channels.TRPC5 homomers exhibited large inward currents, whereas TRPC1/5 heteromers displayed NMDA receptor-like current-voltage profiles with slow inward and rapid outward rectification^8^.

### Functional characterization and stoichiometry of the TRPC1/5 heteromer

Previous work by Fakler and colleagues suggested that TRPC1/5 heteromers in the rat brain adopt a stoichiometry of 1:3 (TRPC1:TRPC5)^26^. Recently, cryo-EM structures of (-)-Englerin A (EA) bound to TRPC5 were resolved by Porac et al. Chen et al. and Kim et al. revealing the ligand-binding pocket and the mechanism of action of EA^43–45^. To assess whether this stoichiometry is maintained in vitro, we examined the dose-response relationship of the TRPC1/5 channel to EA—an agonist of TRPC1/C4/C5 channels—under varying TRPC1:TRPC5 transfection ratios.

At a 1:1 (w/w) C1:C5 transfection ratio, the EC_50_ of the TRPC1/5 complex was 34.3 nM, significantly higher than that of TRPC5 homomers (3.6 nM), indicating the presence of both TRPC1/5 heteromers and TRPC5 homomers. When the C1:C5 ratio was increased to 3:1, the EC₅₀ shifted further to 62.7 nM, suggesting a diminished contribution from TRPC5 homomers and a greater predominance of TRPC1/5 heteromeric assemblies. Further increasing the C1:C5 ratio to 6:1 did not produce a significant additional shift in EC_50_ (60.3 nM), indicating that TRPC1 incorporation into the channel complex had reached saturation (Fig. 1b). Our dose-response experiments demonstrate that co-expression of TRPC1 induces a concentration-dependent rightward shift in the EC_50_ of EA for TRPC5-mediated currents. The EC_50_ value reached a plateau when the cDNA transfection ratio of TRPC1 to TRPC5 was increased to 6:1. This observation indicates that TRPC1 and TRPC5 assemble with a fixed, non-random stoichiometry in our HEK293 cell expression system.

To directly examine the subunit composition of the TRPC1/5 heteromer, we further performed Förster resonance energy transfer (FRET) experiments using fluorescently tagged TRPC1 and TRPC5 constructs. Following a strategy previously described for TRPC1/4 heteromers^42^, TRPC1 and TRPC5 subunits were labeled with eGFP or mCherry and co-expressed in HEK293T cells^46^. Significant FRET signals were detected only when both TRPC1 and TRPC5 subunits were tagged, indicating close proximity between them within the complex. This FRET efficiency was comparable to that observed between TRPC5-eGFP and TRPC5-mCherry homomers. In contrast, co-expression of TRPC1-eGFP and TRPC1-mCherry in the presence of untagged TRPC5 did not produce significant FRET signals, indicating limited incorporation of multiple TRPC1 subunits. These findings support a stoichiometry of 1 TRPC1 to 3 TRPC5 subunits in the heteromeric channel complex (Fig. 1c and Supplementary Fig. 2), consistent with both in vivo and in vitro assembly.

### Overall structure of TRPC1/5 heterotetramer

Using a dataset of 4095 cryo-electron microscopy images and extensive data processing, we resolved the structure of the human TRPC1/5 heterotetramer at a global resolution of 2.8 Å (Supplementary Fig. 8 and Table 1). Despite moderate sequence identity between TRPC1 and the TRPC4/5 subfamily ( ~ 46–48%, https://blast.ncbi.nlm.nih.gov/Blast.cgi) distinct side-chain densities in the transmembrane domain enabled reliable assignment of TRPC1 and TRPC5 subunits (Supplementary Fig. 6). The resulting map revealed a heterotetrameric architecture comprising one TRPC1 subunit and three TRPC5 subunits, arranged in a pseudo-symmetric configuration (Fig. 1d, e). Structurally, the TRPC1/5 heterotetramer shares a conserved overall architecture with both the TRPC5 homotetramer and the TRPC1/4 heteromer. In all three complexes, each subunit contains six transmembrane helices and cytosolic domains including the N-terminal ankyrin repeat domain (ARD), helix-loop-helix (HLH) motif, pre-S1 elbow helix, and C-terminal TRP and coiled-coil domains (CCD) (Supplementary Fig. 1c). These shared features contribute to a common tetrameric scaffold across the TRPC subfamily.

Despite these similarities, TRPC1/5 exhibits several distinguishing structural features. Unlike the fully symmetric TRPC5 homotetramer, TRPC1/5 adopts a pseudo-symmetric conformation due to the incorporation of a single TRPC1 subunit, disrupting fourfold symmetry. Compared to TRPC1/4, which also forms a 1:3 heteromer, TRPC1/5 displays greater structural asymmetry-particularly in the ion conduction pathway and the transmembrane helices.

**Fig. 2:**
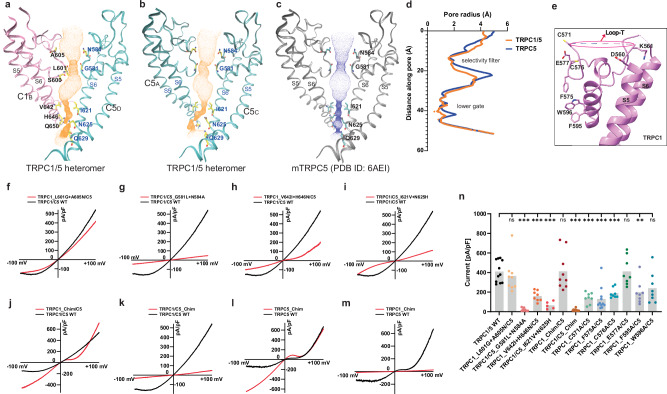
The asymmetric ion-conducting pore of TRPC1/5 heteromer. **a** Side view of TRPC1/5’s pore region with chains B (C1, pink) and C (C5, aquamarine). **b** Side view of TRPC1/5’s pore region with chains A and C (C5, aquamarine). **c** Side view of TRPC5’s pore region with chains A and C (gray). The ion conduction pathway is shown as dots and mapped using HOLE with key amino acid residues labeled. **d** Pore radius along the central axis in the apo state of TRPC1/5 heteromer (in orange) and TRPC5 homomer (in dark blue). **e** The pore-helix and pore-loop of TRPC1, the red arrow points to Loop-T (top). All point mutations of amino acids are displayed in a stick model. **f–i** Patch clamp recordings of TRPC1/5 mutants in response to channel activators EA. Normalized and superposed current traces of TRPC1/C5 WT (black) and each mutant (red). **j, k** Patch clamp recordings of TRPC1/5 chimeras (red) and TRPC1/C5 WT (black) in response to channel activators EA. TRPC1_Chim/C5 (the corresponding TRPC5 segment replaces TRPC1’s Loop-T (indicated by red arrow) and co-transfected with TRPC5); TRPC1/C5_Chim (the corresponding TRPC5 segment was replaced by TRPC1’s Loop-T (indicated by red arrow) and co-transfected with TRPC1). **l, m** Patch clamp recordings of TRPC1 or TRPC5 chimeras (red) and TRPC5 WT (black) in response to channel activators EA. **n** Densities of currents at +100 mV evoked by 100 nM EA for TRPC1/5 WT and mutant constructs. Current density measured as ratio of peak current amplitude to cell membrane capacitance (pA/pF). Data are presented as mean ± SD. Each point represents a single cell patch, with *n* = 8–11 cells per group as indicated in the Source Data file. *p* values were determined by two-tailed Student’s *t*-test in SPSS; *p < 0.05, **p < 0.01, ***p < 0.001 vs. TRPC1/5 WT. “ns” indicates no significant difference. Exact *p* values are provided in Source Data.

Most notably, the selectivity filter and lower gate regions in both TRPC1/5 and TRPC1/4 are narrower than those of the TRPC5 homomer, a consequence of the unique SLAHVA motif in TRPC1, which introduces steric hindrance (Fig. 2a and Supplementary Figs. 3–5). However, TRPC1/5 shows a more pronounced distortion: the S2 helix and the S2–S3 linker in the TRPC5 subunits are displaced leftward, and the CCD of TRPC1 is positioned closer to the adjacent TRPC5, suggesting stronger subunit asymmetry and potential interfacial remodeling. In contrast, the TRPC1/4 heteromer maintains a more symmetric conformation, with only minor displacement observed in the S2 helix of TRPC1 (Supplementary Fig. 4a–f).

In addition, pore loop sequences diverge significantly among TRPC1, TRPC4, and TRPC5, further supporting the notion that heteromer-specific structural features underlie distinct functional properties such as altered ion selectivity, gating dynamics, and ligand responsiveness (Supplementary Figs. 3 and 4g).

### TRPC5 dominantly controls asymmetric gating in TRPC1/5 heteromer

The TRPC1/5 heterotetramer’s ion conduction pathway, comprising the pore helix, pore loop, and S5-S6 helices, displays pronounced asymmetry compared to the symmetric TRPC5 homotetramer, particularly at the upper and lower gates (Fig. 2a–d and Supplementary Fig. 5a–c). The upper gate involves residues L601 and A605 in TRPC1 and G581 and N584 in TRPC5; the lower gate includes H646 in TRPC1 and N625 in TRPC5 (Fig. 2a).

Reciprocal mutations showed that substituting TRPC1 upper gate residues with TRPC5 counterparts had little effect, while the reverse mutations in TRPC5 abolished channel activity (Fig. 2f, g), indicating TRPC5’s dominant role in gating. At the lower gate, TRPC1-to-TRPC5 residue swaps shifted current rectification to a TRPC5-like profile, whereas TRPC5 to TRPC1 mutations reduced current amplitude and altered rectification (Fig. 2h, i, n). These data demonstrate that TRPC5 gating residues primarily control TRPC1/5 channel biophysics.

### A TRPC1-specific pore loop segment governs gating in TRPC1/5 heteromer

Despite overall structural homology, TRPC1/5 heteromers differ electrophysiologically from TRPC5 homomers. A unique segment in the TRPC1 pore loop-termed Loop-T–is absent in TRPC5 (Fig. 2e and Supplementary Fig. 3). Chimeric replacement of Loop-T in TRPC1 with the TRPC5 sequence (TRPC1^Chim^) restored TRPC5-like current properties when co-expressed with TRPC5, whereas TRPC1^Chim^ alone was non-functional. Conversely, introducing Loop-T into TRPC5 (TRPC5^Chim^) preserved TRPC5 homomer function (Fig. 2j–n). This indicates Loop-T in TRPC1 is critical for heteromer-specific gating but dispensable for TRPC5 homomers.

Alanine mutagenesis of TRPC1 pore loop residues C571, F575, and C576 significantly reduced heteromer current amplitude (***p < 0.001) (Fig. 2e, n). Mutations E577A and C576A modulated rectification, shifting gating towards TRPC5-like behavior (Supplementary Fig. 7f, g). Mutations in the conserved LFW motif abolished TRPC5 homomer currents but only altered gating in TRPC1/5 heteromers without reducing current amplitude (Supplementary Fig. 7h, i), further highlighting TRPC5’s dominant gating influence^36^.

### The interface between TRPC1 and TRPC5

**Fig. 3:**
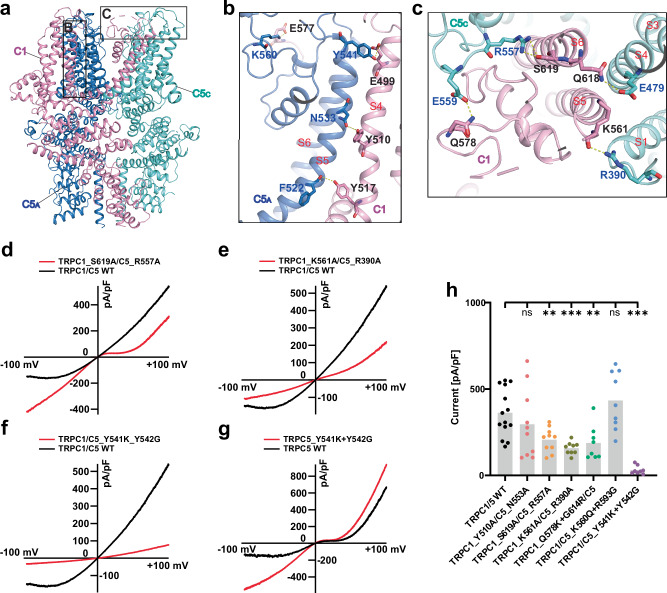
The interaction between TRPC1 and TRPC5 subunits in transmembrane domain. **a** Side view of TRPC1/5 heteromer, the two neighboring TRPC5 subunits and one TRPC1 subunit are colored aquamarine and pink, respectively. **b** The amino acids involved in polar interactions between the S5 and pore-loop regions of the TRPC5 subunit and the S4 and pore-loop regions of the TRPC1 subunit. **c** The amino acids involved in polar interactions between the S1, S4, and pore-loop regions of the TRPC5 subunit and the S5, S6, and pore-loop regions of the TRPC1 subunit. **d–g** Patch clamp recordings of TRPC1/5 mutants in response to channel activators EA. Normalized and superposed current traces of TRPC1/C5 WT (black) and each mutant (red). **h** Densities of currents at +100 mV evoked by 100 nM EA for TRPC1/5 WT and mutant constructs. Current density measured as ratio of peak current amplitude to cell membrane capacitance (pA/pF). Data are presented as mean ± SD. Each point represents a single cell patch, with *n* = 8-14 cells per group as indicated in the Source Data file. *p* values were determined by two-tailed Student’s t-test in SPSS; *p < 0.05, **p < 0.01, ***p < 0.001 vs TRPC1/5 WT, “ns” indicates no significant difference. Exact *p* values are provided in Source Data.

Our analysis of the TRPC1/5 heteromer revealed strong interactions between TRPC1 and adjacent TRPC5 subunits, particularly at the transmembrane interface. Focusing on regions B and C (Fig. 3a), we observed that the S5 and S6 helices of TRPC1 interact with the S4, S5, and S6 helices of the neighboring TRPC5 subunit (Fig. 3b). Similarly, the S4, S5, and S6 helices of TRPC1 also engage with the S5 and S6 helices of an adjacent TRPC5 subunit (Fig. 3c).

Mutating several polar residues within this interaction interface to alanine significantly reduced the current amplitudes of TRPC1^S619A^/C5^R557A^, TRPC1^K561A^/C5^R390A^, and TRPC1^Q578K+G614R^/C5 compared to the TRPC1/C5 WT (**p < 0.01, ***p < 0.001) (Fig. 3h and Supplementary Fig. 7a–c). However, TRPC1^Y510A^/C5^N533A^ and TRPC1/C5^K560Q+R593G^ mutations did not significantly affect the current amplitudes (Fig. 3h). Notably, the current profiles of TRPC1^S619A^/C5^R557A^ and TRPC1^K561A^/C5^R390A^ closely resemble those of C5 homomers (Fig. 3d, e). These results suggest that the mutations disrupt TRPC1-TRPC5 interactions, potentially impairing heteromer assembly and activation. In addition, these polar residues involved in these interactions are not conserved in the TRPC1/C4/C5 complex (Supplementary Fig. 3).

Sequence alignment revealed that TRPC1’s K561 and G562 correspond to TRPC5’s Y541 and Y542, a pattern also observed in TRPC4 (Supplementary Fig. 3a). Mutating TRPC5’s Y541 and Y542 to lysine and glycine, respectively, disrupted the interaction between TRPC5 Y541 and TRPC1 E499. Patch-clamp recordings showed that the heteromeric channel current was undetectable under these conditions. In contrast, the TRPC5^Y541K+Y542G^ double mutant homomer exhibited normal current, similar to TRPC5 WT, suggesting that the disrupted interaction between TRPC1 and TRPC5 impairs TRPC1’s ability to function as part of the heteromeric channel (Fig. 3b, f–h).

### Ca^2+^ binding site in TRPC1/5 heterotetramer

**Fig. 4:**
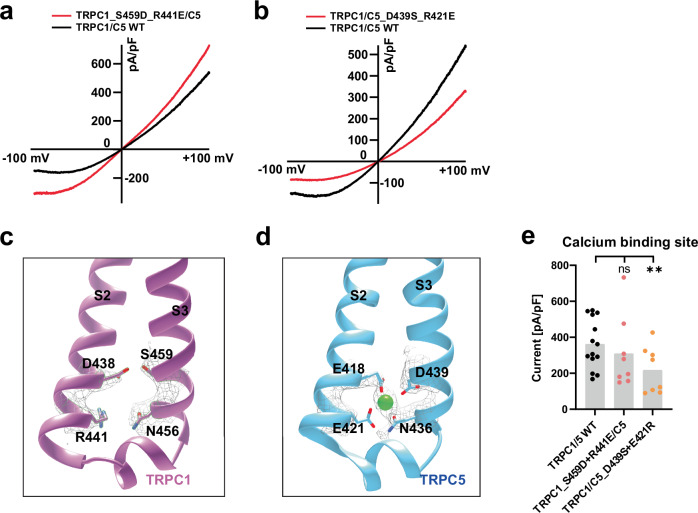
The Ca^2+^ binding site in TRPC1/5 heterotetramer. **a, b** Patch clamp recordings of TRPC1^S459D+R441E^/C5 and TRPC1/C5^D439S+R421E^ heteromer (red) and TRPC1/C5 WT (black) in response to channel activators EA (100 nM). **c** The cartoon representation of S2 and S3 helices of TRPC1. There is no Ca^2+^ binding in this pocket formed by D438, S459, R441, and N456 of TRPC1 subunit. **d** The cartoon representation of calcium binding site formed by E418, D439, E421, and N436 of TRPC5 subunit, showing calcium binding. **e** Densities of currents at +100 mV evoked by 100 nM EA for TRPC1/5 WT and mutant constructs. Current density measured as ratio of peak current amplitude to cell membrane capacitance (pA/pF). Data are presented as mean ± SD. Each point represents a single cell patch, with *n* = 8-14 cells per group as indicated in the Source Data file. *p* values were determined by two-tailed Student’s *t*-test in SPSS; *p < 0.05, **p < 0.01, ***p < 0.001 vs TRPC1/5 WT, “ns” indicates no significant difference. Exact *p* values are provided in Source Data.

The calcium ion binding site in TRPC5 is formed by residues E418, E421, D439, and N436 within the S2 and S3 helices. These negatively charged residues facilitate calcium ion binding^35^. Our structural analysis revealed a similar binding pattern in the TRPC5 subunit, with each TRPC5 subunit coordinating a calcium ion at the respective binding site. However, no calcium ion binding was observed at the analogous position in the TRPC1 subunit (Fig. 4c, d). In TRPC1, residues D438, R441, N456, and S459 occupy the corresponding positions, but they differ from those in TRPC4 and TRPC5 (Fig. 4c, d and Supplementary Fig. 3). Notably, R441 is positively charged, and S459 is neutral, creating an insufficient negative charge to attract calcium ions. Mutagenesis studies of TRPC1^R441E+S459D^ mutation did not alter channel function compared to wild-type TRPC1 (Fig. 4a). In contrast, mutations in TRPC5 (E421R and D439S) that introduced the TRPC1-like residues R441 and S459 significantly reduced the current amplitude (**p < 0.01), although the current shape was preserved (Fig. 4b, e). These results are consistent with prior studies on TRPC5, such as the TRPC5-riluzole study, where mutations in the TRPC5 calcium-binding site impaired TRPC5 activation in response to extracellular calcium^37^.

Our structural and functional data indicate that, unlike TRPC5, the TRPC1 subunit lacks a functional, acidic Ca²⁺-binding pocket, precluding Ca²⁺ coordination at this site. This structural divergence likely alters Ca²⁺ sensing and may contribute to differences in ion selectivity and gating behavior between TRPC1-containing heteromers and TRPC5 homomeric channels.

### Structure-based design of a selective TRPC1/5 heteromeric inhibitor

**Fig. 5:**
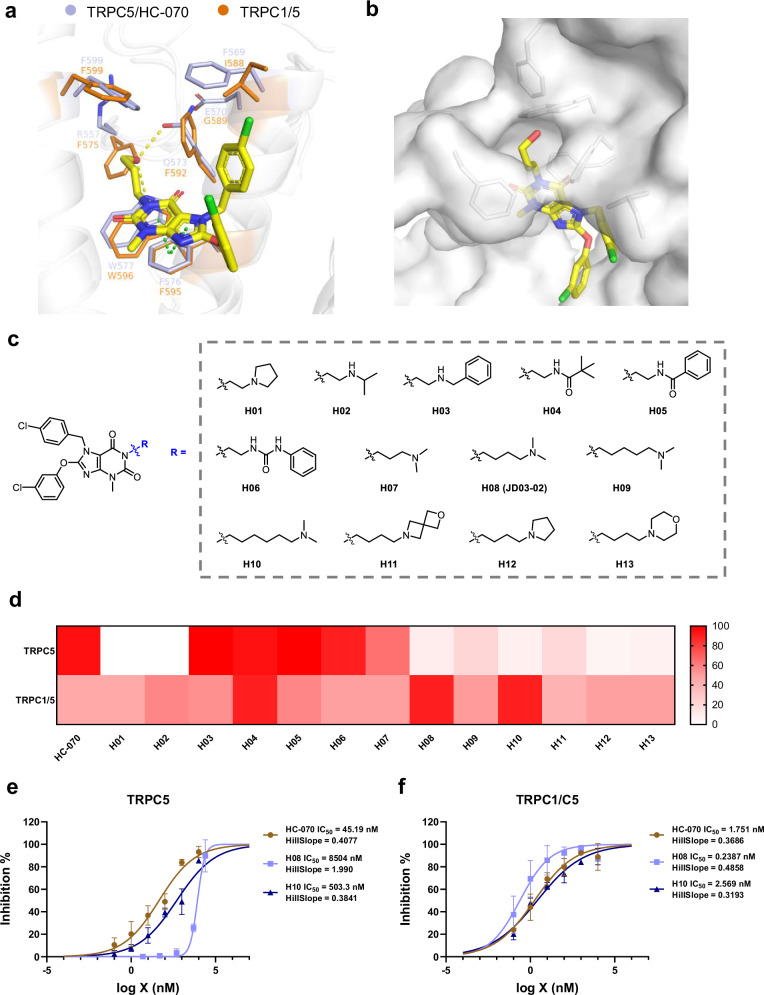
Discovery of selectivity inhibitors for TRPC1/5 heteromeric. **a** The align structure of the HC-070 binding pocket in TRPC5 homomeric and TRPC1/5 heteromeric. H-bonding and π-π stacking interactions are shown as yellow and green dashed lines, respectively. **b** The surface mode of TRPC1/5 in HC-070 binding pocket. **c** Chemical structures of H01-H13. **d**Inhibition of TRPC5 and TRPC1/5 by compounds at 10 μM in indicator-assisted calcium influx analysis. **e–f** Inhibitory effects of HC-070, H08, and H10 on TRPC5 homomers and TRPC1/5 heteromers measured by whole-cell patch-clamp recordings. Data are presented as mean ± SEM (error bars). For TRPC5: HC-070, *n* = 3 independent cells; H08, *n* = 5 independent cells; H10, *n* = 4 independent cells. For TRPC1/5: HC-070, *n* = 3 independent cells; H08, *n* = 5 independent cells; H10, *n* = 3 independent cells.

To develop inhibitors with improved selectivity for TRPC1/5 heteromers over TRPC5 homomers, we examined the structural differences revealed by our high-resolution cryo-EM structure. HC-070, a known nonselective TRPC1/4/5 inhibitor developed by Boehringer Ingelheim^32^, binds to a pocket formed by inter-subunit interactions^35^. In the heteromeric TRPC1/5 channel, this binding pocket is formed by the S5 helix and pore helix of TRPC1 and the S6 helix of TRPC5. Notably, the pocket in the heteromeric context is larger and predominantly hydrophobic, with key contributions from phenylalanine residues (Fig. 5a). Moreover, unlike the TRPC5 homomer, the extracellular-facing portion of the pocket in the heteromer is accessible (Fig. 5b and Supplementary Fig. 9a).

Based on these structural distinctions, we hypothesized that introducing positively charged or aromatic side chains could enhance binding affinity and selectivity toward TRPC1/5. We designed and synthesized a series of derivatives by modifying the hydroxyl tail of HC-070 with amine side chains (cyclopentamine and isopropylamine; compounds H01–H03, (Fig. 5c and Supplementary Fig. 13). Functional screening using calcium flux assays revealed that H01 and H02 markedly reduced inhibitory activity toward TRPC5 homomers while retaining moderate inhibition of TRPC1/5 heteromers (Fig. 5d). Introduction of a bulkier phenyl ring (H03) restored TRPC5 homomer inhibition, indicating sensitivity to substituent size.

Subsequent replacement of the amine with an amide (H04–H06) abolished selectivity, whereas elongation of the amine chain in compounds H07–H10 restored and enhanced selectivity. In particular, compound H08 (4-carbon chain) showed strong inhibition of TRPC1/5 while having minimal effect on TRPC5 homomers. H10 (6-carbon chain) yielded comparable selectivity. Modifications with larger ring structures (e.g., morpholine, pyrrolidine; H11–H13) preserved selectivity but decreased overall activity.

Patch-clamp validation confirmed the calcium flux results: H08 exhibited exceptional selectivity for TRPC1/5 (IC_50_ = 0.2 nM) over TRPC5 (IC_50_ = 8504 nM), representing >10,000-fold selectivity (Fig. 5e, f). Notably, H08 also selectively inhibited TRPC1/4 heteromers over TRPC4 homomers (IC_50_ = 6.7 nM vs. 2385 nM) (Supplementary Fig. 9e), which is likely attributable to the high sequence conservation within the transmembrane regions of TRPC4 and TRPC5 (approximately 85% identity). Consistent with this selectivity profile, H08 (JD03-02) exhibited minimal activity against other closely related TRPC family members, including TRPC3, TRPC6, and TRPC7, producing no more than 40% inhibition at a concentration of 10 μM (Supplementary Fig. 9f–h). Together, these data demonstrate that JD03-02 is a selective antagonist for TRPC1-containing heteromeric channels, with minimal off-target effects on other TRPC subfamily members. Molecular docking revealed that H08’s side chain extended into the heteromeric binding pocket, engaging in cation–π interactions with F592 (TRPC1) and F599 (TRPC5), while its methylxanthine core formed π–π stacking with F595 (TRPC1) (Supplementary Fig. 9b, c). Alanine substitution of F595 in TRPC1 resulted in a ~ 100-fold increase in IC₅₀, confirming its critical role (Supplementary Fig. 9d). These results demonstrate that the unique architecture of the heteromeric binding pocket—characterized by its hydrophobicity, accessibility, and size—can be exploited to design selective inhibitors like JD03-02.

### JD03-02 exhibits rapid and robust antidepressant and anxiolytic efficacy in rodent models

**Fig. 6:**
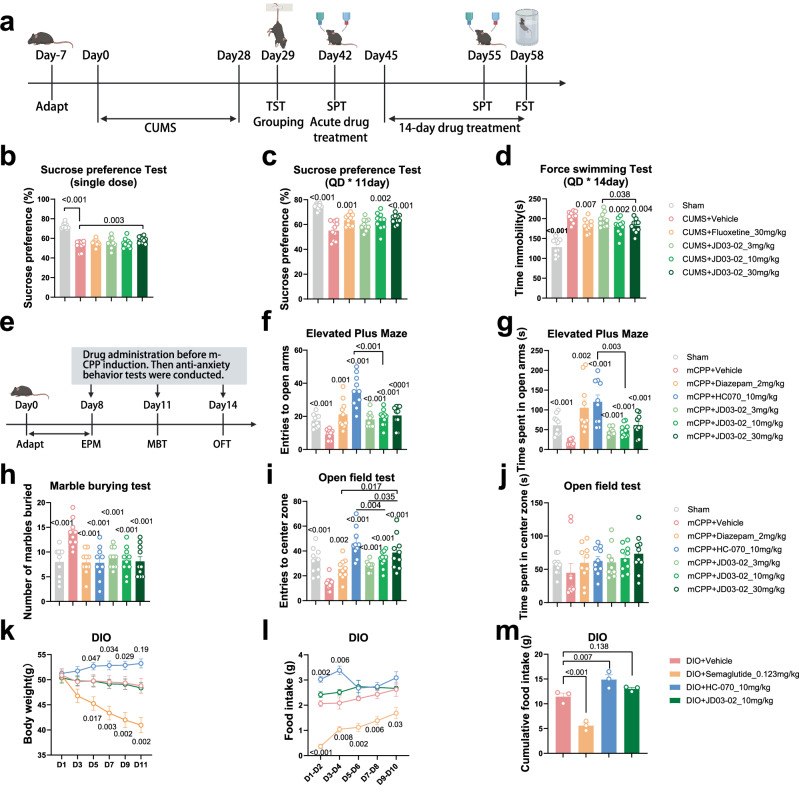
JD03-02 exhibits antidepressant and anxiolytic efficacy with minimal side effects in rodent models. **a** Schematic diagram of the CUMS-induced depression model and behavioral tests. Created in BioRender. Wan, S. (2026) https://BioRender.com/vgzgtp5**b** Sucrose Preference Test: single-dose efficacy of JD03-02 against CUMS-induced depressive-like behaviors. Fluoxetine, vehicle, and sham groups were set as positive control, vehicle control, and sham control, respectively. **c** Sucrose Preference Test: efficacy of JD03-02 after 11 consecutive days of once-daily oral administration against CUMS-induced depressive-like behaviors in mice. Control as in (**b**). **d** Forced Swim Test: efficacy of JD03-02 after 14 consecutive days of once-daily oral administration against CUMS-induced depressive-like behaviors in mice. Control as in (**b**). In b–d, *n* = 10; mean ± SEM; One-way ANOVA with Fisher’s LSD test for multiple comparisons. Each column vs. CUMS + Vehicle or as indicated; exact *p* values shown. **e** Schematic diagram of the mCPP-induced anxiety model and behavioral tests. Created in BioRender. Wan, S. (2026) https://BioRender.com/l09eomk**f–j** Elevated Plus Maze, Marble Burying, and Open Field tests: single-dose efficacy of JD03-02 and HC-070 against mCPP-induced anxiety. Diazepam, vehicle, and sham groups were set as positive control, vehicle control, and sham control, respectively. In **f–j**, *n* = 10; mean ± SEM; Statistical analyses were performed using One-way ANOVA with Fisher’s LSD test (in **f–g**, **i–j**) and Welch’s correction (in **h**) for multiple comparisons. Each column vs. mCPP + Vehicle or as indicated; Exact *p* values shown. **k–m** Weight, daily food intake, and cumulative food intake data were measured to evaluate the efficacy of JD03-02 and HC-070 after 11 consecutive days of once-daily oral administration against diet-induced obesity (DIO). Semaglutide and vehicle groups were set as positive control and vehicle control, respectively. In **k–l**, *n* = 6; **m**, *n* = 3; mean ± SEM; Statistical analyses were performed using two-tailed Student’s *t*-test in **k–i** and One-way ANOVA with Welch’s correction in **m** for multiple comparisons as indicated; *p* values shown.

We evaluated the therapeutic potential of JD03-02 in established mouse models of depression and anxiety. In the Chronic Unpredictable Mild Stress (CUMS) model, after 28 days of stress induction, mice showed significantly increased immobility in the tail suspension test (TST), confirming the depressive-like phenotype (Supplementary Fig. 11A). Surprisingly, a single oral dose of JD03-02 (30 mg/kg) significantly increased sucrose preference in the sucrose preference test (SPT), indicating rapid antidepressant action (Fig. 6a, b), whereas fluoxetine showed no immediate effect, consistent with its delayed onset of action. Following 11–14 days of daily treatment, both JD03-02 and fluoxetine significantly improved sucrose intake in the SPT and reduced immobility in the forced swim test (FST) compared with vehicle group (Fig. 6a, c, d). Additionally, JD03-02 exhibited dose-dependent efficacy, with 10 mg/kg and 30 mg/kg doses producing robust improvements, and the 30 mg/kg dose showed significantly better efficacy than the 3 mg/kg dose in both the SPT and FST. Although statistical differences between JD03-02 and fluoxetine were not significant, JD03-02 at 30 mg/kg showed slightly greater absolute behavioral improvement.

To evaluate anxiolytic effects, the mCPP-induced anxiety model was employed. mCPP administration elicited significant anxiety-like behaviors as measured by the elevated plus maze (EPM), marble burying test (MBT), and open field test (OFT) (Fig. 6e). JD03-02 dose-dependently attenuated these anxiety-related behaviors, increasing numbers of open arm entries and time spent in open arms in the EPM (Fig. 6f, g), reducing the number of marbles buried in the MBT (Fig. 6h), and enhancing numbers of central zone entries and time spent in the central zone in the OFT (Fig. 6i, j). In the EPM and MBT, JD03-02 exhibited efficacy comparable to diazepam, whereas in the OFT, 30 mg/kg JD03-02 showed significantly superior efficacy to diazepam, suggesting JD03-02 may have potential for more optimal therapeutic effects. Compared with the non-selective TRPC5 inhibitor HC-070, 10 mg/kg JD03-02 demonstrated weaker anxiolytic efficacy in the EPM and OFT, while similar efficacy was observed in the MBT. Interestingly, when total locomotor distance traveled in the OFT was quantified, HC-070 induced significantly higher total distance than the vehicle group, as well as JD03-02 and diazepam treatment groups, indicating that HC-070 may cause hyperlocomotion in mice (Supplementary Fig. 11b). This may partially explain why HC-070 showed stronger efficacy in the EPM and OFT, but not in the movement-independent anxiety-like test (MBT). JD03-02 minimizes side effects associated with TRPC5 inhibition

While both JD03-02 and HC-070 exhibit anxiolytic efficacy, clinical data indicate that BI 1358894^47^, a small molecule inhibitor of TRPC4/5 ion channels has been developed and is currently under investigation for the treatment of MDD and post-traumatic stress disorder, is associated with adverse effects such as hyperphagia and weight gain^48–50^. Prior studies suggest that TRPC5 homomers in hypothalamic oxytocin neurons regulate metabolism, raising the possibility that selective inhibition of TRPC1/5 may mitigate these side effects. To test this, we assessed locomotor activity using the OFT. HC-070 increased total distance traveled in both anxious and healthy mice, whereas JD03-02 and diazepam had no significant effect, suggesting that JD03-02 does not alter basal locomotion (Supplementary Fig. 11b, c). Notably, HC-070’s superior performance in EPM and OFT may partly reflect increased locomotor activity rather than anxiolytic specificity.

We next assessed weight gain using a diet-induced obesity (DIO) model. After 11 days of administration, HC-070 significantly increased body weight, beginning on day 5, and also elevated food intake, particularly during the early phase. In contrast, JD03-02 had no significant effect on either parameter (Fig. 6k–m). Semaglutide, a GLP-1R agonist, served as a positive control and effectively reduced both weight and appetite.

These findings underscore that JD03-02, unlike nonselective TRPC5 antagonists, does not induce hyperphagia or locomotor hyperactivity. Thus, selective TRPC1/5 inhibition retains anxiolytic efficacy while avoiding key side effects associated with TRPC5 blockade.

## Discussion

Our study provides the high-resolution structural characterization of the TRPC1/5 heteromeric ion channel, uncovering molecular features that govern its assembly, functional asymmetry, and pharmacological selectivity. While heteromeric assembly is known to confer unique properties on ligand-gated channels such as NMDA receptors, the structural basis and therapeutic relevance of TRPC channel heteromers have remained largely unexplored. Here, using cryo-electron microscopy and structure-guided functional assays, we delineate the architecture of the TRPC1/5 heterotetramer and identify distinct residues and inter-subunit interfaces critical for its formation and gating behavior.

Notably, TRPC1 contributes a non-canonical pore loop and lacks the conserved glutamate-based calcium-binding motif found in TRPC5, resulting in altered ion selectivity and rectification. Structure-guided mutagenesis revealed that TRPC5 residues Y541 and Y542 are essential for maintaining heteromeric current without affecting TRPC5 homomeric function. Mutations at the TRPC1-TRPC5 interface not only disrupted channel assembly but also rewired the channel’s electrophysiological profile to resemble TRPC5 homomers, emphasizing that heteromerization does not merely reflect subunit coexistence but actively defines channel identity. These insights outline a molecular basis for understanding the unique gating and pharmacological features of TRPC1/5 channels.

Beyond structural discovery, we identified a ligand-binding pocket in the TRPC1/5 heteromer. This interfacial site, formed by the S5-S6 helices of TRPC5 and the TRPC1 pore loop, is absent in homomeric TRPC5, enabling rational design of heteromer-selective compounds (Supplementary Fig. 12). Using this pocket, we developed JD03-02, a selective small-molecule inhibitor that exhibits nanomolar potency and over 10,000-fold selectivity for TRPC1/5 over TRPC5 homomers, reflecting the power of structure-based design enabled by heteromer-selective structural data. Pharmacological analysis further supports a distinct mechanism of action. Inhibition of the TRPC1/5 heteromer by JD03-02 is characterized by a low Hill coefficient ( ~ 0.49), consistent with negative cooperativity, similar to that observed for the non-selective inhibitor HC-070, whereas its activity toward TRPC5 homomers is negligible. Together, these features are compatible with a model in which JD03-02 preferentially engages a binding pocket formed at the heteromeric interface, linking structural insights to selective pharmacological modulation.

The therapeutic relevance of these findings is underscored by the performance of JD03-02 in behavioral models of depression and anxiety. In contrast to prior TRPC5-targeting compounds such as BI 1358894, which induce side effects including weight gain and hyperlocomotion, JD03-02 shows robust antidepressant and anxiolytic efficacy without these liabilities. These distinctions likely stem from selective inhibition of TRPC1/5 heteromers while sparing TRPC5 homomers, which are implicated in oxytocinergic regulation of appetite and stress. Our findings support a growing hypothesis that TRPC1/5 heteromers, rather than TRPC5 alone, may serve as the more disease-relevant channel complex in limbic and hypothalamic circuits.

Given the limitations of current antidepressant and anxiolytic therapies—including delayed onset of action, incomplete efficacy, and frequent adverse effects—TRPC1/5-selective modulation represents a mechanistically novel and potentially safer therapeutic strategy. To assess the in vivo antidepressant potential of TRPC1/5 inhibition, we employed the CUMS model, a well-established paradigm that recapitulates key behavioral and physiological features of human depression and remains a critical benchmark in preclinical antidepressant development. Within this model, the TRPC1/5-selective inhibitor JD03-02 robustly reversed CUMS-induced depressive-like behaviors across multiple domains. Acute administration of JD03-02 rapidly alleviated anhedonia and behavioral despair, as evidenced by increased sucrose preference and reduced immobility in the forced swim test, whereas a single dose of fluoxetine was ineffective, consistent with the delayed onset of selective serotonin reuptake inhibitors (SSRIs). Upon repeated administration (11–14 days), JD03-02 produced greater and more sustained behavioral improvements than fluoxetine, with no evidence of tolerance.

Importantly, JD03-02 exhibited favorable drug-like properties, including high oral bioavailability (49.8%) and efficient central nervous system exposure, with a brain-to-plasma ratio (B/P) of 0.67 (Supplementary Tables 2 and Table 3). Chronic dosing did not affect body weight or food intake in diet-induced obesity mice, indicating good tolerability and minimal metabolic liability. At the mechanistic level, TRPC1 and TRPC5 are co-expressed in brain regions implicated in mood regulation, including the amygdala and hippocampus (Supplementary Fig. 10e). Consistent with this expression pattern, ex vivo electrophysiological recordings demonstrated that JD03-02 selectively suppresses CCK-4-induced hyperexcitability of lateral amygdala neurons (Supplementary Fig. 10a–d), supporting a circuit-level mechanism whereby TRPC1/5 inhibition normalizes stress-induced neuronal overactivation. JD03-02 exhibits favorable drug-like properties, including high oral bioavailability (49.8%) and efficient brain penetration (B/P = 0.67), indicating improvement over previously reported TRPC inhibitors. The compound shows high selectivity for TRPC1-containing heteromers, with minimal activity against other TRPC family members (IC_50_ > 10 μM), and demonstrates good tolerability upon repeated dosing. Together with its nanomolar potency and robust antidepressant efficacy in the CUMS model, these features support a potentially wide therapeutic window and position JD03-02 as a promising lead for further preclinical development.

Several questions remain unanswered. First, the extent to which TRPC1/5 heteromers differ functionally from TRPC1/4/5 ternary assemblies across brain regions is unknown. Given the co-expression of TRPC1, TRPC4, and TRPC5 in key nodes of mood and stress regulation—such as the amygdala, hippocampus, and hypothalamus^22^—defining the stoichiometry, dynamics, and activity states of these heteromers will be essential. Future high-resolution structures of TRPC1/4/5 in open and ligand-bound states could reveal new determinants of gating and selectivity.

Second, the physiological distribution of heteromeric TRPC channels in specific neuronal subtypes remains poorly characterized. Cell-type–resolved transcriptomic and proteomic approaches, combined with JD03-02 as a pharmacological probe, could illuminate the endogenous roles of these complexes in behaviorally relevant circuits. Third, given the dominance of TRPC5 in forming the functional pore, it will be important to further dissect how TRPC1 modulates ligand sensitivity and gating kinetics—an understanding that could inform both agonist and antagonist development.

Finally, our findings reinforce a broader paradigm in ion channel pharmacology: selective targeting of heteromeric assemblies offers a powerful strategy for achieving functional precision and avoiding off-target effects. As seen with NMDA receptor subtypes and now TRPC channels, heteromerization endows channels with emergent properties not present in homomers, making them ideal substrates for therapeutic intervention.

A current limitation is the absence of TRPC1/5 structures in complex with small-molecule modulators. Future studies aimed at solving ligand-bound TRPC1/5 complexes, together with functional validation, will be essential for elucidating the molecular mechanisms of heteromer-selective modulation and for advancing structure-based drug design targeting TRPC heteromeric channels. In conclusion, our work provides a structural and functional overview of the TRPC1/5 heteromer, reveals a heteromer-selective druggable interface, and introduces a selective compound with strong therapeutic potential. These findings provide additional avenues for treating depression and anxiety by targeting ion channel assemblies with unprecedented precision and efficacy.

## Methods

### Ethics statement

All animal experiments were conducted in accordance with relevant laws and regulations regarding the use and management of experimental animals and the Institutional Animal Care and Use Committee (IACUC) guidelines. The study was approved by the IACUC of ICE Bioscience Inc. (Approval No: XZICE-IACUC-2024081602) and Greentech Bioscience Co., Ltd. (Approval No: IACUC-B2024102-P-01). The experimental designs, animal numbers, and handling procedures were strictly followed as per the approved protocols. Mice were housed in a temperature-controlled room (24 °C ± 1 °C) with a 12-h light/dark cycle and provided with food and water ad libitum.

### Plasmid construction

A C-terminal truncated 210 residues amino acid sequence of mouse TRPC5 was cloned into the pEG-BacMam vector. For structural analysis, we designed triple Flag tag with a maltose binding protein (MBP) on its N terminus. A full-length amino acid sequence of human TRPC1 with N-terminal MBP and C-terminal eGFP followed twin Strep-tag®Ⅱ was also cloned into the pEG-BacMam vector. For electrophysiological experiments, the full-length human TRPC5 or C-terminal truncated 210 residues amino acid sequence of mouse TRPC5 with N-terminal mCherry was cloned into pcDNA4.0 vector. All mutants in this study were generated from the recombinant plasmids mentioned above through the design and use of modified primers. Details of primer sequences are available from the corresponding author upon reasonable request. All newly generated plasmids are available from the corresponding author upon reasonable request.

### Expression and purification of TRPC1/5 heteromer

The expression and purification of TRPC1/5 heteromer were performed as previously described with slight modification^40^. Briefly, plasmids FMC5 and MGC1 were respectively transformed into *E. coil* DH10Bac competent cells to obtain bacmids. P4 baculovirus were produced in the Bac-to-Bac Baculovirus Expression System (Invitrogen). HEK293S GnTI^−^ (from the American Type Culture Collection (ATCC)) cells were infected with 10% (v/v) P4 baculovirus with a ratio of TRPC1 to TRPC5 of 2:1 (v/v) at a density of 2.0–3.0×10^6^ cells/ml. Following an incubation period of 18–24 h, sodium butyrate was administered to enhance protein expression, which was subsequently induced at 30°C for 48 h. Then cells were harvested and frozen at -80°C. The cell pellet was re-suspended in buffer A (50 mM Hepes pH 7.5, 150 mM NaCl, and 1 mM DTT, 1% (v/v) EDTA-free protease inhibitor cocktail (Roche), 10% (v/v) glycerol, 0.5% (w/v) LMNG) (Anatrace), 0.1% (w/v) CHS (Anatrace)) and then solubilized for 3 h in 4°C. Then the cell lysate was centrifuged for 60 min at 100,000 g, and the supernatant was incubated with Strep-Tactin resin (IBA) at 4 °C for 2 h. The resin was washed once with buffer B (50 mM Hepes pH 7.5, 150 mM NaCl, and 1 mM DTT, 10% (v/v) glycerol, 0.1% (w/v) LMNG) (Anatrace), 0.02% (w/v) CHS (Anatrace)), followed by one wash with buffer C (50 mM Hepes pH 7.5, 150 mM NaCl, and 1 mM DTT, 10% (v/v) glycerol, 0.03% (w/v) LMNG) (Anatrace), 0.006% (w/v) CHS (Anatrace), 0.01% (w/v) GDN (Anatrace)). The protein was eluted with buffer C plus 5 mM desulfurized biotin (Sigma-Aldrich) and then incubated with anti-Flag M2 affinity resin (Genescript) at 4 °C for 1 h. The resin was washed twice with buffer C and the protein was final eluted with buffer C plus 300 μg/mL Flag peptide (Genescript). The protein was further purified on a Superose 6 (10/300 GL) size exclusion column in SEC buffer (25 mM Hepes pH 7.5, 150 mM NaCl, and 1 mM DTT, 0.00675% (w/v) LMNG) (Anatrace), 0.00135% (w/v) CHS (Anatrace), 0.00225% (w/v) GDN (Anatrace). The peak fraction was collected and concentrated to 8.5 mg/mL for Cryo-EM analysis.

### Cryo-EM sample preparation and data acquisition

3 μL of the samples were applied to Quantifoil grid R1.2/1.3 Au 300 mesh grids (glow discharged at 15 mA for 40 seconds with a Glow discharge cleaning system). Grids were blotted with qualitative filter paper in a Vitrobot Mark Ⅳ (Thermo Fisher Scientific) at 4°C and 100% humidity for 3.5 s using a blot force of 2 prior to plunging into liquid ethane and stored in liquid nitrogen until checked.

We used the 300 kV Titan Krios Gi3 microscope (Thermo Fisher Scientific FEI, the Kobilka Cryo-EM Center of the Chinese University of Hong Kong, Shenzhen) to check the grids and collect at 105,000 × magnification (pixel size of 0.83 Å/pixel). The movie stacks were automatically acquired with the defocus range from −1.0 to −2.0 μm. Micrographs were collected with a total dose of ~54 e-/Å^2^. SerialEM 3.7 was used for semiautomatic data acquisition. Summary of detailed data collection was shown in Supplementary Table 1.

### Cryo-EM data processing

A total of 4095 movies stacks were imported into cryoSPARC v4.1.1. After motion corrected, electron-dose weighted and CTF estimation, the initial particles were performed by cryoSPARC blob picker. After four rounds of 2D classification, the good particles proceeded to two rounds of Ab-initio reconstruction and heterogeneous refinement. Then we performed an additional round of 2D classification using the best class of particles and selected the template. And the particles were performed by cryoSPARC template picker. After four rounds of 2D classification, the good particles proceeded to two rounds of Ab-initio reconstruction and heterogeneous refinement. We merged the particles of the two best classes to remove duplicates and the final particle sets were re-extracted with original box size and further applied for final nonuniform refinement and local refinement a density map was obtained with overall resolution of 2.84 Å (determined by Gold-standard Fourier shell correlation (GSFSC) using the 0.143 criterion).

### Model building and refinement for cryo-EM structure

The reference models, PDB 6AEI (mTRPC5) and AF-P48995-F1 (predicted structure of hTRPC1)^40,51,52^, were initially fitted into the EM density map as rigid bodies using Chimera^53^. This fit was further optimized using the jiggle fit function in Coot^54^, followed by manual adjustments with the real-space refine zone function in Coot to produce an atomic model. Subsequently, the model was refined with the real_space_refine tool in Phenix^55^. Validation was performed using MolProbity and Mtriage. PyMOL and Chimera were employed for additional structural analysis and figure generation.

### Electrophysiology

The full-length hTRPC5 or truncated mTRPC5, and the full-length hTRPC1 constructs was respectively co-transfected in a plasmid ration 1:3 (w/w) into HEK293T cells (from the ATCC). As previously described, a mCherry was inserted into TRPC5 constructs and a GFP was inserted at the C terminus of TRPC1. Therefore, Cells with red and green fluorescence were selected for whole-cell patch recordings (HEKA EPC10 amplifier, PATCHMASTER software (https://www.heka.com/downloads/downloads_main.html#down_patchmaster)). A 1-s ramp protocol from −100 to +100 mV was applied at a frequency of 0.2 Hz. Signals were sampled at 50 kHz and filtered at 2.9 kHz. The pipette solution contained 130 mM CsCl, 1 mM MgCl_2_, 5.7 mM CaCl2, 10 mM EGTA, and 10 mM Hepes (calculated free Ca^2+^, 200 nM), and the pH was titrated to 7.2 using CsOH^40^. The standard bath solution contained 140 mM NaCl, 5 mM KCl, 1 mM MgCl_2_, 2 mM CaCl_2_, 10 mM Hepes, and 10 mM D-glucose, and the pH was adjusted to 7.4 with NaOH (1 M). The recording chamber (150 μL) was perfused at a rate of ~2 mL/min. All recordings were performed at room temperature. In this experiment, TRPC5 homomers or TRPC1/5 heteromers were activated using the TRPC1/4/5 agonist Englerin A (EA).

### Electrophysiological recordings in brain slices

Coronal brain slices (300 μm) were prepared from male *C57BL/6 J* mice (8–10 weeks, Jinan Pengyue Laboratory Animal Breeding Co., Ltd., Jinan, China) and initially incubated in ACSF-1.5 at 32 for 30 min, followed by an additional 1 h at room temperature. Subsequently, slices were transferred to oxygenated artificial cerebrospinal fluid (ACSF) containing the test compound and incubated for 2–3 h before being placed in a recording chamber perfused with ACSF-1.5.

Electrophysiological recordings were performed under an Olympus microscope equipped with a 40× long-working-distance objective. Recording electrodes (3-4 MΩ) were pulled from borosilicate glass capillaries. Data were acquired using an Axonpatch 700B amplifier and pClamp 10.5 software. After achieving a gigaseal in voltage-clamp mode at −70 mV and establishing whole-cell configuration with series resistance compensation, recordings were switched to current-clamp mode. Step currents ranging from −60 pA to 300 pA (Δ = 20 pA, 500 ms duration, 3 s interval) were applied to evoke action potentials. Solutions: ACSF (mM): 185 Sucrose, 2.5 KCl, 1.25 NaH₂PO₄·2H₂O, 25 NaHCO₃, 25 D-Glucose, 0.5 CaCl₂·2H₂O, 10 MgSO₄; saturated with 95% O₂/5% CO₂, pH 7.2-7.4. ACSF-1.5 (mM): 125 NaCl, 2.5 KCl, 1.25 NaH₂PO₄·2H₂O, 25 NaHCO₃, 10 D-Glucose, 2 CaCl₂·2H₂O, 1.5 MgSO₄; saturated with 95% O₂/5% CO₂, pH 7.2-7.4. Intracellular solution (mM): 140 K-gluconate, 2 MgCl₂, 8 KCl, 10 HEPES, 2 Na₂-ATP, 0.2 Na₂-GTP; pH adjusted to 7.2 with KOH.

### Calcium flux assay

Full-length, non-tagged hTRPC1 and hTRPC5 constructs are used for calcium flux assays. hTRPC5 is transfected alone or co-transfected with hTRPC1 into HEK293T cells (70–80% confluency) using PEI as the transfection reagent. At 18–24 h post-transfection, cells are plated into 96-well poly-L-lysine-coated plates (Costar, black) and cultured overnight in DMEM (Corning) supplemented with 10% FBS (ExCell Bio) at 37 °C in a 5% CO_2_ incubator.

At 48 h post-transfection, calcium flux assays are conducted using the Calcium 5 Assay Kit (Molecular Devices). The culture medium is removed, and 100 µL of loading buffer (Calcium 5 Assay Kit) is added to each well, followed by a 1-h incubation at 37 °C in 5% CO_2_. Five minutes before measurement, molecules are added to each well at a final concentration of 10 µM under light-sensitive conditions. TRPC1/4/5 agonist Englerin A (100 nM) is used to activate the channels. Fluorescence is measured with a FlexStation 3 (Molecular Devices), using excitation/emission wavelengths of 485/525 nm and an emission filter set at 515 nm.

### Fluorescence resonance energy transfer

In order to perform the FRET experiment with confocal microscopy, specialized 20-mm-aperture confocal dishes with borosilicate-glass bottom were applied for the culture of HEK293T cells. Six different experimental groups of FRET measurements were designed to identify the stoichiometry, namely, eGFP+mCherry, eGFP-TRPC1+mCherry-TRPC1, eGFP-TRPC1+mCherry-TRPC1 + TRPC5, eGFP-TRPC1+mCherry-TRPC5, eGFP-TRPC5+mCherry-TRPC5, eGFP-TRPC5+mCherry-TRPC5 + TRPC5, in each of which all plasmids were co-transfected in the same proportion. FRET experiments were conducted in the sight of the 40× objective lens of the inverted LSM 980 (Axio Observer 7). In short, FRET efficiency (*E*_EFF_) was measured in the method of acceptor photobleaching. Excitation beam at wavelength 488 nm (eGFP) and 561 nm (mCherry) were used for live screening to seek appropriate cells in sight. Cells were kept on the condition of 37°C, 5%CO_2_. On the cells to be measured, we frame a region of interest for 561 nm (mCherry) light bleaching, with another similar one on the empty area for comparison. While necessary parameters were checked, experiment would be started, and stopped after 10 cycles or the fluorescent intensity of EGFP stopped increasing with the decrease of mCherry fluorescent intensity. In each cycle and before the first, intensity of the two types of fluorescence would be recorded and a snap would take place. The data tables of fluorescent intensity were exported for calculating the FRET efficiency (*E*_EFF_) according to the following formula: *E*_EFF_ = (*D*_post_ -*D*_pre_)/*D*_post_ × 100%, of which *D*_post_ means the intensity of donor (eGFP) after bleaching and *D*_pre_ before bleaching. More than ten typical and valid *E*_EFF_ data were picked for statistical analysis, histogram plotting and line chart drawing. All confocal images in this article came from the snaps during FRET experiments.

### RNAScope multiplex fluorescent RNA in situ hybridization

Tissue samples from male *C57BL/6 J* mice (7–8 weeks, Jinan Pengyue Laboratory Animal Breeding Co., Ltd., Jinan, China) were fixed in 10% neutral buffered formalin for 16–32 h at room temperature, followed by dehydration, clearing, and paraffin embedding. Sections (4 µm) were mounted on SuperFrost Plus slides, dried overnight, and baked at 60 °C for 60–90 min. Deparaffinization was performed with xylene and 100% ethanol.

Antigen retrieval was carried out in pre‑boiled 1× RNAscope® Target Retrieval Reagent (98–102 °C) for 15 min, followed by treatment with RNAscope® Hydrogen Peroxide for 10 min at room temperature. A hydrophobic barrier was drawn around each section using an ImmEdge pen. Protease digestion was performed with RNAscope® Protease Plus at 40 °C for 30 min in a HybEZ™ oven.

For multiplex detection, probes targeting Mm‑Trpc1 (#440441: RNAscopeTM Probe-Mm-Trpc1, C1) and Mm‑Trpc5 (#476241: RNAscopeTM Probe-Mm-Trpc5, C2) were hybridized at 40 °C for 2 h. Signal amplification was achieved using the RNAscope® Multiplex Fluorescent V2 Kit (ACD) with sequential application of AMP1 (30 min), AMP2 (30 min), and AMP3 (15 min) at 40 °C. Opal fluorophores (520, 570; ACD) were diluted 1:10 in TSA buffer and applied accordingly for fluorescence detection. All steps were followed by stringent washes with 1× RNAscope® Wash Buffer. Appropriate positive (Polr2a‑C1, Ppib‑C2, Ubc‑C3) and negative (DapB) control probes (RNAscope® 3-plex Negative Control Probe and RNAscope® 3-plex Positive Control Probe-Mm; ACD) were included. Slides were stored with desiccant at room temperature between key steps, and all reagents were prepared fresh or used within recommended stability periods to preserve RNA quality and assay performance. Fluorescent images were scanned using the 3DHistech (Pannoramic MIDI) system and subjected to magnification analysis.

### Molecular docking

The cryo-EM structure of TRPC1/5 was prepared in the protein preparation Wizard Workflow of Glide in Maestro (Schrödinger Release 2021-2). The energy of the protein structure was minimized using the OPLS4 force field until the RMSD of the heavy atoms converged to 0.3 Å. The center of the three-dimensional receptor grid (20 × 20 × 20 Å) was generated by centroid of F595 via Receptor Grid Generation module. The LigPrep module of Maestro was used to generate the three-dimensional (3D) conformations and applied the OPLS4 force field to minimize the energy of the molecules. During the process, ligands were prepared at pH 7.0 ± 2.0 to estimate their protonation using the Epik module and with maximal 32 stereoisomer generations. The extra precision mode of Glide was used to predict the binding poses of H08.

### General information of chemical synthesis

All starting materials, solvents, and reagents were used directly as obtained commercially without further purification unless otherwise noted. ^1^H NMR and ^13^C NMR spectra were recorded using CDCl_3_, DMSO-*d*_6_, or MeOD-*d*_4_ on a Bruker Avance 300 MHz spectrometer. Chemical shifts are reported in parts per million referenced with respect to residual solvent (MeOD-*d*_4_) 3.31 ppm, (DMSO-*d*_6_) 2.50 ppm, and (CDCl_3_) 7.26 ppm or from internal standard tetramethylsilane (TMS) 0.00 ppm. Coupling constants (*J*) are expressed in hertz (Hz). Chemical shifts (δ) of NMR are reported in parts per million (ppm) units. The first-order peak patterns are indicated as s (singlet) and d (doublet). All compounds submitted for testing were confirmed to be > 95 % purity by LC-MS traces.

### CUMS model induction protocol

After acclimatization, male 7–8-week-old *C57BL/6 J* mice (Jinan Pengyue Laboratory Animal Breeding Co., Ltd., Jinan, China) were subjected to a 28-day chronic unpredictable mild stress (CUMS) protocol. The stressors included food and water deprivation, foot shock, wet bedding, cage tilting, restraint, shaking, and olfactory stimulation. These seven stressors were randomly applied each week to ensure unpredictability. The stress induction was maintained continuously for 28 days. Following model establishment, the tail suspension test (TST) was performed to confirm the successful induction of depressive-like behaviors. Mice that met the criteria were randomly assigned to experimental groups.

### Drug administration and behavioral testing

Acute Treatment: A single dose of the test compound was administered, followed by the sucrose preference test (SPT).

Chronic Treatment: Mice were treated daily for 14 consecutive days. The SPT was conducted on day 11, and the forced swimming test (FST) was performed on day 14.

### Behavioral test

#### Tail suspension test (TST)

Mice were gently handled for 10–15 min and allowed to acclimate to the testing environment for 60 min. The distal one-third of the mouse’s tail was fixed with adhesive tape, and the animal was suspended from a bracket with its head 15 cm above the base. The test was recorded for 6 min, and the immobility time during the last 4 min was analyzed. The testing chamber was cleaned with 75% ethanol after each trial.

#### Sucrose preference test (SPT)

Mice were acclimated to 1% sucrose solution and plain water for 2 days. On the second day, the positions of the sucrose solution and water were swapped to eliminate positional bias. After habituation, mice were deprived of food and water for 24 h. Mice were provided with pre-weighed 1% sucrose solution and plain water. After 24 h, the remaining volumes of sucrose solution (A) and water (B) were measured. Sucrose preference (%) was calculated as Sucrose Preference = *A*/(*A* + *B*)×100.

#### Forced swim test (FST)

Mice were gently handled for 10–15 min and allowed to acclimate to the testing environment for 60 min. They were then placed in the swim chamber for 6 min, and the immobility time during the last 4 min was recorded. One day prior to testing, mice underwent a single habituation swim session. The water depth was adjusted to prevent the mice from touching the bottom with their limbs. Mice were placed in the swim chamber for 6 min, and the immobility time during the last 4 min was recorded. The cumulative immobility time during the last 4 min was analyzed.

### Animals and experimental procedure for anxiety model

Seventy male 7–8-week-old *C57BL/6 J* mice from Jinan Pengyue Laboratory Animal Breeding Co., Ltd. (Jinan, China) were randomly and equally divided into seven groups for the experiment. After a 7-day acclimatization period, interventions were carried out for the indicated days. The Elevated Plus Maze (EPM) test was conducted on day 8, the Marble-Burying (MB) test on day 11, and the Open Field (OF) test on day 14. Mice were brought to the experimental site 2 h before the start of the experiment for acclimatization. Before the behavioral experiments, each group received specific interventions as follows: control group (Normal saline, NS, 10 mL/kg, i.g., 0.5 h before the behavioral test), model group (NS, 10 mL/kg, i.g., 0.5 h before the behavioral test), positive group (Diazepam, 2 mg/kg, i.p., 0.5 h before the behavioral test), HC-070 group (10 mg/kg, i.g., 1 h before the behavioral test), JD03-02 low-dose group (3 mg/kg, i.g., 4 h before the behavioral test), JD03-02 medium-dose group (10 mg/kg, i.g., 4 h before the behavioral test), and JD03-02 high-dose group (30 mg/kg, i.g., 4 h before the behavioral test). Anxiety models were induced 30 min before the experiment using the following protocols: control group (NS, 10 mL/kg, i.p.), other groups (mCPP, 2 mg/kg, i.p.)^56^.

The behavioral testing procedures for anxiety animals are as follows:

#### Elevated plus maze (EPM) test

The Elevated Plus Maze (EPM) test utilizes an apparatus with two enclosed arms (30 cm × 5 cm) and two open arms (30 cm × 5 cm × 25 cm) arranged in a cross shape. Each mouse is placed at the center of the maze facing an open arm and observed for 10 min. Prior to the test, animals are acclimated to the experimental environment for 60 min. To minimize the influence of olfactory cues from previous subjects, the maze is cleaned with a 75% ethanol solution after each trial. Parameters indicating anxiolytic-like behavior, such as time spent in the open arms and entries into the open arms, are evaluated by analyzing recorded video footage. Any mouse that accidentally falls out of the maze is excluded from the experiment.

#### Marble-burying test

The marble-burying test (MBT) was conducted in a square box (38 × 32 × 28 cm) with a 5 cm layer of sawdust on the floor and twenty green clear glass marbles (1.5 cm diameter, arranged in a 5 × 4 grid) evenly spaced. Prior to the test, the animals were acclimated to the testing environment for 60 min. The test lasted for 30 min, and after each round, the bedding was replaced and the cage was disinfected. The number of marbles buried was recorded, with burying more than 50% of the marbles considered as the criteria for burying.

#### Open-field test

The Open-Field test (OFT) was utilized to evaluate the autonomous behavior, exploratory tendencies, and anxiety levels of experimental animals in a new environment. Each mouse was placed at the center of the container and given a 10-min exploration period. Before the test, the animals were acclimated to the experimental setting for 60 min. To minimize the potential impact of odors from previous subjects, the apparatus was thoroughly cleaned with a 75% ethanol solution after each trial to eliminate any lingering scents. This cleaning procedure aimed to reduce the risk of contamination and prevent any misleading experimental results due to olfactory stimuli. Anxiety levels and exploratory behavior were assessed by measuring the time spent in the central area, the number of entries into the central area, and the total distance traveled by the mice.

### Animals and experimental procedure for obesity model

Twenty-four male 14-week-old *C57BL/6 J* mice with high-fat diet-induced obesity were obtained from SPF (Beijing) Biotechnology Co., Ltd. (Beijing, China) and acclimatized for 2 weeks in the SPF area of the animal facility at Greentech Bioscience Co., Ltd. During the acclimatization period, the general condition of the animals was monitored, and any unfit animals were excluded from the experiment. A maximum of five mice were housed per cage during the acclimatization period, and three mice per cage during the experimental period. After acclimatization, the mice were randomly divided into the following groups with six mice per group: DIO-Vehicle group (NS, i.g.), DIO-Semaglutide group (0.123 mg/kg, s.c.), DIO-HC-070 group (10 mg/kg, i.g.), DIO-JD03-02 group (10 mg/kg, i.g.). The day of administration was defined as day 1 (D1) of the experiment, with a single dose/day administered for 11 consecutive days. Daily observations were made on the animals’ general condition. Body weight was measured on days 1, 3, 5, 7, 9, and 11 post-administrations. Food intake was measured on days 1, 3, 5, 7, and 9 post-administrations by calculating the difference between the amount of food provided and the amount remaining in the cage, divided by the number of animals in the cage to determine the average food intake per mouse. The cumulative food intake during the experiment was calculated by summing the food intake measurements taken on different days.

### Quantification and statistical analysis

Data analysis was performed using GraphPad prism 10.1.2 and IBM SPSS statistics 27. The electrophysiological data were expressed as mean ± SEM, *n* represents number of independent measurements. Differences between wild type and mutants were evaluated by independent samples T-Test. *p < 0.05; **p < 0.01; ***p < 0.001; and ns, *p* > 0.05.

### Reporting summary

Further information on research design is available in the Nature Portfolio Reporting Summary linked to this article.

## Supplementary information

Supplementary Information

Reporting Summary

Transparent Peer Review file

## Source data

Source Data

## Data Availability

The atomic coordinates and cryo-EM maps generated in this study have been deposited in the Protein Data Bank (PDB) under accession code 9K4I and the Electron Microscopy Data Bank (EMDB) under accession code EMD-62060 The map and model identifiers are detailed in Supplementary Table 1. Additional source data have been deposited in Figshare (10.6084/m9.figshare.30171676). Source data for all figures are also provided with this paper as a Source Data file. Source data are provided with this paper.
