# Supplementary material for: Cryo-EM Structure of the TRPC1/5 Heteromer Enables Design of Antidepressant and Anxiolytic Drug with Reduced Side Effects
Source: Nat Commun. 2026 May 23;17:6770. doi: 10.1038/s41467-026-73409-1 (PMC13385394; doi:10.1038/s41467-026-73409-1)
Supplement: Supplementary file 1 — Supplementary Information [file 41467_2026_73409_MOESM1_ESM.pdf]

Cryo-EM Structure of the TRPC1/5 Heteromer Enables Design of Antidepressant and Anxiolytic Drug with Reduced Side Effects

Yixiang Chen[1†], Tong Che[1,2†], Xinyu Cheng[1†], Xiaoqiang Yang[3†], Xiaojing Song[4], Juncheng Li[1], Ying Fu[1], Wei Zhang[1], Sijia Lv[1], Tingting Yang[1], Qi Peng[1], Weiwei Nan[3], Shuangyan Wan[1], Yaoguang Hua[1], Xiaoyun Wu[3], Han Hu[3], Yuting Zhang[3], Yinzhen Liu[3], Mingxing Yang[3], Shuqi Zeng[3], Ougen Liu[5], Bo Yu[1], Jingjing Duan[6*], Jian Li[2*], Bing Xiong[7*], Jin Zhang[1*]

Supplementary Information

- Supplementary Figures 1-13
- Supplementary Tables 1-3
- General procedures for compound synthesis
- Characterization data (^{1}H NMR, ^{13}C NMR, LC-MS, HPLC purity)

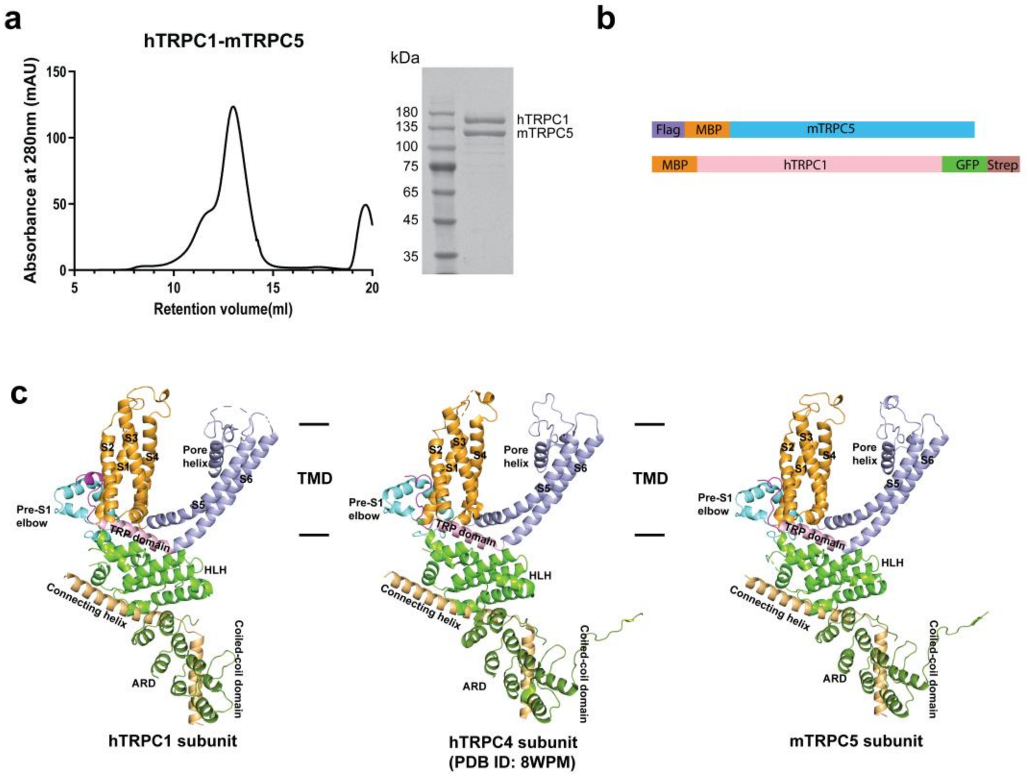

Supplementary Figure 1. Expression and purification of TRPC1/5 heteromer. (a) Size exclusion chromatography of TRPC1/5 heteromer. The peak fractions of TRPC1/5 (between the two red dashed lines) were collected and concentrated for cryo-EM studies. **(b)** Summary of the cryo-EM construct design for TRPC1/5 heteromer. **(c)** Ribbon diagrams depicting structural details of hTRPC1, hTRPC4 (PDB ID: 8WPM) and mTRPC5 subunits. Uncropped scans of the gel are provided at the end of the Supplementary Information file.

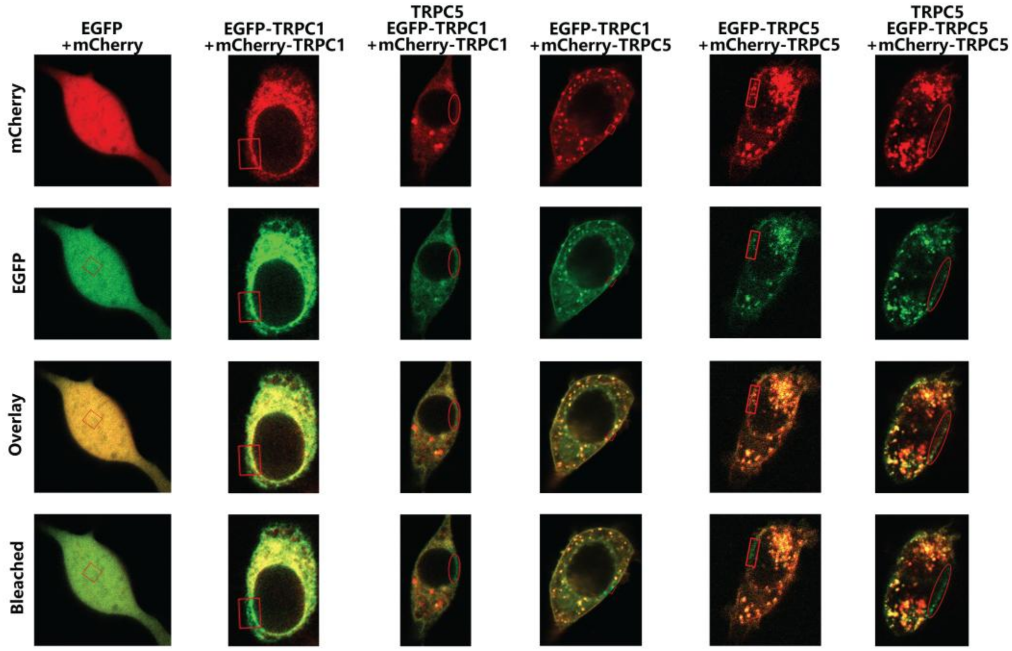

Supplementary Figure 2. Representative confocal fluorescent images and line charts of six FRET experimental groups. Each column representing a different group of expression pair (i.e. 1st column, eGFP+mCherry, n = 20 cells; 2nd column, eGFP-TRPC1+mCherry-TRPC1, n = 14 cells; 3rd column, eGFP-TRPC1+mCherry-TRPC1+TRPC5, n = 21 cells; 4th column, eGFP-TRPC1+mCherry-TRPC5, n = 29 cells; 5th column, eGFP-TRPC5+mCherry-TRPC5, n = 25 cells; 6th column, eGFP-TRPC5+mCherry-TRPC5+TRPC5, n = 23 cells) and each row a different type of result or data (i.e. 1st row, mCherry epifluorescence images; 2nd row, eGFP epifluorescence images; 3rd row, overlayed fluorescent images; 4th row, overlayed fluorescent images after bleaching mCherry;). The rectangle or oval red frame in every fluorescent image represents the selected region of experiment for both photobleaching and a continuous record of fluorescence intensity. This graph related to Figure 1C.

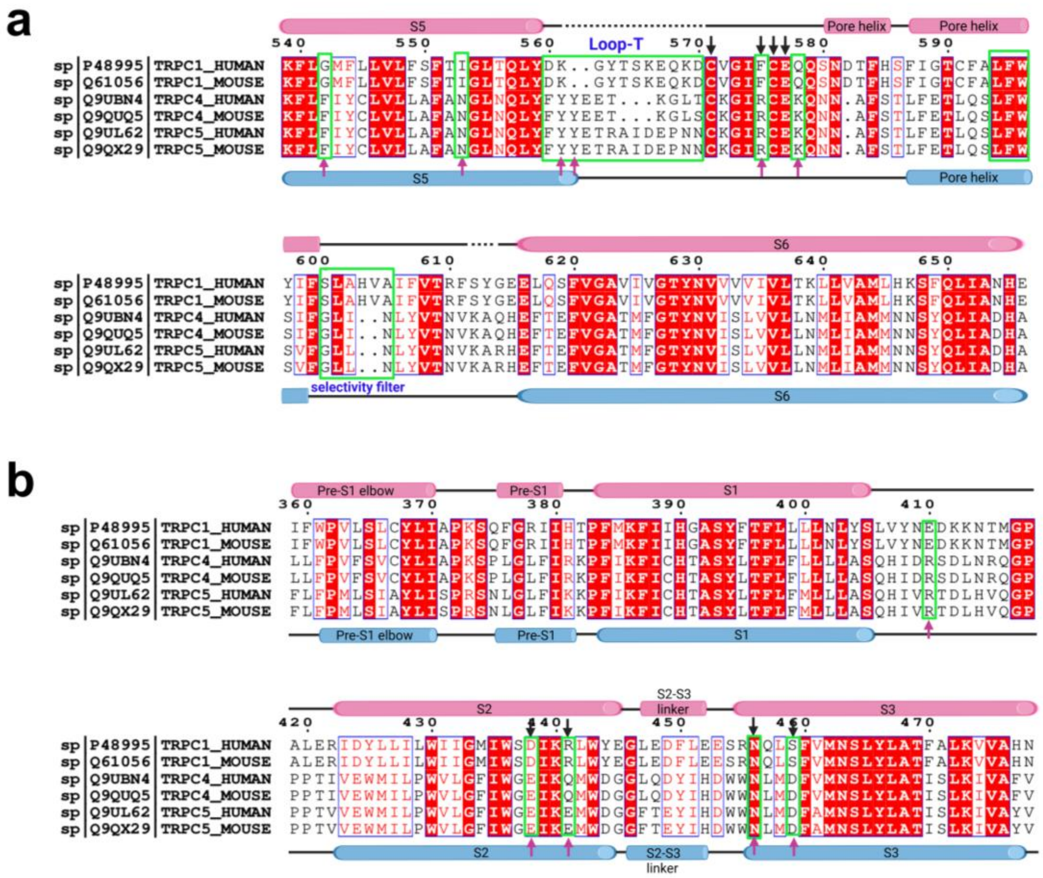

Supplementary Figure 3. Sequence alignment between human and mouse TRPC1/C4/C5. Secondary structure assignments are based on the structure of apo TRPC1/5, TRPC1 is pink and TRPC5 is blue. The black arrows indicate the residues of TRPC1, and the red arrows indicate the residues of TRPC5. **(a, b) Related to Figure 3, Figure 4, and Figure 5.** Created in BioRender. Wan, S. (2026) https://BioRender.com/8t8p9w4

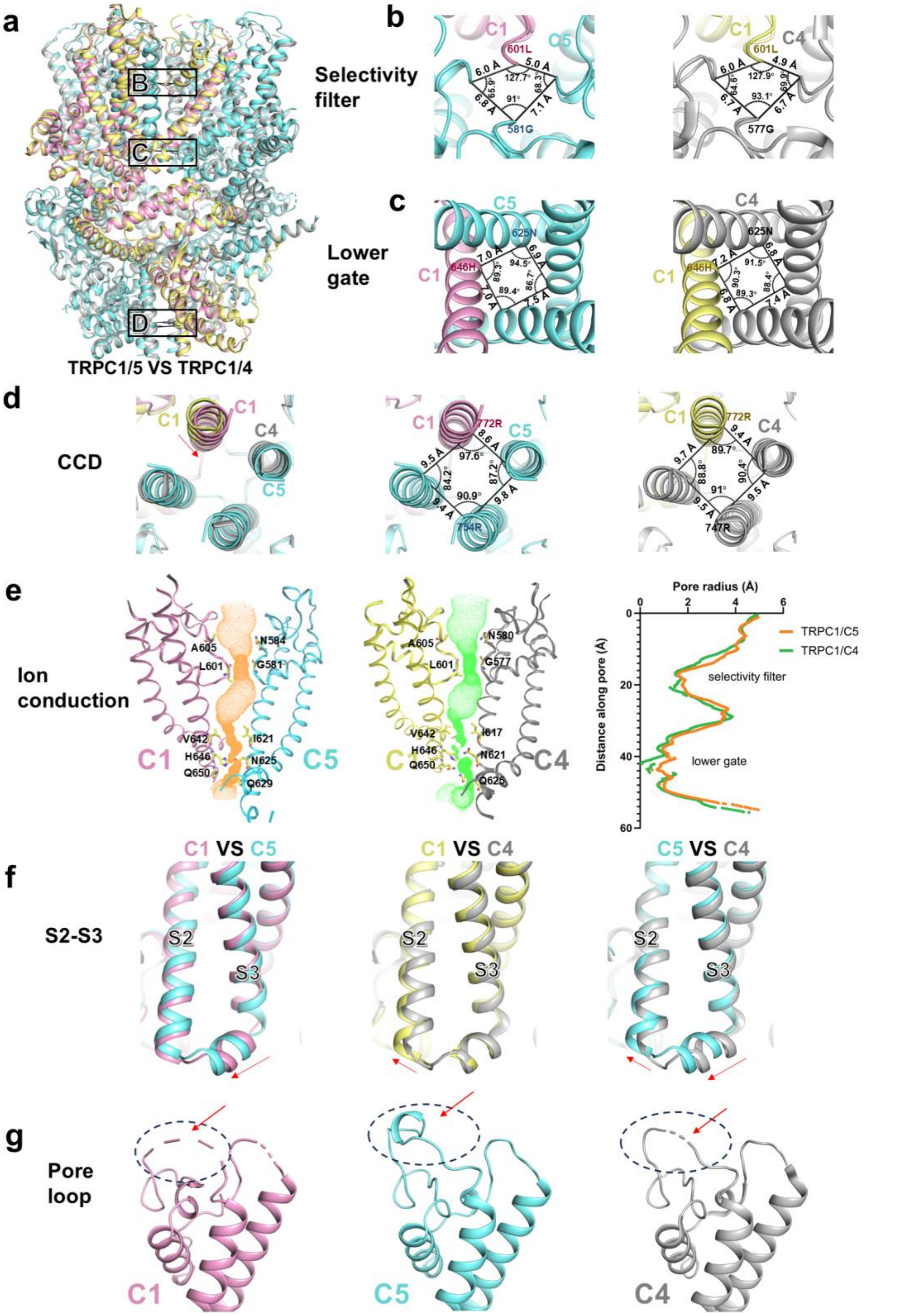

Supplementary Figure 4. Structural comparison of key domains in TRPC1/5 and TRPC1/4 heteromers. (a) Superimposition of the TRPC1/5 and TRPC1/4 heteromers. The TRPC1 subunit is colored pink, and the three TRPC5 subunits are depicted in cyan. In the TRPC1/4 heteromer, the TRPC1 subunit is shown in yellow, while the three TRPC4 subunits are in gray. **(b-d)** The top views of the selectivity filter, lower gate, and coiled-coil domain were analyzed by constructing quadrilaterals connecting the Cα atoms of the

specific residues (as marked) from adjacent subunits. **(e)** Comparison of the ion conduction pathways between TRPC1/5 and TRPC1/4 heteromers. **(f)** Comparison of the S2-S3 helices among TRPC1 and TRPC5 subunits, TRPC1 and TRPC4 subunits, and TRPC5 and TRPC4 subunits. **(g)** Comparison of the pore loops among TRPC1, TRPC5, and TRPC4 subunits, shown in different colors as indicated.

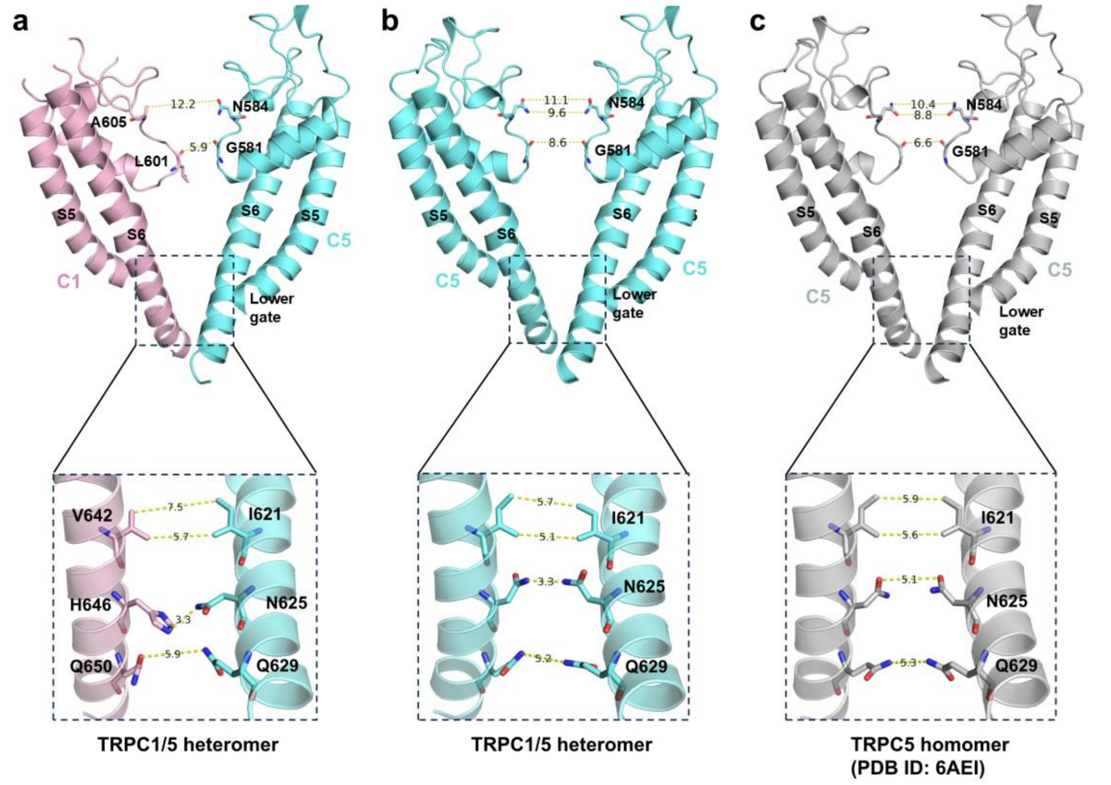

Supplementary Figure 5. Comparison of the upper gate and lower gate between the TRPC1/5 heteromer and TRPC5 homomer. (a) Side view of TRPC1/5's pore region with chains B (TRPC1 colored by pink) and D (TRPC5 colored by aquamarine). The side chains of L601 (TRPC1) and G584 (TRPC5) form a narrow constriction at the selectivity filter. "VHQ" motif of TRPC1 and "INQ" motif of TRPC5 forms the lower gate, and the narrowest point is between H646 and N625. **(b)** The side view of the ion conduction pore between the two TRPC5 subunits (chains B and D) in the TRPC1/5 heteromer. **(c)** Side view of TRPC5's pore region with chains A and C (gray).

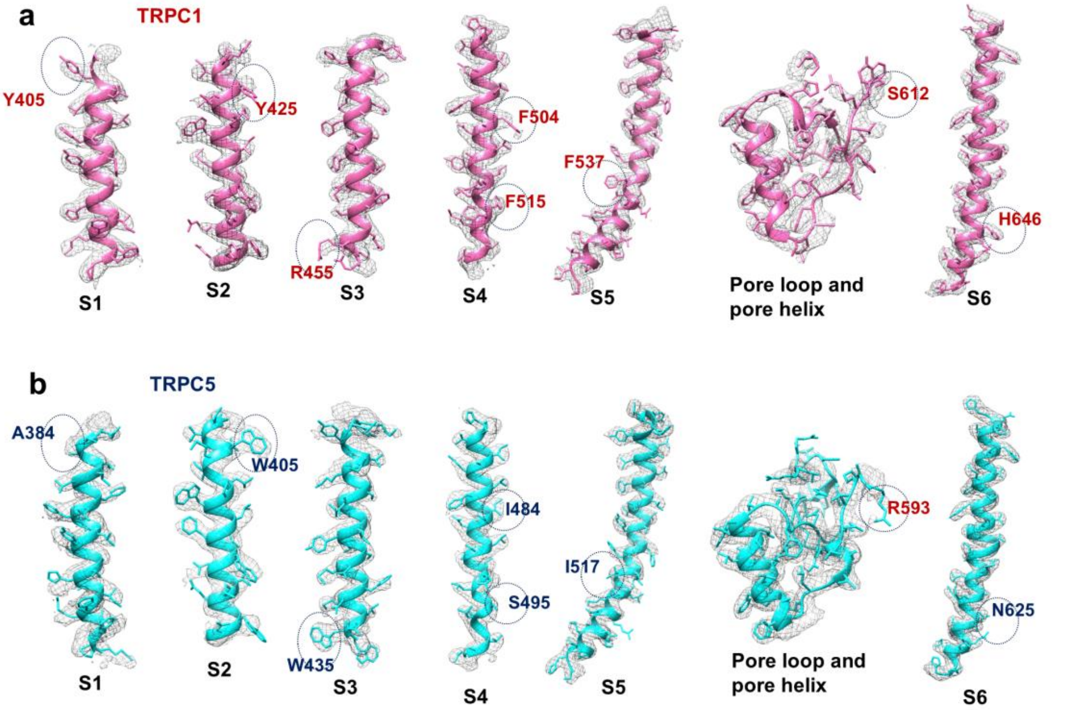

Supplementary Figure 6. Cryo-EM density for TRPC1/5 heteromer. (a) Cryo-EM densities for each segment of the transmembrane helices of TRPC1. **(b)** Cryo-EM densities for each segment of the transmembrane helices of TRPC5. The black dashed circle indicates the different residues or regions between TRPC1 and TRPC5 in cryo-EM density maps.

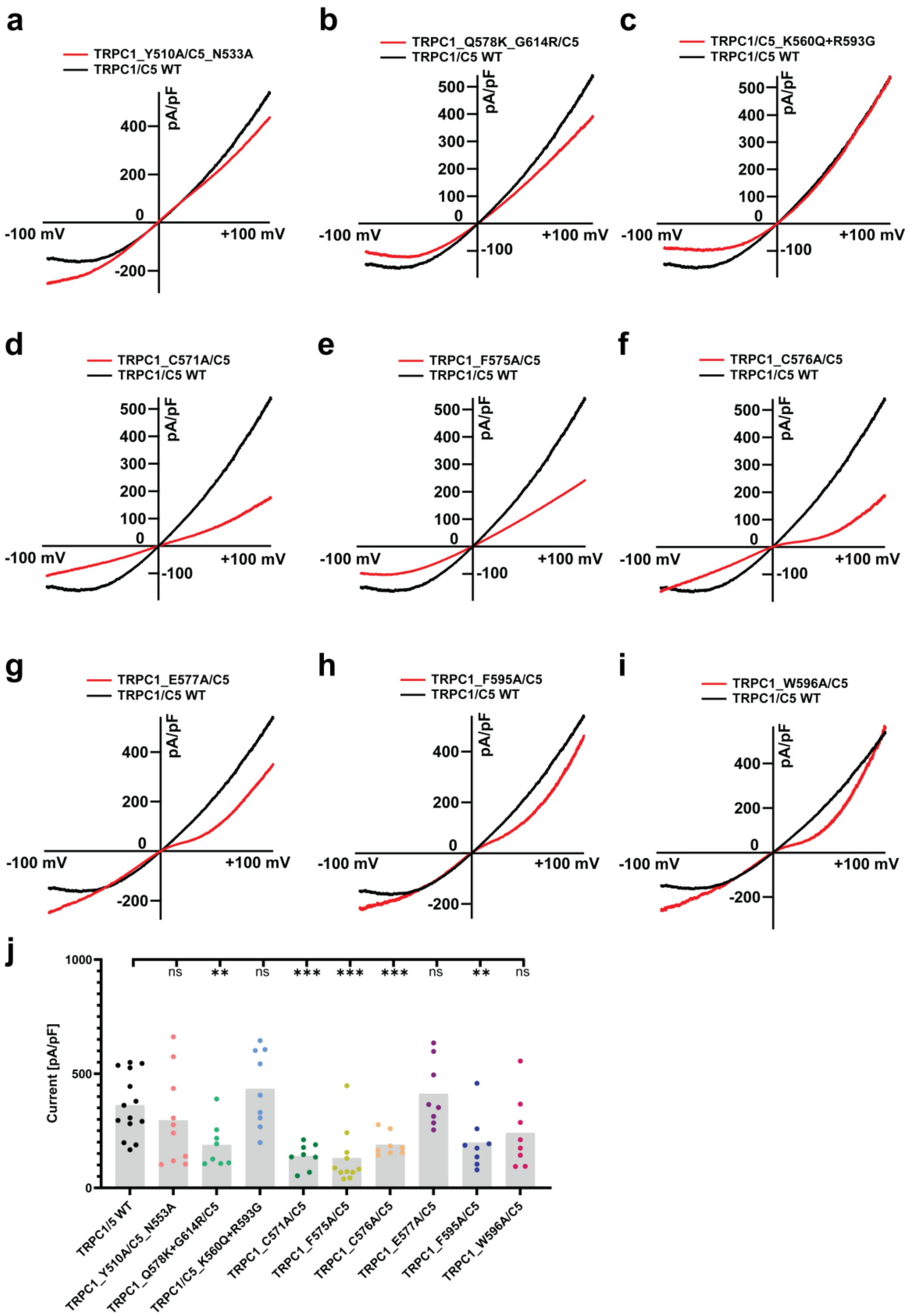

Supplementary Figure 7. The influence of TRPC1's pore-loop, poor-helix, and TRPC1-TRPC5 interface mutations on TRPC1/5 heteromer activation. (a-c) Current profiles of TRPC1/5 heteromer with various mutations in the TRPC1 or TRPC5 subunit (red) compared to TRPC1/5 WT (black). **(d-i)** Current profiles of TRPC1/5 heteromer with mutations specifically in the TRPC1 subunit (red) compared to TRPC1/5 WT (black). **(j)**

Densities of currents at +100 mV evoked by 100 nM EA for TRPC1/5 WT and mutant constructs. Current density measured as ratio of peak current amplitude to cell membrane capacitance (pA/pF). Data are presented as mean ± SD. Each point represents a single cell patch, with n = 8-14 cells per group as indicated in the Source Data file. *p* values were determined by two-tailed Student's t-test in SPSS; **p* < 0.05, ***p* < 0.01, ****p* < 0.001 vs TRPC1/5 WT, “ns” indicates no significant difference. Exact *p* values are provided in Source Data.

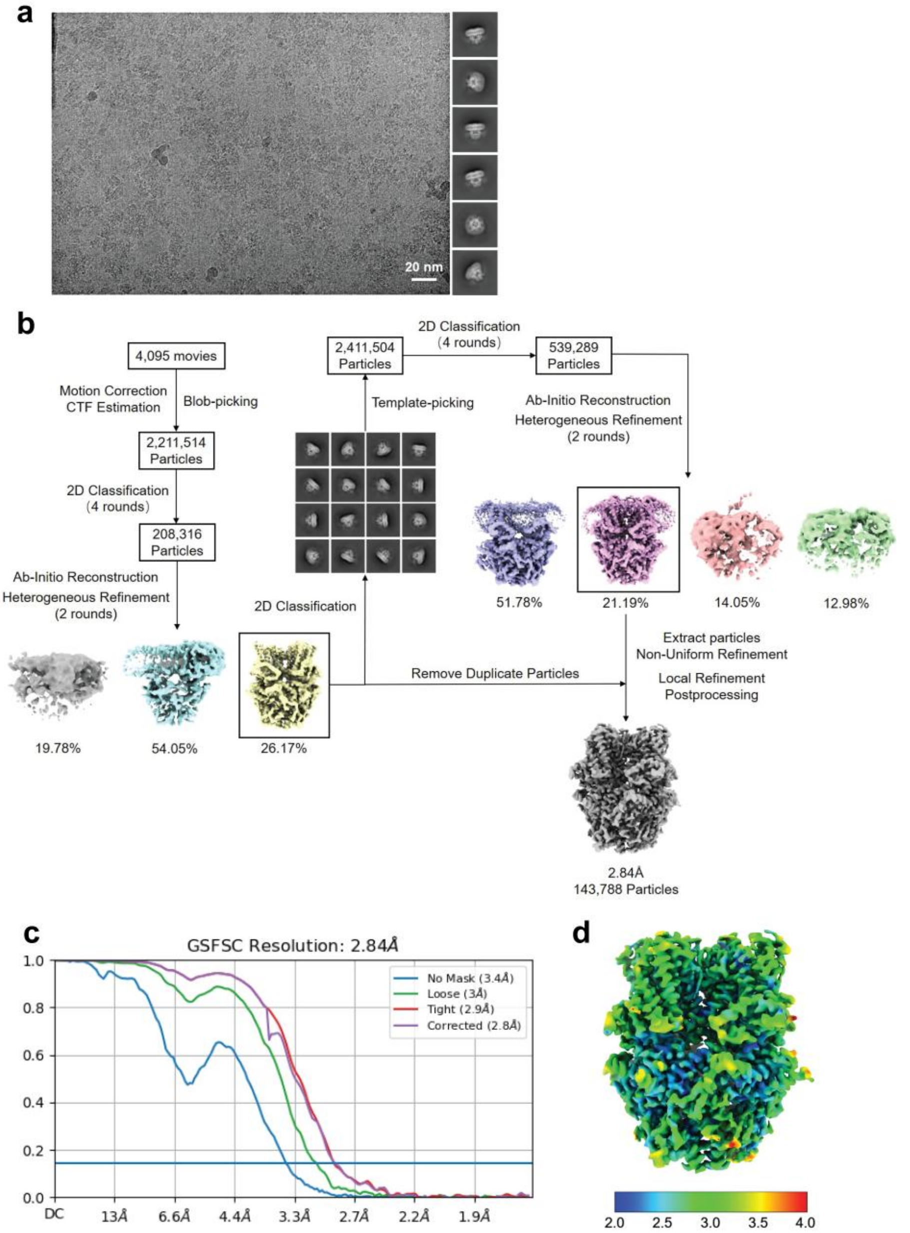

Supplementary Figure 8. Flow chart for TRPC1/5 cryo-EM data processing. (a) Representative of micrograph from 4,095 movies of TRPC1/5 sample in vitreous ice (left) and 2D class averages (right). Scale bar is at 20 nm. **(b)** Flow chart of the cryo-EM data processing procedure. Representative image of 3D reconstructions from final non-uniform refinement and local refinement, and the final density map of TRPC1/5 heteromer with overall resolution of 2.84 Å. **(c)** Gold-standard Fourier shell correlation (GSFSC) curves for the 3D reconstruction of TRPC1/5 heteromer. **(d)** Local resolution estimation.

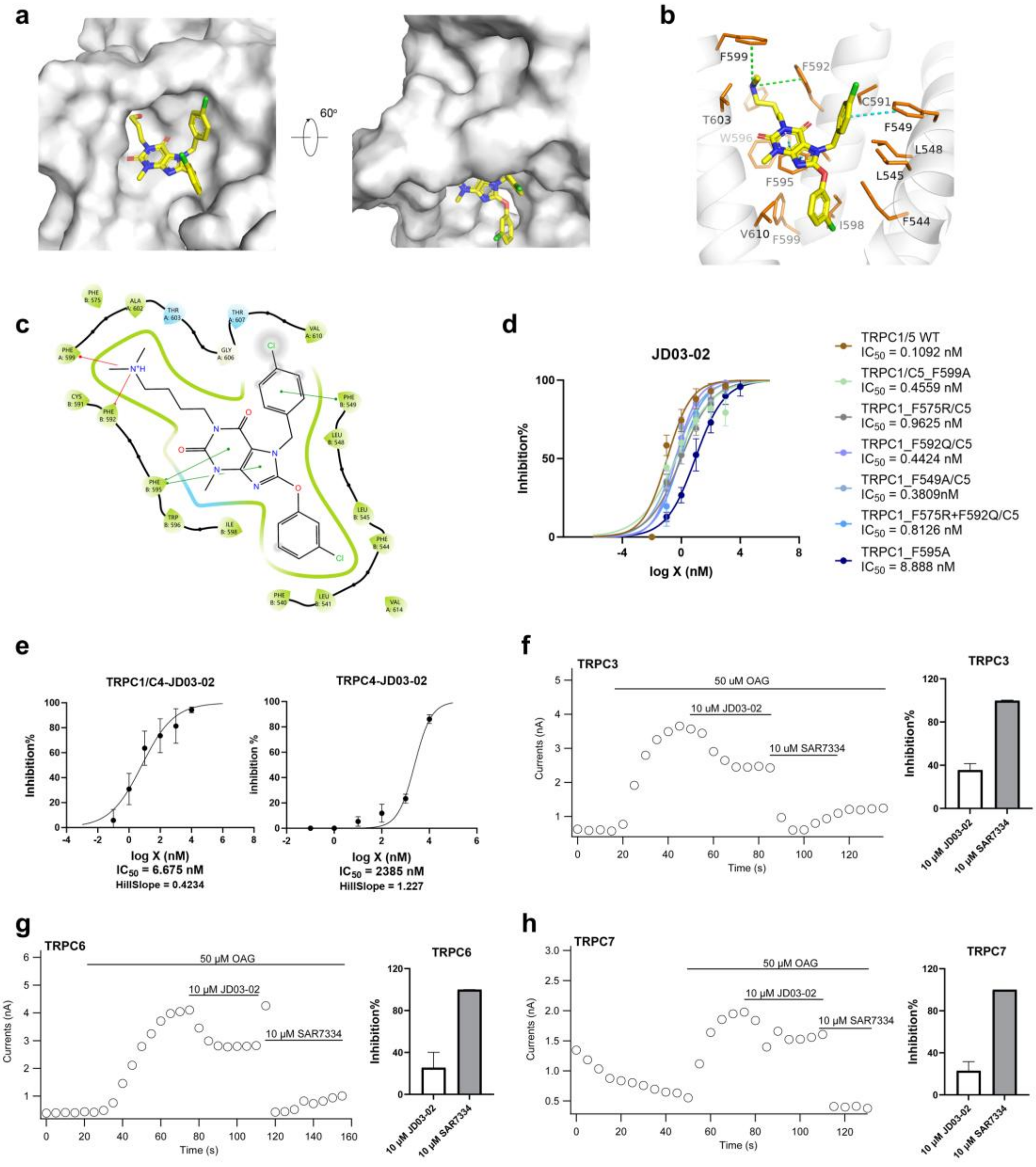

Supplementary Figure 9. Molecular docking of JD03-02 to the TRPC1/5 heteromer. **(a)** Surface representation of the JD03-02 binding pocket in the TRPC1/5 heteromer from different angles. **(b)** Structural analysis of the interactions between the JD03-02 and residues in the TRPC1/5 heteromer. **(c)** Cartoon representation of the interaction between JD03-02 and the TRPC1/5 heteromer. **(d)** Inhibitory effect of JD03-02 on various TRPC1 or TRPC5 mutations within the TRPC1/5 heteromer, as measured by whole-cell patch-clamp assay. Error bars represent the standard error of the mean (SEM, n = 6 independent cells). **(e)** Inhibitory effect of JD03-02 on TRPC1/4 heteromer and TRPC4 homomer, as measured by whole-cell patch-clamp assays. Error bars represent the standard error of the mean (SEM, n = 5 independent cells). **(f)** Inhibitory effect of JD03-02 on TRPC3 homomer with 10 μM, as measured by whole-cell patch-clamp assays. Error bars represent the standard error of the mean (SEM, n = 5 independent cells). **(g)** Inhibitory effect of JD03-02 on TRPC6 homomer with 10 μM, as measured by whole-cell patch-clamp assays. Error

bars represent the standard error of the mean (SEM, n = 5 independent cells). **(h)** Inhibitory effect of JD03-02 on TRPC7 homomer with 10 μM, as measured by whole-cell patch-clamp assays. Error bars represent the standard error of the mean (SEM, n = 5 independent cells).

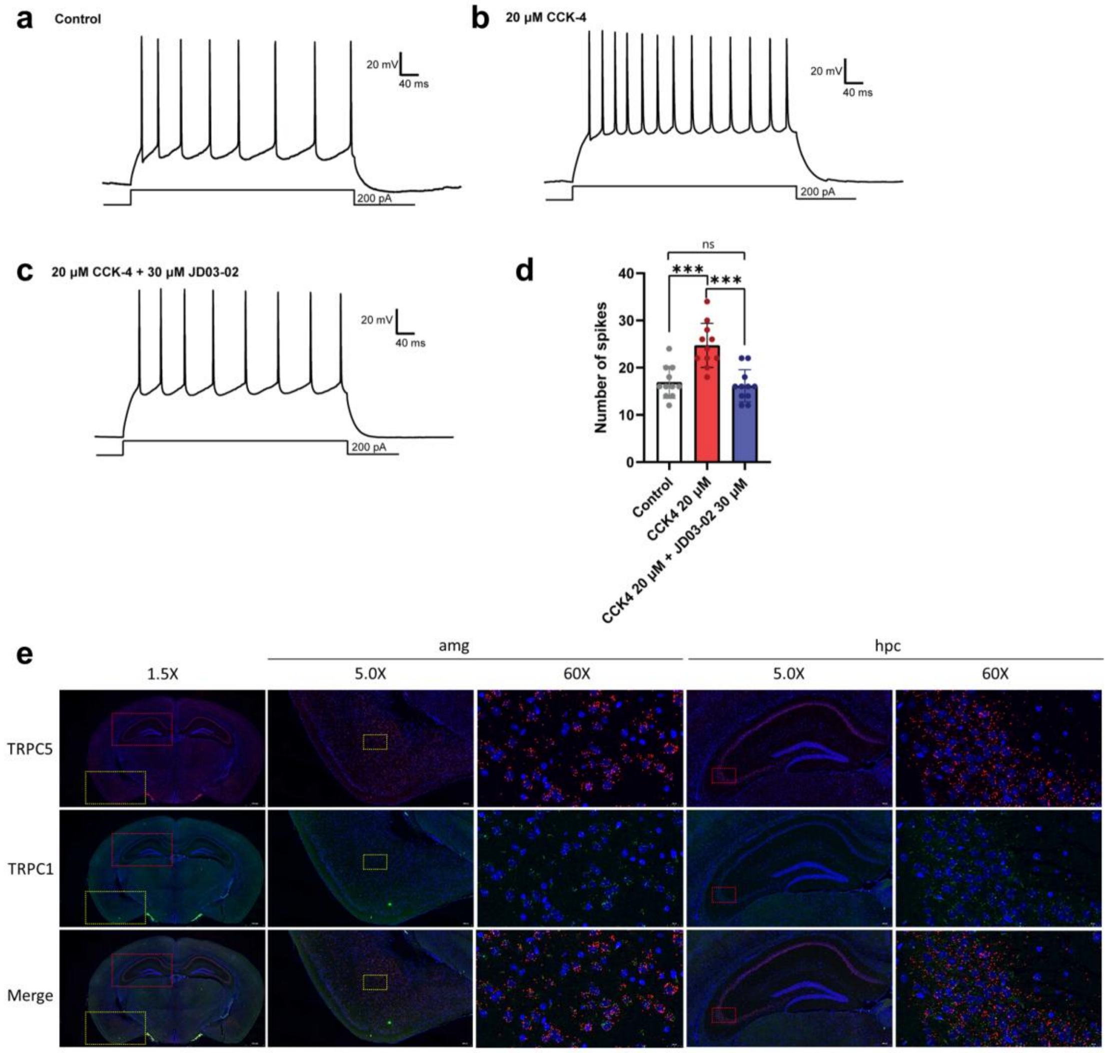

Supplementary Figure 10. The TRPC1/5-selective inhibitor JD03-02 effectively counteracts CCK-4-induced neuronal hyperexcitability in the lateral amygdala (LA). (a-c) Responses of LA neurons in acute brain slices to a series of current injections (200 pA, 500 ms) recorded in current-clamp mode from the Control, CCK-4 (20 μM), and CCK-4 (20 μM) + JD03-02 (30 μM) groups, respectively. **(d)** Quantification of action potential numbers evoked at the 200 pA step. Data are presented as mean ± SEM (n = 11 neurons per group). *p* values were determined by one-way ANOVA followed by Tukey's post hoc test using SPSS; ***$p < 0.001$, “ns” indicates no significant difference. Exact *p* values are provided in Source Data. **(e)** RNAscope assay was performed to characterize the expression patterns of TRPC5 and TRPC1 in the mouse brain. The yellow boxes show the magnified views of the amygdala region in sequence (amg, amygdala); the red boxes show the magnified views of the hippocampal region in sequence (hpc, hippocampus). Scale bar: 1000 μm for 1.5× magnification; 200 μm for 5.0× magnification; 20 μm for 60× magnification.

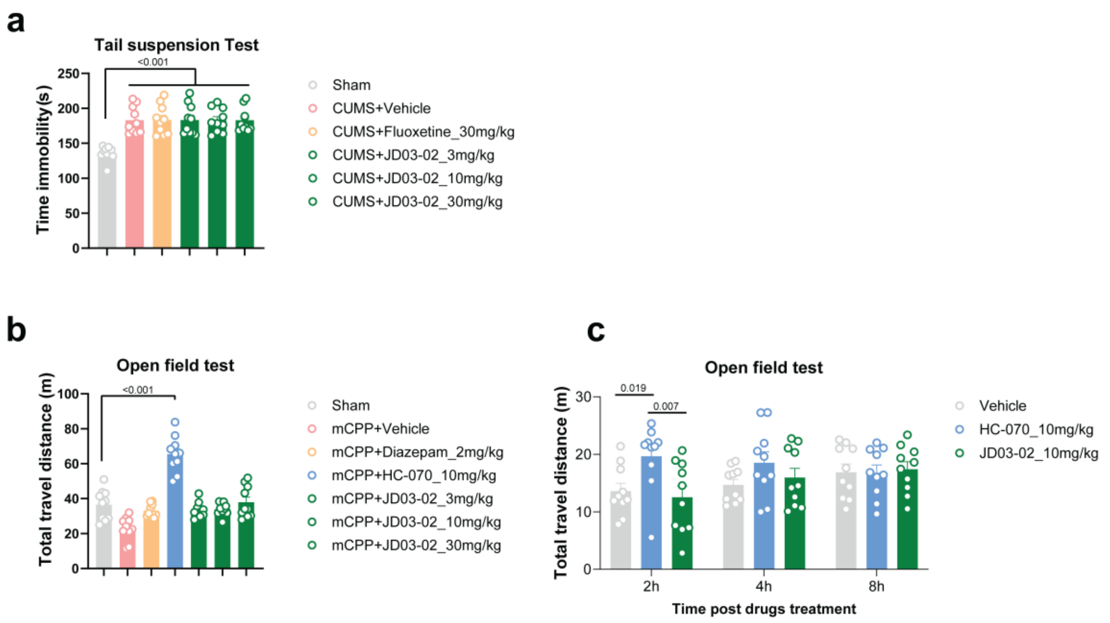

Supplementary Figure 11. JD03-02 does not affect locomotor activity in both anxiety and healthy mice, unlike HC-070. (a) Tail suspension test (TST) was performed to validate the induction of significant depressive-like behaviors in mice after 28 days of CUMS. n = 10, data are presented as mean ± SEM. Statistical significance was determined by One-way ANOVA with Welch's correction for multiple comparisons, for each group vs. Sham with p values shown. **(b)** Total distance traveled in the Open Field Test was quantified to assess the effect of single oral administration of JD03-02 and HC-070 on locomotor activity in mCPP-induced anxiety-like mice. Diazepam, vehicle, and sham groups were set as positive control, vehicle control, and sham control, respectively. n = 10, data are presented as mean ± SEM. Statistical significance was determined by One-way ANOVA with Welch's correction for multiple comparisons, for each group vs. Sham with *p* values shown. **(c)** Total distance traveled in the Open Field Test was quantified at 2, 4, and 8h post-treatment with JD03-02, HC-070, or vehicle, to evaluate their effects on locomotor activity in healthy *C57BL/6J* mice. n = 10, data are presented as mean ± SEM. Statistical significance was determined by One-way ANOVA with Fisher's LSD test for multiple comparisons as indicated by the line above the column, with *p* values shown.

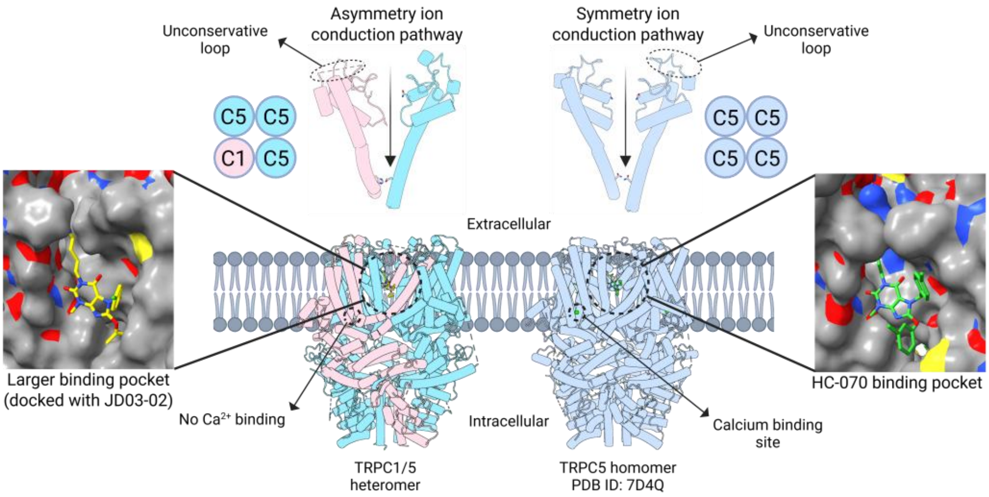

Supplementary Figure 12. Comparative analysis of TRPC1/5 heteromer and TRPC5 homomer features. Created in BioRender. Wan, S. (2026) https://BioRender.com/ah3hgpw

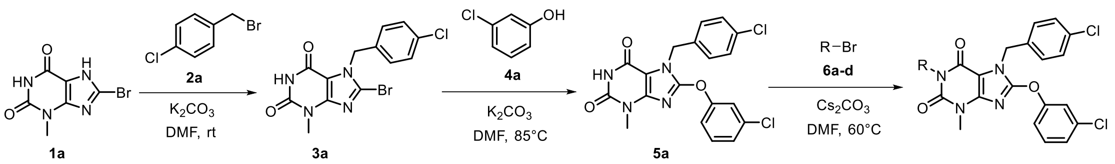

Supplementary Figure 13. Synthesis of compounds H01-H13.

Supplementary Table 1: Cryo-EM data collection, refinement and validation statistics.

Structuer	TRPC1/5
EMDB accession code	(EMD-62060)
PDB accession code	(PDB 9K4I)
**Data collection and processing**	
Magnification	105,000
Voltage (kV)	300
Electron exposure (e–/Å^2)	54
Defocus range (μm)	-1.0 ~ -2.0
Pixel size (Å)	0.83
Symmetry imposed	*C1*
Initial particle images (#)	2,411,504
Final particle images (#)	143,788
Map resolution (Å)	2.84
FSC threshold	0.143
**Refinement**	
Initial model used (PDB code)	6AEI
Model resolution (Å)	2.89
FSC threshold	0.143
**Model composition**	
Non-hydrogen atoms	22,192
Protein residues	2709
Ligands	10
**B factors (Å^2)**	
Protein	72.71
Ligand	76.54
r.m.s. deviations	
Bond lengths (Å)	0.008
Bond angles (°)	0.944
**Validation**	
MolProbity score	2.01
Clashscore	17
Poor rotamers (%)	0
Ramachandran plot	
Favored (%)	94.46
Allowed (%)	5.54
Disallowed (%)	0

Suppelmentary Table 2: Pharmacokinetics parameters of JD03-02 after p.o administration in mouse.

Compound	Matrix	T_{max} (h)	C_{max} (ng/mL or ng/g)	AUC_{last} (h*ng/mL or h*ng/g)	$AUC_{last\ brain}$/$AUC_{last\ heart\ plasma}$	$AUC_{last\ heart\ plasma}$/$AUC_{last\ portal\ vein\ plasma}$	$C_{last\ brain}$/$C_{max\ heart\ plasma}$
	heart plasma	4.00	318	778			
JD03-02	portal vein plasma	0.25	493	1332	0.67	0.58	0.83
	brain	4.00	265	524			

Suppelmentary Table 3: Pharmacokinetics parameters of JD03-02 after 10mg/kg p.o and 2mg/kg i.v. administration in mouse.

Administration	Animal No.	$T_{1/2}$	T_{max}	C_{max}	AUC_{last}	AUC_{INF_obs}	$CL_{\_obs}$	MRT_{INF_obs}	$Vss_{\_obs}$	F
		(h)	(h)	(ng/mL)	(h*ng/mL)	(h*ng/mL)	(mL/min/kg)	(h)	(mL/kg)	(%)
p.o.	1	2.86	1.00	256	1294	1299	-	4.73	-	49.8
	2	3.35	1.00	205	1532	1549	-	6.50	-	
	3	2.48	1.00	143	746	800	-	4.58	-	
	**Mean**	**2.90**	**1.00**	**201**	**1191**	**1216**	**-**	**5.27**	**-**	
	**SD**	**0.44**	**0.00**	**57**	**403**	**381**	**-**	**1.06**	**-**	
i.v.	4	3.52	-	-	385	469	71.0	4.455	18984	
	5	2.06	-	-	416	447	74.6	2.895	12955	
	6	3.69	-	-	634	640	52.1	4.460	13949	
	**Mean**	**3.09**	**-**	**-**	**478**	**519**	**65.9**	**3.937**	**15296**	
	**SD**	**0.90**	**-**	**-**	**135**	**105**	**12.1**	**0.902**	**3233**	

General procedures for the synthesis of compounds.

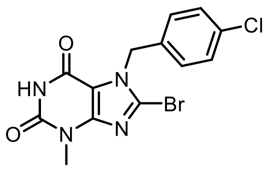

8-bromo-7-(4-chlorobenzyl)-3-methyl-3,7-dihydro-1*H*-purine-2,6-dione (3a). To a solution of 8-bromo-3-methyl-3,7-dihydro-1H-purine-2,6-dione (**1a**, 5.0 g, 20.40 mmol, 1.0 eq) in DMF (100.0 mL) was added K_2CO_3 (3.1 g, 22.45 mmol, 1.1 eq), and 1-(bromomethyl)-4-chlorobenzene (**2a**, 4.6 g, 22.45 mmol, 1.1 eq) under nitrogen atmosphere. Then mixture was stirred at room temperature for 3 h. The mixture was poured into ice water (500.0 mL). The precipitate formed was collected by filtration, washed with water, dried in vacuo under reduced pressure to give residue The residue was triturated with EtOAc (60.0 mL) and filtered to give **3a** as a white solid (7.0 g, 92.8%). LC-MS: 368.9, 370.9 ($[M+H]^+$).

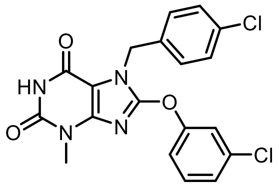

7-(4-chlorobenzyl)-8-(3-chlorophenoxy)-3-methyl-3,7-dihydro-1*H*-purine-2,6-dione (5a). To a solution of **3a** (3.0 g, 8.12 mmol, 1.0 eq) in DMF (60.0 mL) was added K_2CO_3 (1.2 g, 8.93 mmol, 1.1 eq), and 3-chlorophenol (**4a**, 1.25 g, 9.74 mmol, 1.2 eq) under nitrogen atmosphere. Then mixture was stirred at 85°C for 15 h. The cold mixture was poured into ice water (200.0 mL). The precipitate formed was collected by filtration, washed with water, dried in vacuo under reduced pressure to give **5a** as a white solid (3.0 g, 88.6%).

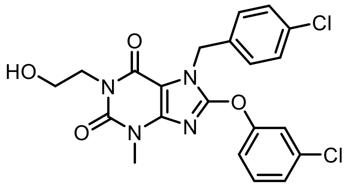

7-(4-chlorobenzyl)-8-(3-chlorophenoxy)-1-(2-hydroxyethyl)-3-methyl-3,7-dihydro-1*H*-purine-2,6-dione (7a). To a solution of **5a** (100 mg, 0.24 mmol, 1.0 eq)

in DMF (3.0 mL) was added Cs_2CO_3 (102 mg, 0.31 mmol, 1.3 eq), and 2-bromoethan-1-ol (**6a**, 36 mg, 0.29 mmol, 1.2 eq) under nitrogen atmosphere. Then mixture was stirred at 60°C for 15 h. The mixture was diluted with water (10 mL) and extracted with EtOAc (10 mL × 3). The combined organic phase was washed with water (10 mL × 3) and brine (10 mL), dried over Na_2SO_4, filtered and concentrated under reduced pressure to give a residue. The residue was purified by prep-TLC (DCM / EtOAc = 1:1) to give **7a** as a white solid (70 mg, 63.3%). LC-MS: 461.1 ($[M+H]^+$).

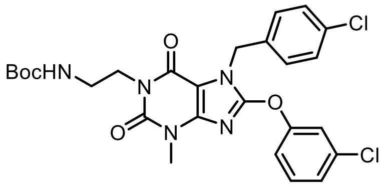

tert-butyl (2-(7-(4-chlorobenzyl)-8-(3-chlorophenoxy)-3-methyl-2,6-dioxo-2,3,6,7-tetrahydro-1*H*-purin-1-yl)ethyl)carbamate (7b). To a solution of **5a** (625 mg, 1.5 mmol, 1.0 eq) in DMF (15.0 mL) was was added K_2CO_3 (415 mg, 3.00 mmol, 2.0 eq), and tert-butyl (2-bromoethyl)carbamate (**6b**, 360 mg, 1.6 mmol, 1.05 eq) under nitrogen atmosphere. Then mixture was stirred at 60 °C for 15 h. The combined organic phase was washed with water (15 mL × 3) and brine (20 mL), dried over Na_2SO_4, filtered and concentrated under reduced pressure to give a residue. The residue was purified by silica gel column chromatography (DCM / MeOH = 30:1) to give **7b** as a yellow solid (390 mg, 46.3%). LC-MS: 460.1 ($[M\text{-}Boc+H]^+$).

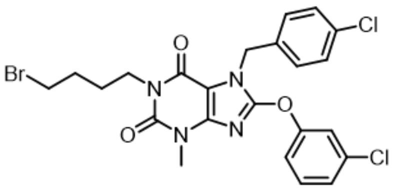

1-(4-bromobutyl)-7-(4-chlorobenzyl)-8-(3-chlorophenoxy)-3-methyl-3,7-dihydro-1*H*-purine-2,6-dione (7c). To a solution of **5a** (500 mg, 1.20 mmol, 1.0 eq) in DMF (5 mL) was added K_2CO_3 (182 mg, 1.32 mmol, 1.1 eq) and 1,4-dibromobutane (**6c**, 259 mg, 1.20 mmol, 1.0 eq). The mixture was stirred at 50°C for 1 h. The mixture was diluted with water (8 mL) and extracted with EtOAc (8 mL x 2). The combined organic phase was washed with water (8 mL × 2), brine (8 mL), dried over Na_2SO_4, filtered and concentrated under reduced pressure to give a residue. The residue was purified by

silica gel column chromatography (Petroleum ether: EtOAc = 20: 1) to give **7c** as a colorless oil (370 mg, 55.9%). LC-MS: 550.6 ([M+H]$^+$).

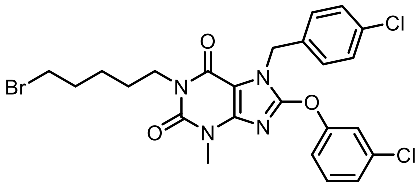

1-(5-bromopentyl)-7-(4-chlorobenzyl)-8-(3-chlorophenoxy)-3-methyl-3,7-dihydro-1*H*-purine-2,6-dione (7d). To a solution of **5a** (100 mg, 0.24 mmol, 1.0 eq) in DMF (1.0 mL) was added 1,5-dibromopentane (**6d**, 55 mg, 0.24 mmol, 1.0 eq) and K_2CO_3 (36 mg, 0.26 mmol, 1.1 eq). The mixture was stirred at 50°C for 15 h. The mixture was diluted with water (10 mL) and extracted with EtOAc (10 mL × 2). The combined organic phase was washed with water (10 mL × 2) and brine (10 mL × 2), dried over Na_2SO_4, filtered and concentrated under reduced pressure to give a residue. The residue was purified by Prep-TLC (Petroleum ether / EtOAc = 3:1) to give **7d** as a colorless oil（70 mg, 51.6%). LC-MS: 565.1 ([M+H]$^+$).

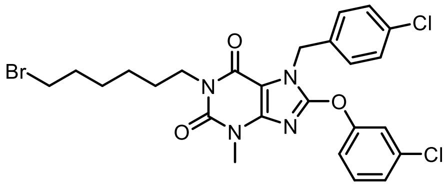

1-(6-bromohexyl)-7-(4-chlorobenzyl)-8-(3-chlorophenoxy)-3-methyl-3,7-dihydro-1H-purine-2,6-dione (7e). To a solution of **5a** (100 mg, 0.24 mmol, 1.0 eq) in DMF (1.0 mL) was added 1,6-dibromohexane (**6e**, 58 mg, 0.24 mmol, 1.0 eq) and K_2CO_3 (36 mg, 0.26 mmol, 1.1 eq). The mixture was stirred at 50°C for 15 h. The mixture was diluted with water (10 mL) and extracted with EtOAc (10 mL × 2). The combined organic phase was washed with water (10 mL × 2) and brine (10 mL × 2), dried over Na_2SO_4, filtered and concentrated under reduced pressure to give a residue. The residue was purified by Prep-TLC (Petroleum ether / EtOAc = 3:1) to give **7e** (70 mg, 50.4%) as a colorless oil. LC-MS: 579.0 ([M+H]$^+$).

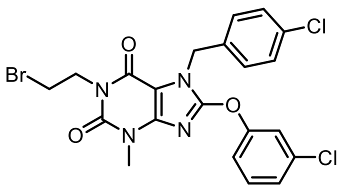

1-(2-bromoethyl)-7-(4-chlorobenzyl)-8-(3-chlorophenoxy)-3-methyl-3,7-dihydro-1*H*-purine-2,6-dione (8a). To a solution of **7a** (70 mg, 0.15 mmol, 1.0 eq) in DCM (3.0 mL) was added PPh_3 (60 mg, 0.23 mmol, 1.5 eq), and CBr_4 (76 mg, 0.23 mmol, 1.5 eq) under nitrogen atmosphere. Then mixture was stirred at room temperature for 15 h. The mixture was diluted with water (10 mL) and extracted with DCM (10 mL × 3). The combined organic phase was washed with brine (10 mL), dried over Na_2SO_4, filtered and concentrated under reduced pressure to give a residue. The residue was purified by prep-TLC (Petroleum ether / EtOAc = 2:1) to give **8a** (60 mg, 75.0%) as a white solid. LC-MS: 522.9 ($[M+H]^+$).

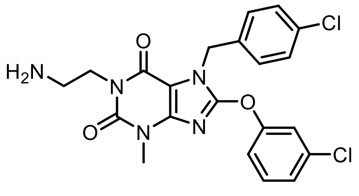

1-(2-aminoethyl)-7-(4-chlorobenzyl)-8-(3-chlorophenoxy)-3-methyl-3,7-dihydro-1*H*-purine-2,6-dione (8b). To a solution of **7b** (300 mg, 0.54 mmol, 1.0 eq) in DCM (5.0 mL) was added HCl/Dioxane (4.0 M) (1.3 mL, 5.35 mmol, 10.0 eq). The mixture was stirred at room temperature for 2 h. The mixture was concentrated to give **8b** (270 mg, crude) as a white solid. LC-MS: 460.0 ($[M+H]^+$).

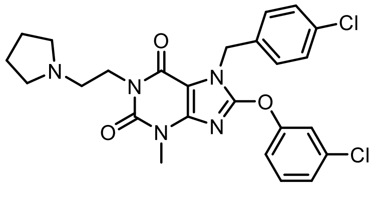

7-(4-chlorobenzyl)-8-(3-chlorophenoxy)-3-methyl-1-(2-(pyrrolidin-1-yl)ethyl)-3,7-dihydro-1*H*-purine-2,6-dione (H01). To a solution of **8a** (30 mg, 0.06 mmol, 1.0 eq) in Dioxane (2.0 mL) was added pyrrolidine (41 mg, 0.57 mmol, 10.0 eq) under nitrogen atmosphere. Then mixture was stirred at room temperature for 15 h. The mixture was concentrated under reduced pressure to give a residue. The residue was purified by prep-TLC (DCM / MeOH = 10:1) to give **H01** as a white solid (3.3 mg,

11.3%). ^{1}H NMR (300 MHz, MeOD-d_4): δ 7.46-7.38 (m, 3H), 7.38-7.28 (m, 4H), 7.26-7.21 (m, 1H), 5.46 (s, 2H), 4.24 (t, J = 6.3 Hz, 2H), 3.40 (s, 3H), 3.05 (t, J = 6.3 Hz, 2H), 2.97 (s, 4H), 1.97-1.85 (m, 4H). ^{13}C NMR (75 MHz, DMSO-d_6): δ 154.42, 154.10, 152.95, 151.25, 146.30, 135.63, 134.19, 133.14, 131.87, 130.11, 129.22, 126.52, 120.48, 119.07, 103.28, 53.95, 52.43, 46.43, 36.73, 30.19, 23.30. LC-MS: 514.1 ([M+H]$^+$). HPLC RT: 4.63 min, purity 99.2%.

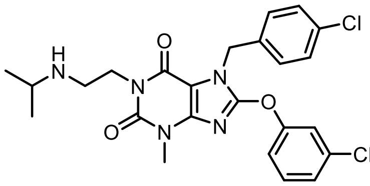

7-(4-chlorobenzyl)-8-(3-chlorophenoxy)-1-(2-(isopropylamino)ethyl)-3-methyl-3,7-dihydro-1*H*-purine-2,6-dione (H02). To a solution of **8a** (30 mg, 0.06 mmol, 1.0 eq) in Dioxane (2.0 mL) was added isopropylamine (101 mg, 1.71 mmol, 30.0 eq) under nitrogen atmosphere. The mixture was heated under microwave irradiation at 130°C for 2 h The mixture was concentrated under reduced pressure to give a residue. The residue was purified by prep-TLC (DCM / MeOH = 10:1) to give **H02** as a white solid (7 mg, 24.4%). ^{1}H NMR (300 MHz, MeOD-d_4): δ 7.48-7.41 (m, 3H), 7.40-7.30 (m, 4H), 7.29-7.23 (m, 1H), 5.49 (s, 2H), 4.26 (t, J = 6.0 Hz, 2H), 3.43 (s, 3H), 3.27-3.16 (m, 1H), 3.13 (t, J = 6.0 Hz, 2H), 1.23 (d, J = 6.3 Hz, 6H). ^{13}C NMR (101 MHz, DMSO-d_6): δ 154.56, 154.09, 152.95, 151.46, 146.32, 135.61, 134.20, 133.19, 131.90, 130.21, 129.22, 126.55, 120.47, 119.07, 103.33, 49.72, 46.47, 42.65, 37.84, 30.21, 19.21. LC-MS: 502.1 ([M+H]$^+$). HPLC RT: 4.63 min, purity 97.3%.

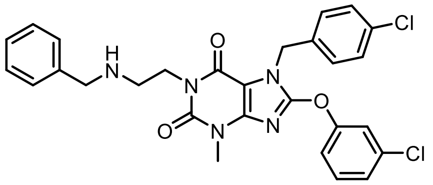

1-(2-(benzylamino)ethyl)-7-(4-chlorobenzyl)-8-(3-chlorophenoxy)-3-methyl-3,7-dihydro-1*H*-purine-2,6-dione (H03). To a solution of **8b** (50 mg crude, 0.10 mmol, 1.0 eq) in DCE (3.0 mL) was added TEA (15 mg, 0.15 mmol, 1.5 eq). The mixture was stirred at room temperature for 10 min. Then AcOH (12 mg, 0.20 mmol, 2.0 eq), benzaldehyde (12 mg, 0.13 mmol, 1.3 eq) and $NaBH(OAc)_3$ (16 mg, 0.15 mmol, 1.5

eq) was added and stirred for 15 h. The mixture was quenched by the addition of saturated aqueous Na_2CO_3 solution (10 mL) and extracted with DCM (10 mL × 2). The combined organic phase was washed with brine (10 mL), dried over Na_2SO_4, filtered and concentrated under reduced pressure to give a residue. The residue was purified by prep-TLC (DCM / MeOH = 15:1) to give **H03** as a yellow solid (6 mg, 10.9%). ^{1}H NMR (300 MHz, DMSO-d_6): δ 7.53-7.46 (m, 2H), 7.42-7.22 (m, 11H), 5.42 (s, 2H), 4.03 (t, J = 6.6 Hz, 2H), 3.78 (s, 2H), 3.28 (s, 3H), 2.79 (t, J = 8.1 Hz, 2H). ^{13}C NMR (75 MHz, DMSO-d_6): δ 154.54, 154.18, 152.83, 151.22, 146.05, 135.68, 134.18, 133.12, 131.84, 130.11, 129.22, 128.75, 128.61, 126.45, 120.45, 119.04, 103.16, 51.98, 50.80, 49.69, 46.39, 30.10. LC-MS: 550.1 ([M+H]$^+$). HPLC RT: 4.88 min, purity 97.9%.

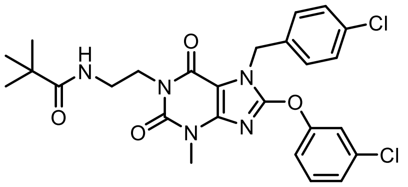

N-(2-(7-(4-chlorobenzyl)-8-(3-chlorophenoxy)-3-methyl-2,6-dioxo-2,3,6,7-tetrahydro-1*H*-purin-1-yl)ethyl)pivalamide (H04). To a solution of **8b** (50 mg, 0.11 mmol, 1.0 eq) in DCM (3.0 mL) was added TEA (55 mg, 0.55 mmol, 5.0 eq) and PivCl (16 mg, 0.13 mmol, 1.2 eq). The mixture was stirred at room temperature for 2 h. The mixture was diluted with water (5 mL) and extracted with DCM (5 mL × 2). The combined organic phase was washed with brine (5 mL), dried over Na_2SO_4, filtered and concentrated under reduced pressure to give a residue. The residue was purified by prep-TLC (DCM / EtOAc = 1:1) to give **H04** as a white solid (32 mg, 54.1%). ^{1}H NMR (300 MHz, DMSO-d_6): δ 7.53-7.42 (m, 3H), 7.41-7.31 (m, 6H), 5.42 (s, 2H), 3.96 (t, J = 5.4 Hz, 2H), 3.33-3.31 (m, 2H), 3.29 (s, 3H), 0.98 (s, 9H). ^{13}C NMR (75 MHz, DMSO-d_6): δ 177.83, 154.64, 154.15, 152.73, 151.27, 145.92, 135.66, 134.18, 133.10, 131.84, 130.09, 129.20, 126.46, 120.46, 119.06, 103.18, 46.36, 39.17, 38.31, 37.41, 30.04, 27.77. LC-MS: 544.1 ([M+H]$^+$). HPLC RT: 5.58 min, purity 98.5%.

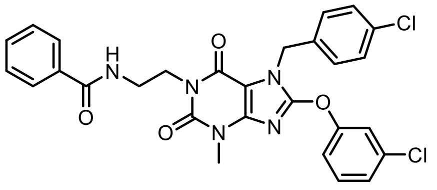

N-(2-(7-(4-chlorobenzyl)-8-(3-chlorophenoxy)-3-methyl-2,6-dioxo-2,3,6,7-tetrahydro-1*H*-purin-1-yl)ethyl)benzamide (H05). To a solution of **8b** (50 mg, 0.11 mmol, 1.0 eq) in DCM (3.0 mL) was added TEA (55 mg, 0.55 mmol, 5.0 eq) and Benzoyl chloride (19 mg, 0.13 mmol, 1.2 eq). The mixture was stirred at room temperature for 2 h. The mixture was diluted with water (5 mL) and extracted with DCM (5 mL × 2). The combined organic phase was washed with brine (5 mL), dried over Na_2SO_4, filtered and concentrated under reduced pressure to give a residue. The residue was purified by prep-TLC (DCM / EtOAc = 1:1) to give **H05** as a white solid (45 mg, 73.4%). ^{1}H NMR (400 MHz, DMSO-d_6): δ 8.48 (t, J = 5.8 Hz, 1H), 7.74 (d, J = 7.2, 2H), 7.56-7.22 (m, 11H), 5.38 (s, 2H), 4.10 (t, J = 5.6 Hz, 2H), 3.56-3.49 (m, 2H), 3.29 (s, 3H). ^{13}C NMR (75 MHz, DMSO-d_6): δ 166.98, 154.70, 154.13, 152.74, 151.29, 145.99, 135.59, 135.24, 134.17, 133.09, 131.84, 131.42, 130.12, 129.14, 128.65, 127.59, 126.46, 120.46, 119.05, 103.15, 46.31, 39.18, 37.76, 30.07. LC-MS: 564.1 ($[M+H]^+$). HPLC RT: 5.55 min, purity 95.8%.

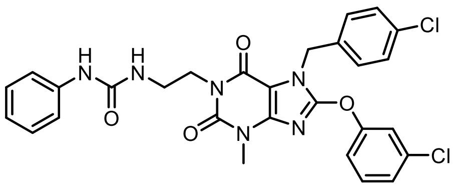

1-(2-(7-(4-chlorobenzyl)-8-(3-chlorophenoxy)-3-methyl-2,6-dioxo-2,3,6,7-tetrahydro-1*H*-purin-1-yl)ethyl)-3-phenylurea (H06). To a solution of **8b** (50 mg, 0.11 mmol, 1.0 eq) and TEA (51 mg, 0.50 mmol, 5.0 eq) in DCM (3.0 mL) was added $(COCl_2)_3$ (15 mg, 0.05 mmol, 0.5 eq) in DCM (1.0 mL). The mixture was stirred at room temperature for 0.5 h. Then aniline (12 mg, 0.13 mmol, 1.3 eq) in DCM (1.0 mL) was added and stirred for 14.5 h. The mixture was diluted with water (10 mL) and extracted with DCM (10 mL × 2). The combined organic phase was washed with brine (10 mL), dried over Na_2SO_4, filtered and concentrated under reduced pressure to give a residue. The residue was purified by prep-TLC (DCM / EtOAc = 1:1) to give **H06** as a yellow solid (10 mg, 17.3%). ^{1}H NMR (300 MHz, DMSO-d_6): δ 8.45 (s, 1H), 7.53-7.44 (m, 2H), 7.43-7.36 (m, 3H), 7.35-7.27 (m, 5H), 7.19(t, J = 7.5 Hz, 2H), 6.87 (t, J = 7.2 Hz, 1H), 6.14 (t, J = 5.7 Hz, 1H), 5.40 (s, 2H), 4.02 (t, J = 6.0 Hz, 2H), 3.41-3.34

(m, 2H), 3.28 (s, 3H). ^{13}C NMR (75 MHz, DMSO-d_6): δ 155.76, 154.63, 154.13, 152.77, 151.24, 146.03, 140.97, 135.60, 134.17, 133.11, 131.83, 130.14, 129.19, 129.03, 126.45, 121.45, 120.45, 119.03, 118.13, 103.09, 45.89, 41.07, 37.88, 30.68. LC-MS: 579.1 ([M+H]$^+$). HPLC RT: 5.08 min, purity 98.9%.

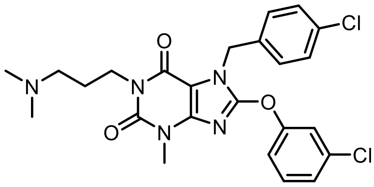

7-(4-chlorobenzyl)-8-(3-chlorophenoxy)-1-(3-(dimethylamino)propyl)-3-methyl-3,7-dihydro-1*H*-purine-2,6-dione (H07). To a solution of **5a** (112 mg, 0.27 mmol, 1.0 eq) in DMF (2.0 mL) was added K_2CO_3 (56 mg, 0.41 mmol, 1.5 eq), and 3-bromo-N,N-dimethylpropan-1-amine (**6f**, 38 mg, 0.30 mmol, 1.1 eq) under nitrogen atmosphere. Then mixture was stirred at 85°C for 15 h. The mixture was diluted with water (10 mL) and extracted with EtOAc (10 mL × 2). The combined organic phase was washed with water (10 mL × 3) and brine (10 mL), dried over Na_2SO_4, filtered and concentrated under reduced pressure to give a residue. The residue was purified by prep-TLC (DCM / MeOH = 10:1) to give **H07** as a white solid (53 mg, 38.8%). ^{1}H NMR (300 MHz, DMSO-d_6): δ 7.54-7.47 (m, 2H), 7.44-7.31 (m, 6H), 5.43 (s, 2H), 3.92 (t, J = 6.9 Hz, 2H), 3.30 (s, 3H), 2.81 (t, J = 6.9 Hz, 2H), 2.53 (s, 6H), 1.94-1.79 (m, 2H). ^{13}C NMR (75 MHz, DMSO-d_6): δ 154.44, 154.15, 152.90, 151.11, 146.15, 135.67, 134.18, 133.14, 131.86, 130.12, 129.25, 126.49, 120.47, 119.06, 103.12, 55.40, 46.42, 43.37, 38.64, 30.14, 24.17. LC-MS: 502.1 ([M+H]$^+$). HPLC RT: 3.91 min, purity 99.1%.

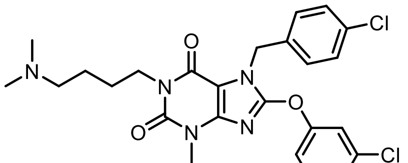

7-(4-chlorobenzyl)-8-(3-chlorophenoxy)-1-(4-(dimethylamino)butyl)-3-methyl-3,7-dihydro-1*H*-purine-2,6-dione (H08). To a solution of **7c** (370 mg, 0.67 mmol, 1.0 eq) in THF (2 mL) was added dimethylamine in THF (2.0 M) (2 mL). The mixture was stirred at 50°C for 15 h. The mixture was diluted with water (8 mL) and extracted with EtOAc (8 mL × 2). The combined organic phase was washed with brine (8 mL), dried

over Na_2SO_4, filtered and concentrated under reduced pressure to give a residue. The residue was purified by silica gel column chromatography (DCM: MeOH = 20: 1) to give **H08** as a white solid (211 mg, 61.0%). ^{1}H NMR (300 MHz, DMSO-d_6): δ 7.53-7.33 (m, 8H), 5.42 (s, 2H), 3.87 (t, J = 6.9 Hz, 2H), 3.29 (s, 3H), 2.34 (t, J = 7.2 Hz, 2H), 2.20 (s, 6H), 1.60-1.49 (m, 2H), 1.47-1.36 (m, 2H). ^{13}C NMR (75 MHz, DMSO-d_6): δ 154.34, 154.16, 152.83, 150.98, 145.99, 135.70, 134.17, 133.11, 131.79, 130.05, 129.21, 126.42, 120.48, 119.05, 103.03, 58.93, 46.37, 45.25, 40.75, 30.06, 25.85, 24.55. LC-MS: 515.8 ($[M+H]^+$). HPLC RT: 4.70 min, purity 99.3%.

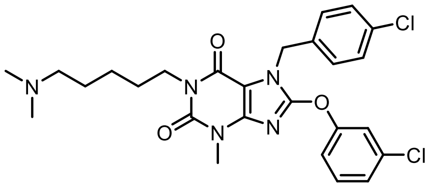

7-(4-chlorobenzyl)-8-(3-chlorophenoxy)-1-(5-(dimethylamino)pentyl)-3-methyl-3,7-dihydro-1H-purine-2,6-dione (H09). To a solution of **7d** (70 mg, 0.12 mmol, 1.0 eq) in dioxane (1.0 mL) was added 2M dimethylamine in THF (1.0 mL). The mixture was stirred at 50°C for 15 h. The mixture was concentrated under reduced pressure to give a residue. The residue was purified by Prep-TLC (DCM / MeOH = 10:1) to give **H09** as a white solid (47.1 mg, 62.3%). ^{1}H NMR (400 MHz, DMSO-d_6): δ 9.78 (brs, 1H), 7.59-7.27 (m, 8H), 5.43 (s, 2H), 3.88 (t, J = 7.2 Hz, 2H), 3.30 (s, 3H), 3.03-2.86 (m, 2H), 2.70 (s, 6H), 1.77-1.48 (m, 4H), 1.37-1.15 (m, 2H). ^{13}C NMR (75 MHz, DMSO-d_6): δ 154.36, 154.16, 152.88, 151.02, 146.03, 135.71, 134.17, 133.12, 131.83, 130.05, 129.25, 126.46, 120.48, 119.07, 103.06, 56.96, 46.40, 42.57, 40.84, 30.10, 27.56, 23.93, 23.87. LC-MS: 530.2 ($[M+H]^+$). HPLC RT: 4.11 min, purity 95.5%.

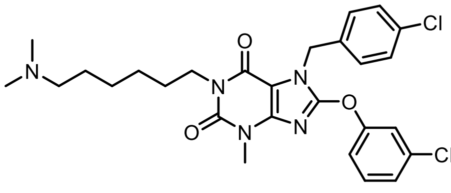

7-(4-chlorobenzyl)-8-(3-chlorophenoxy)-1-(6-(dimethylamino)hexyl)-3-methyl-3,7-dihydro-1H-purine-2,6-dione (H10). To a solution of **7e** (70 mg, 0.12 mmol, 1.0 eq) in dioxane (1.0 mL) was added dimethylamine in THF (2 M, 1.0 mL). The mixture was stirred at 50 °C for 15 h. The mixture was concentrated under reduced pressure to

give a residue. The residue was purified by Prep-TLC (DCM / MeOH = 15:1) and Prep-HPLC (acetonitrile with 0.1% FA in water, 30% to 70%) to give **H10** as a colorless oil (28.6 mg, 40.1%). ^{1}H NMR (400 MHz, DMSO-d_6): δ 7.54-7.46 (m, 2H), 7.44-7.30 (m, 6H), 5.42 (s, 2H), 3.91-3.78 (m, 2H), 3.29 (s, 3H), 2.40-2.30 (m, 2H), 2.23 (s, 6H), 1.60-1.49 (m, 2H), 1.47-1.36 (m, 2H), 1.34-1.18 (m, 4H). ^{13}C NMR (75 MHz, DMSO-d_6): δ 154.32, 154.15, 152.82, 150.96, 145.96, 135.69, 134.16, 133.12, 131.80, 130.06, 129.21, 126.42, 120.46, 119.04, 103.03, 58.89, 46.35, 44.87, 40.81, 30.06, 27.87, 26.79, 26.64, 26.61. LC-MS: 544.2 ($[M+H]^+$). HPLC RT: 4.15 min, purity 99.6%.

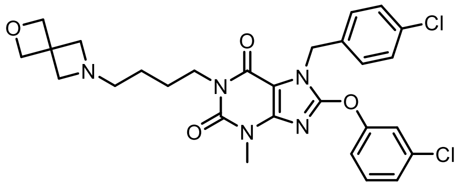

1-(4-(2-oxa-6-azaspiro[3.3]heptan-6-yl)butyl)-7-(4-chlorobenzyl)-8-(3-chlorophenoxy)-3-methyl-3,7-dihydro-1*H*-purine-2,6-dione (H11). To a solution of **7e** (50 mg, 0.09 mmol, 1.0 eq) in dioxane (1.0 mL) was added 2-oxa-6-azaspiro[3.3]heptane (90 mg, 0.91 mmol, 10.0 eq). The mixture was stirred at 60°C for 15 h. The mixture was concentrated under reduced pressure to give a residue. The residue was purified by Prep-TLC (DCM / MeOH = 15:1) to give **H11** as a white solid (13.8 mg, 26.7%). ^{1}H NMR (400 MHz, DMSO-d_6): δ 7.56-7.47 (m, 2H), 7.45-7.31 (m, 6H), 5.42 (s, 2H), 4.60 (s, 4H), 3.84 (t, J = 7.2 Hz, 2H), 3.53 (s, 4H), 3.29 (s, 3H), 2.64-2.51 (m, 2H), 1.60-1.43 (m, 2H), 1.35-1.13 (m, 2H). ^{13}C NMR (75 MHz, DMSO-d_6): δ 154.36, 154.16, 152.87, 150.99, 146.03, 135.71, 134.17, 133.11, 131.82, 130.02, 129.24, 126.45, 120.48, 119.06, 103.04, 79.98, 62.88, 46.38, 40.84, 38.69, 30.09, 25.53, 23.94. LC-MS: 570.1 ($[M+H]^+$). HPLC RT: 4.04 min, purity 99.6%.

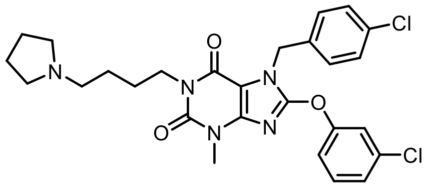

7-(4-chlorobenzyl)-8-(3-chlorophenoxy)-3-methyl-1-(4-(pyrrolidin-1-yl)butyl)-3,7-dihydro-1*H*-purine-2,6-dione (H12). To a solution of **7c** (50 mg, 0.09 mmol, 1.0 eq) in dioxane (1.0 mL) was added pyrrolidine (65 mg, 0.91 mmol, 10.0 eq). The

mixture was stirred at 60°C for 15 h. The mixture was concentrated under reduced pressure to give a residue. The residue was purified by Prep-TLC (DCM / MeOH = 15:1) to give **H12** (9.6 mg, 19.5%) as a colorless oil. ^{1}H NMR (400 MHz, DMSO-d_6): δ 7.57-7.45 (m, 2H), 7.44-7.26 (m, 6H), 5.42 (s, 2H), 3.87 (t, *J* = 7.2 Hz, 2H), 3.29 (s, 3H), 2.41-2.29 (m, 6H), 1.70-1.61 (m, 4H), 1.60-1.51 (m, 2H), 1.49-1.36 (m, 2H). ^{13}C NMR (75 MHz, DMSO-d_6): *δ* 154.38, 154.19, 152.84, 150.99, 146.00, 135.73, 134.17, 133.10, 131.82, 130.04, 129.22, 126.43, 120.48, 119.06, 103.05, 55.86, 54.04, 45.58, 40.84, 30.08, 26.33, 26.15, 23.55. LC-MS: 542.2 ([M+H]$^+$). HPLC RT: 4.09 min, purity 97.0%.

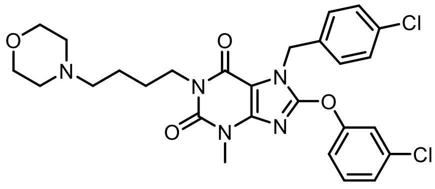

7-(4-chlorobenzyl)-8-(3-chlorophenoxy)-3-methyl-1-(4-morpholinobutyl)-3,7-dihydro-1*H*-purine-2,6-dione (H13). To a solution of **7c** (50 mg, 0.09 mmol, 1.0 eq) in dioxane (1.0 mL) was added morpholine (79 mg, 0.91 mmol, 10.0 eq). The mixture was stirred at 60°C for 15 h. The mixture was concentrated under reduced pressure to give a residue. The residue was purified by Prep-TLC (DCM / MeOH = 15:1) to give **H13** as a white solid (13.2 mg, 26.1%). ^{1}H NMR (400 MHz, DMSO-d_6): *δ* 7.54-7.47 (m, 2H), 7.46-7.30 (m, 6H), 5.42 (s, 2H), 3.88 (t, *J* = 7.2 Hz, 2H), 3.64-3.45 (m, 4H), 3.29 (s, 3H), 2.44-2.04 (m, 6H), 1.62-1.50 (m, 2H), 1.49-1.33 (m, 2H). ^{13}C NMR (75 MHz, DMSO-d_6): *δ* 154.37, 154.18, 152.83, 150.99, 146.01, 135.72, 134.17, 133.10, 131.81, 130.04, 129.21, 126.43, 120.47, 119.05, 103.05, 66.60, 58.37, 53.74, 46.37, 40.78, 30.07, 25.96, 23.77. LC-MS: 558.1 ([M+H]$^+$). HPLC RT: 3.97 min, purity 99.5%.

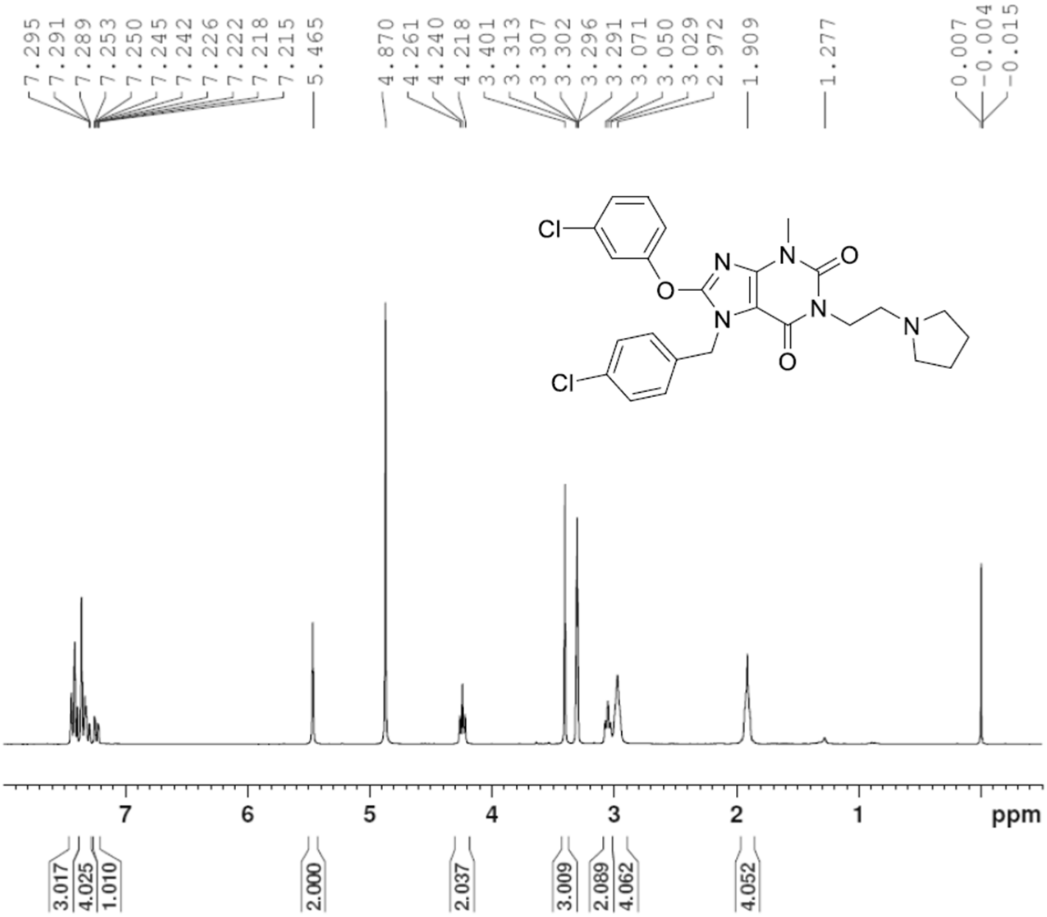

^{1}H NMR of compound **H01**

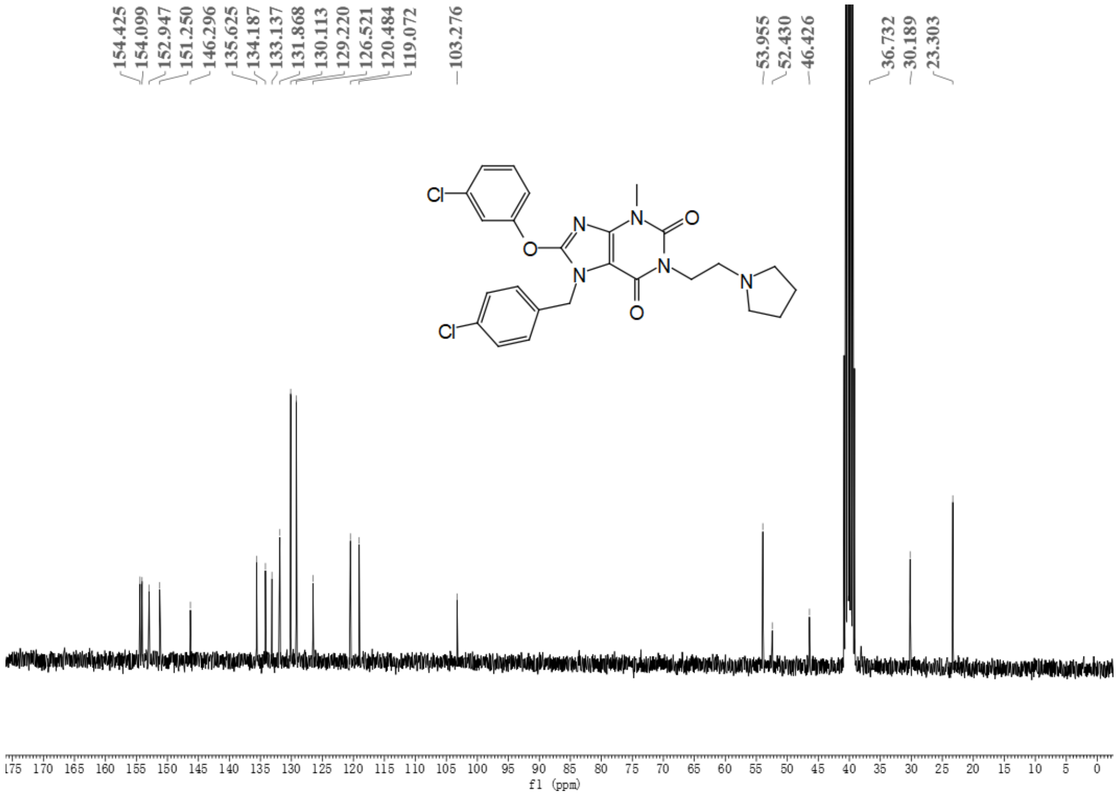

^{13}C NMR of compound **H01**

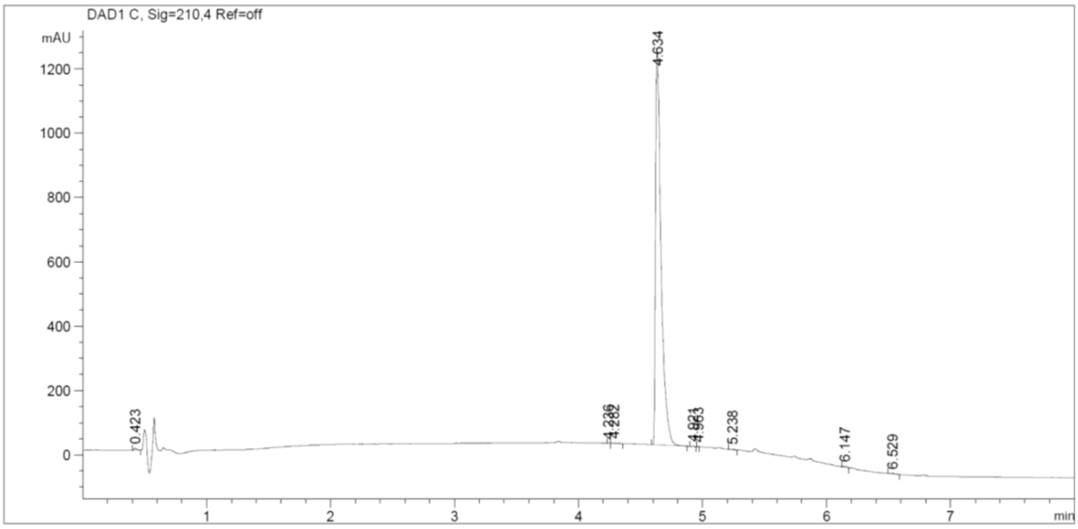

HPLC of compound **H01**

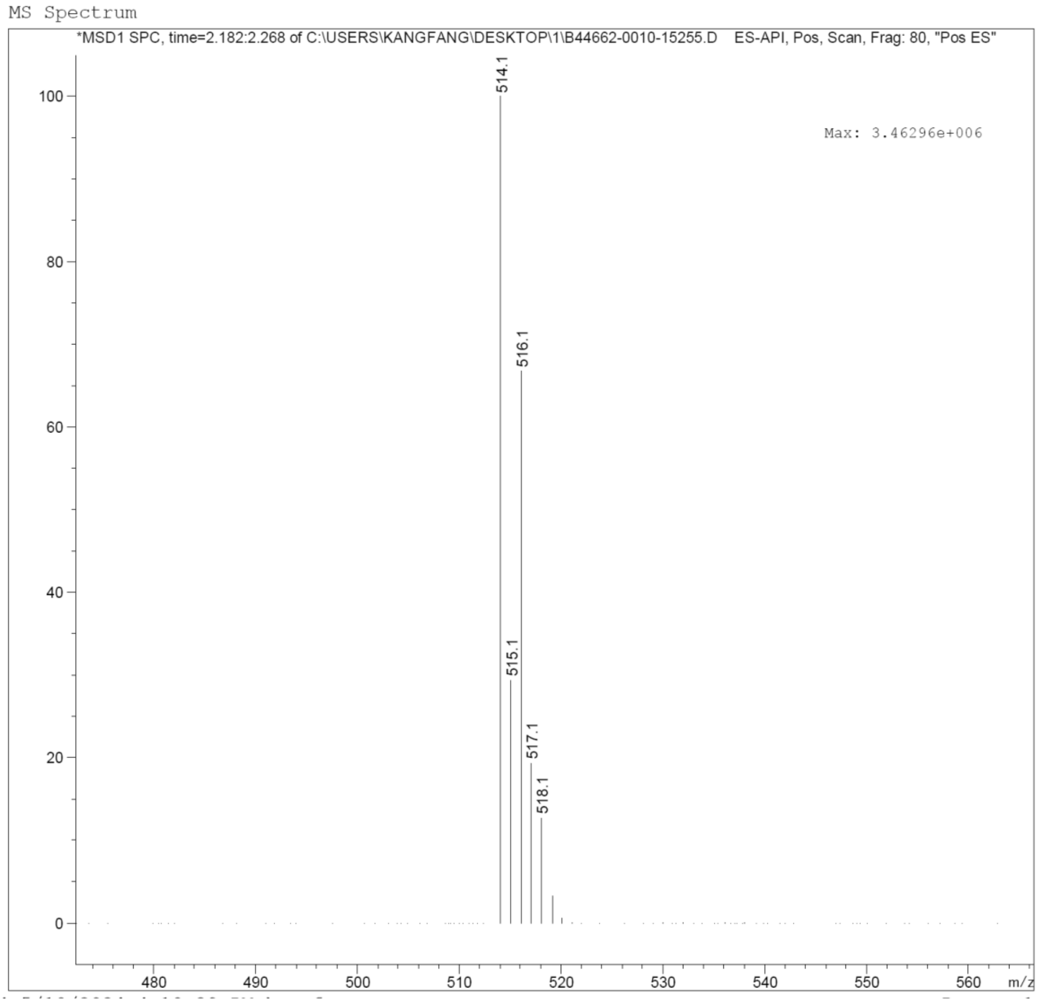

LC-MS of compound **H01**

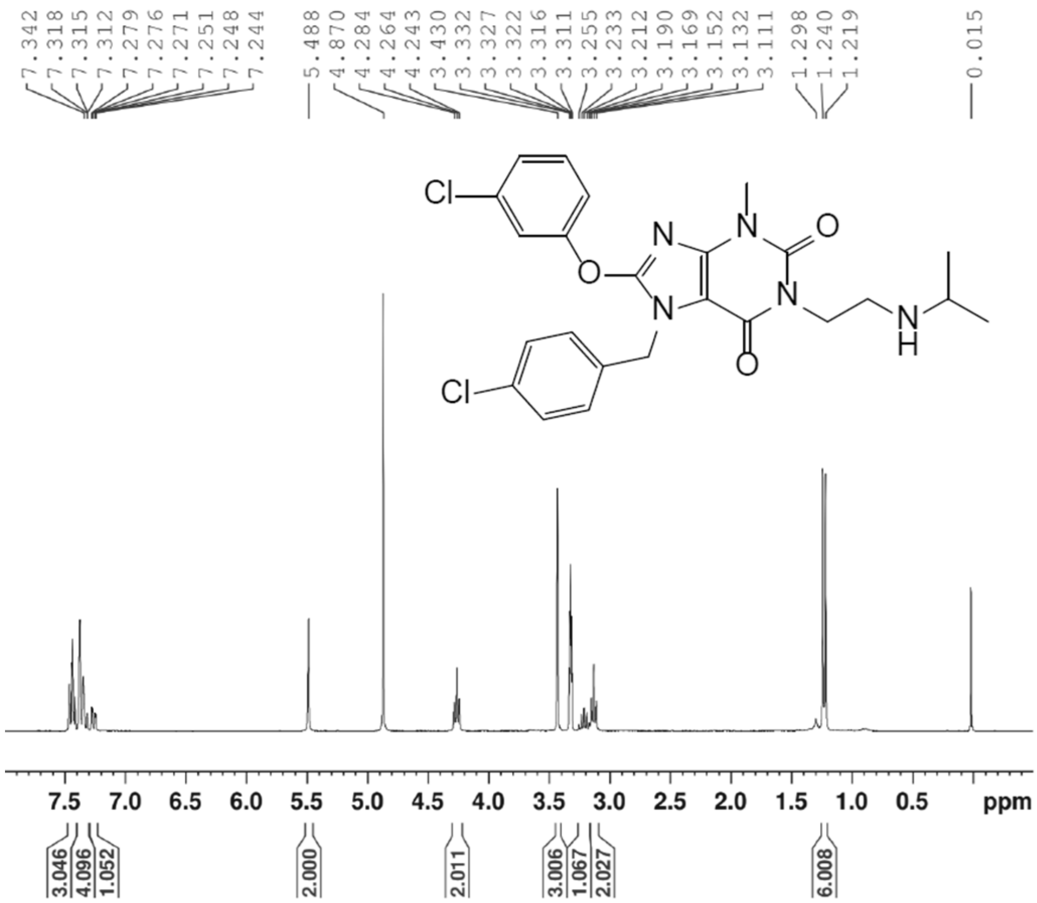

^{1}H NMR of compound **H02**

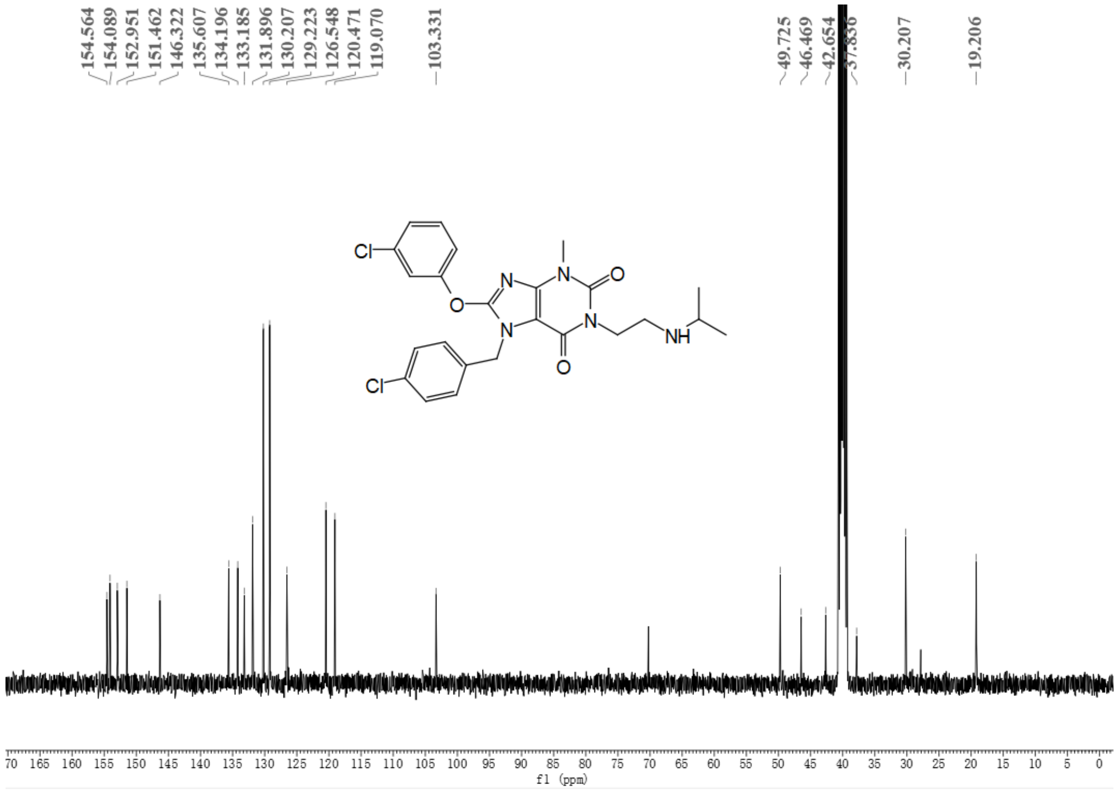

^{13}C NMR of compound **H02**

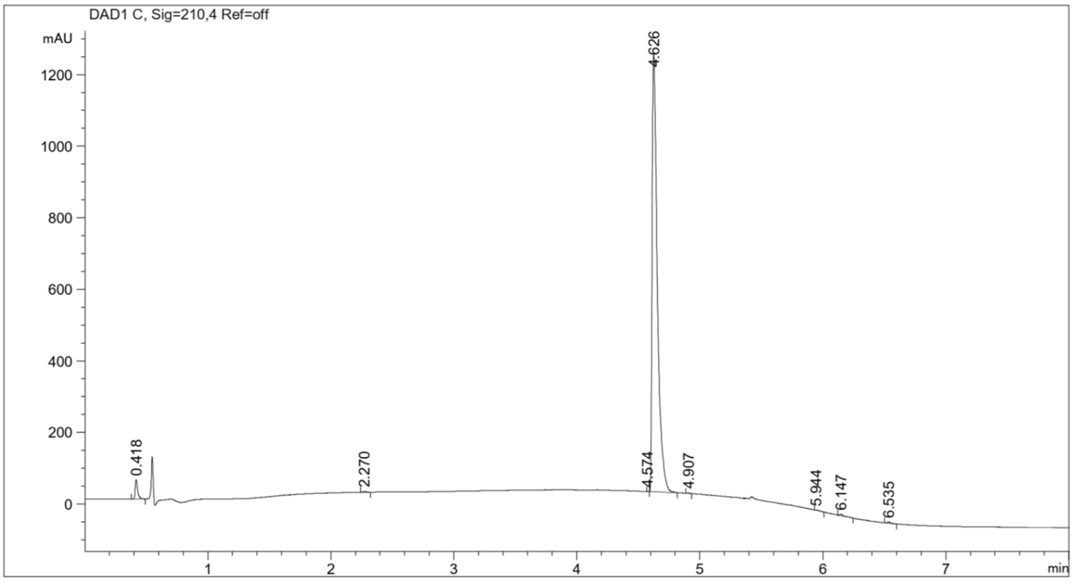

HPLC of compound **H02**

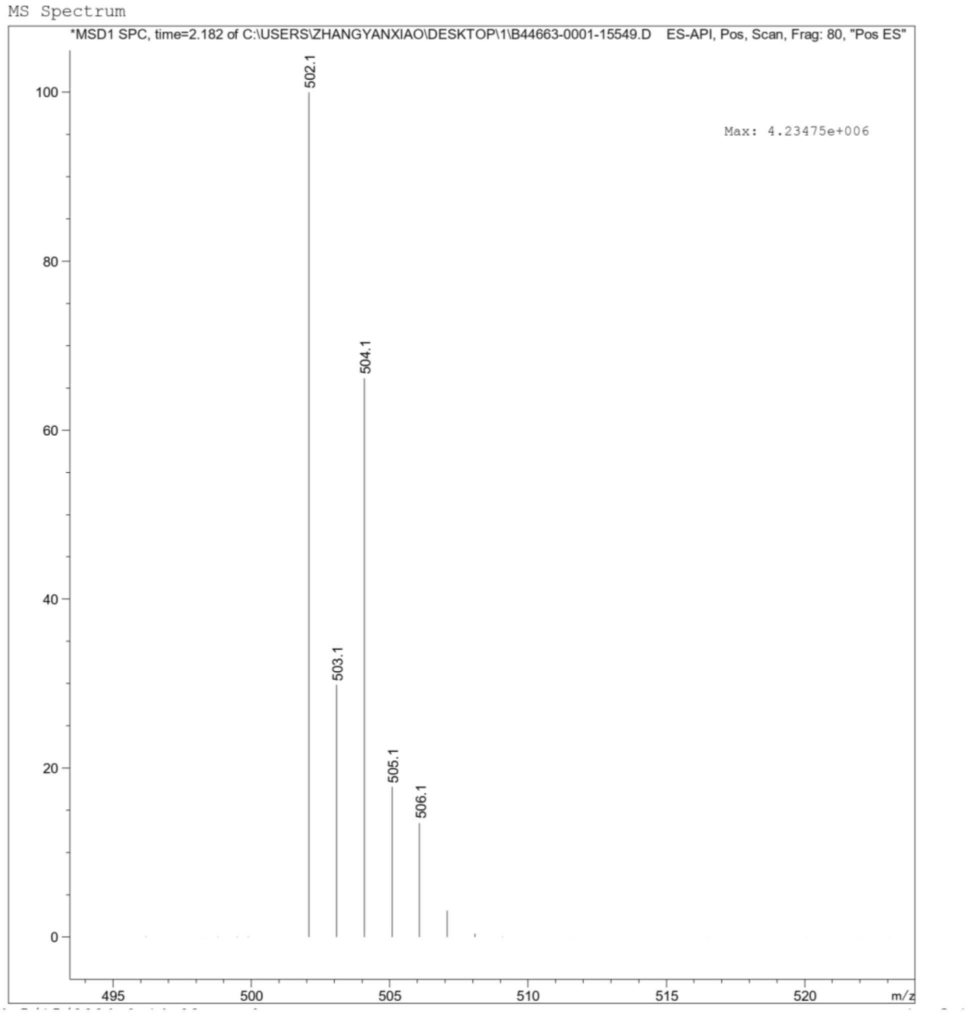

LC-MS of compound **H02**

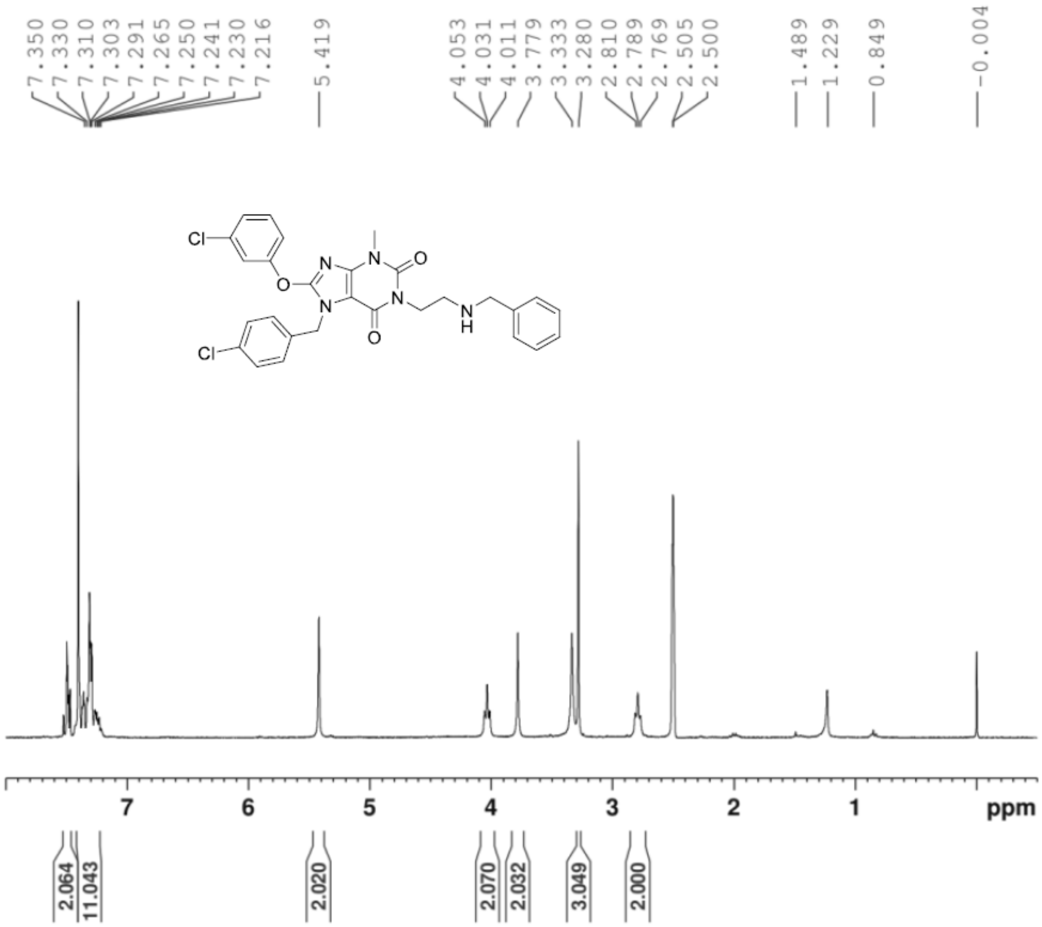

^{1}H NMR of compound **H03**

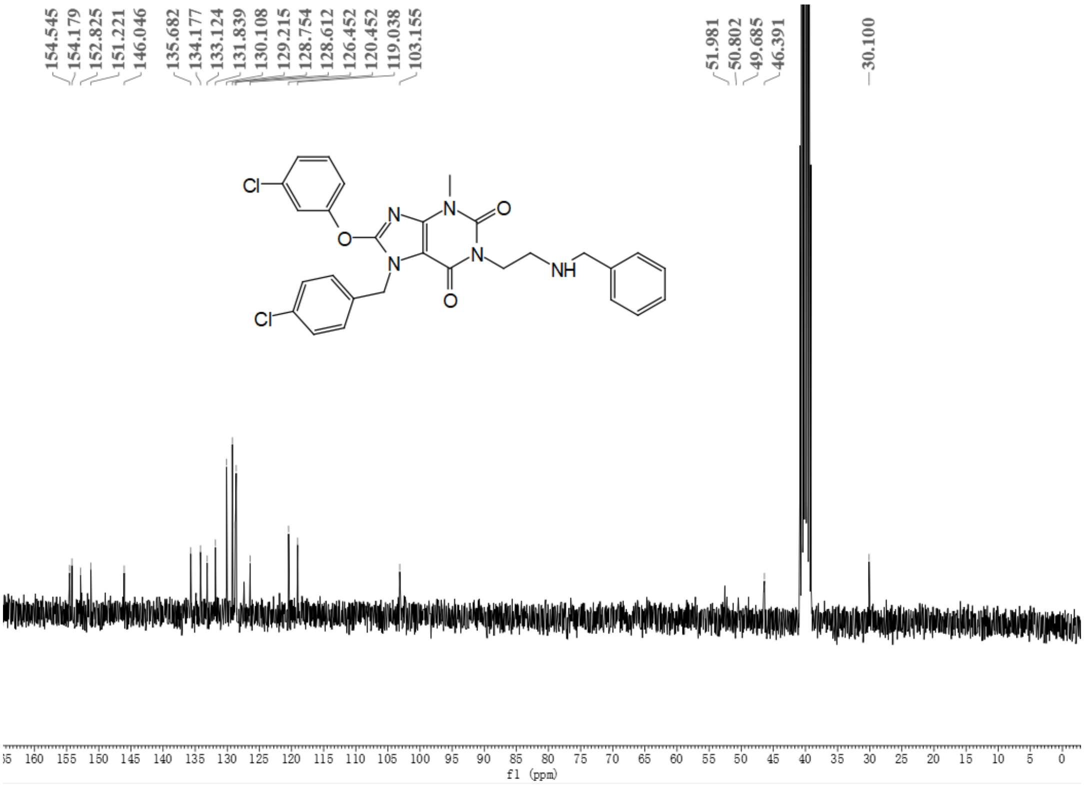

^{13}C NMR of compound **H03**

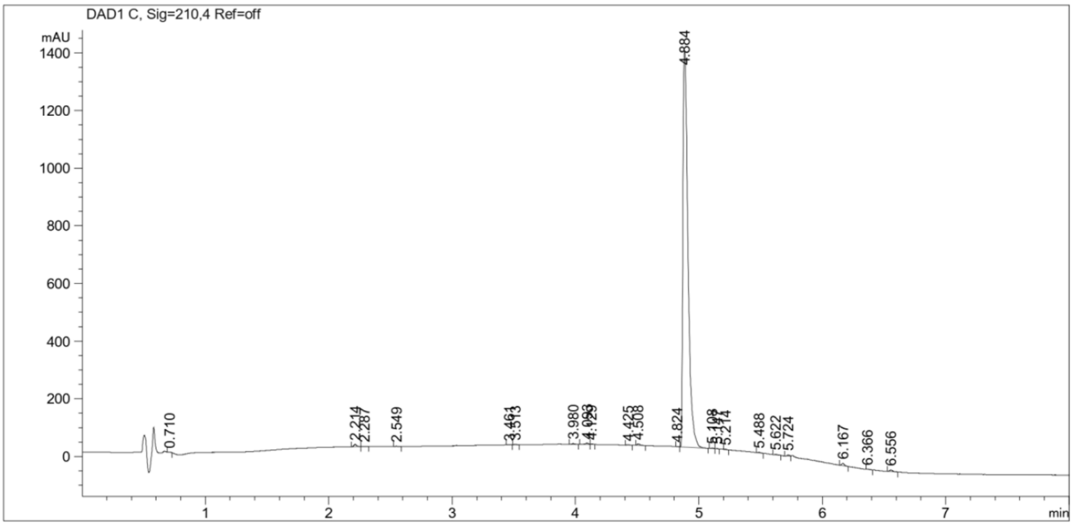

HPLC of compound **H03**

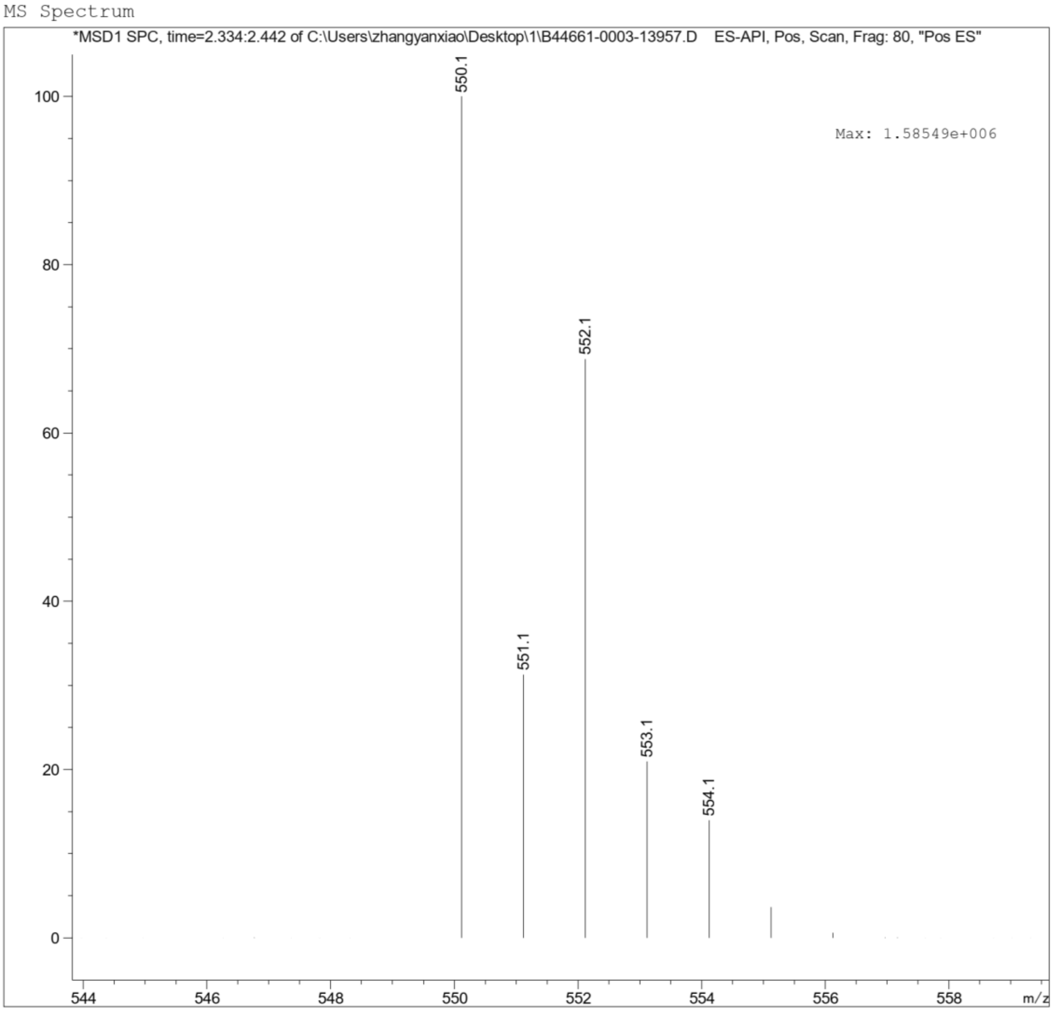

LC-MS of compound **H03**

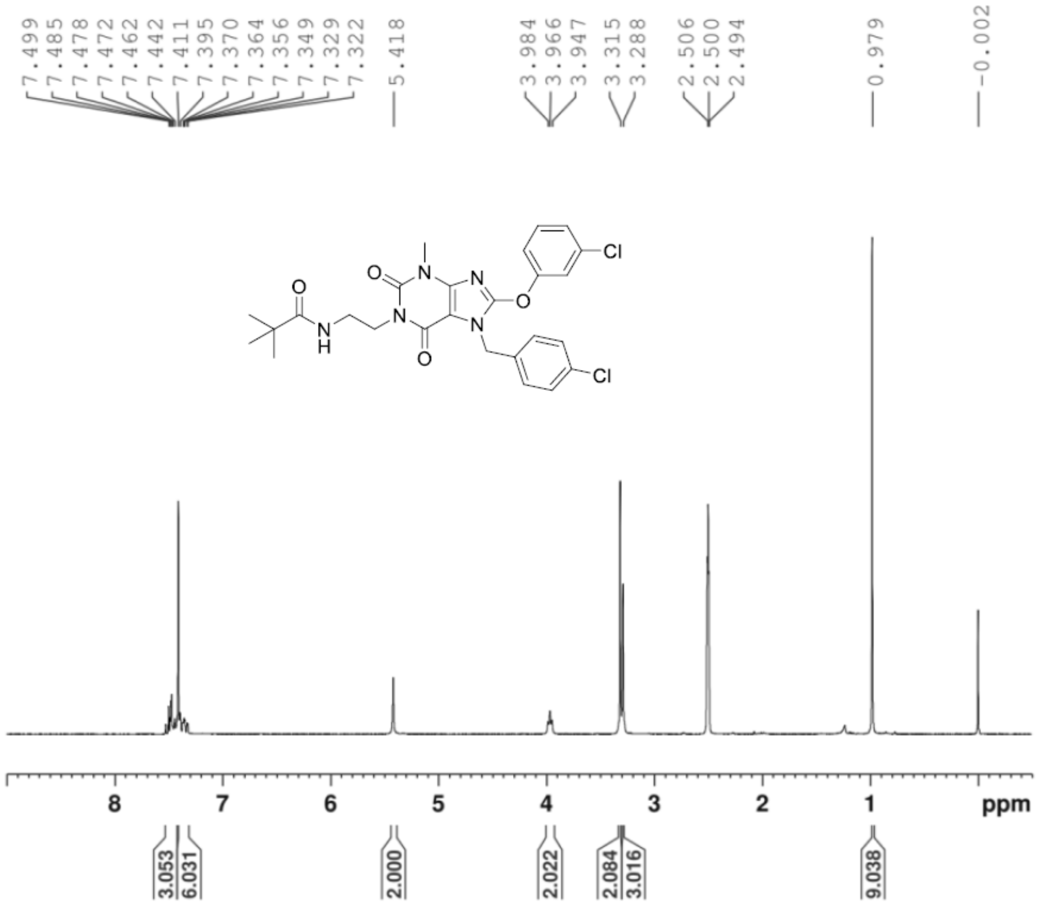

^{1}H NMR of compound **H04**

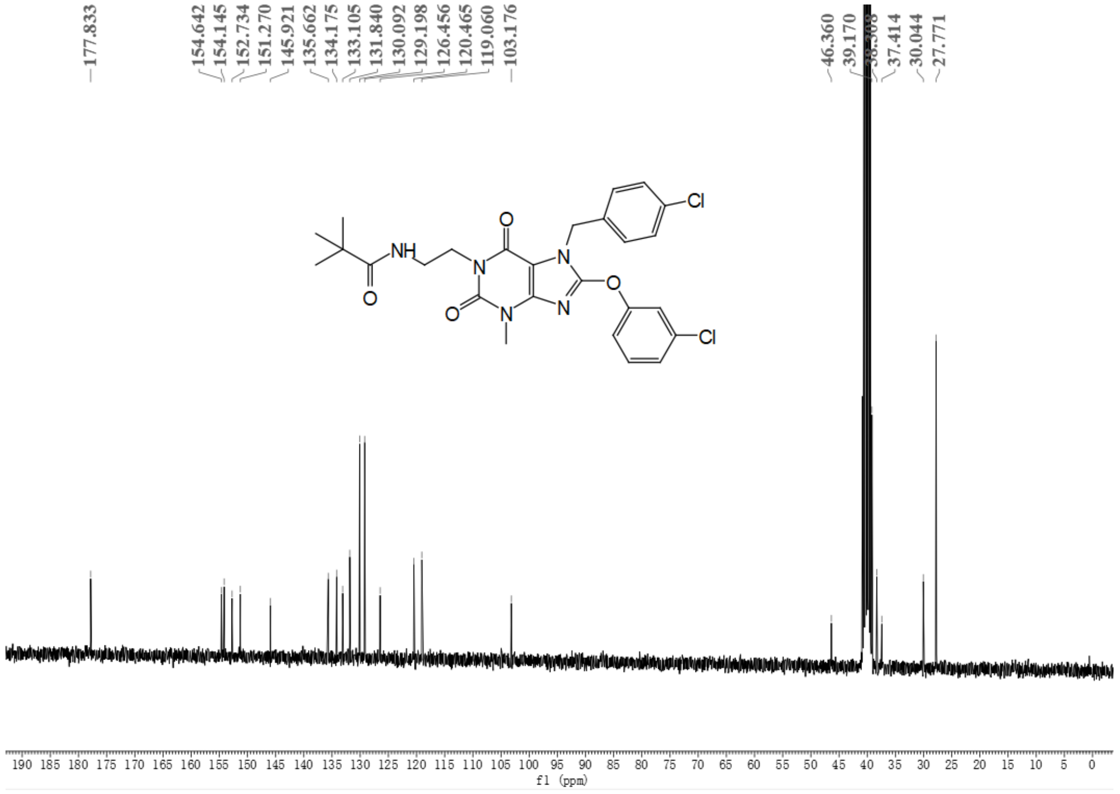

^{13}C NMR of compound **H04**

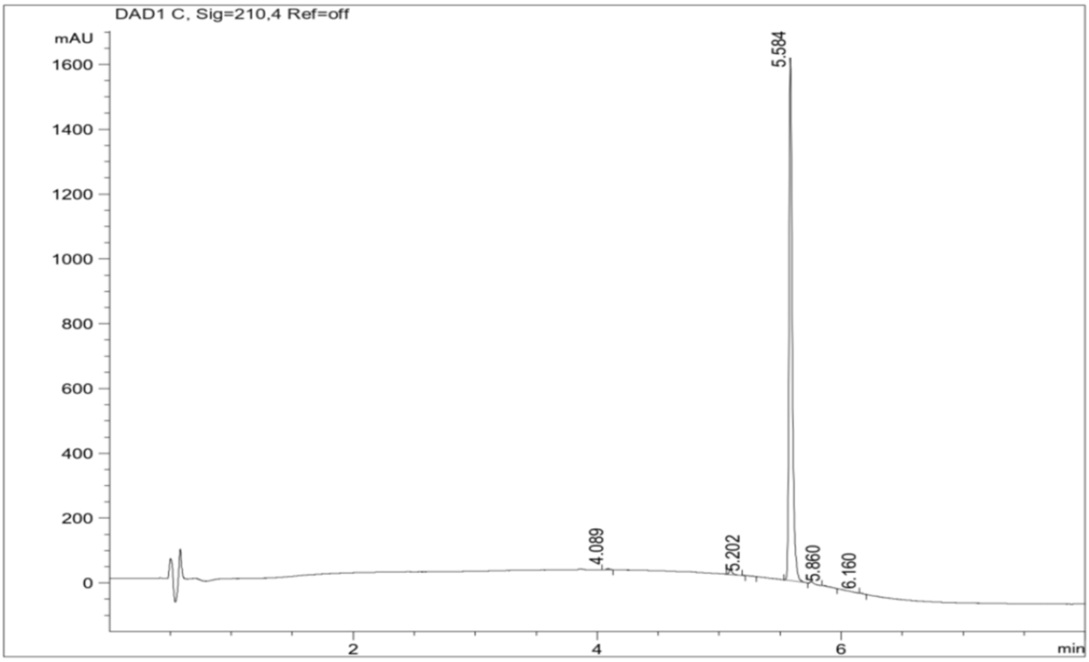

HPLC of compound **H04**

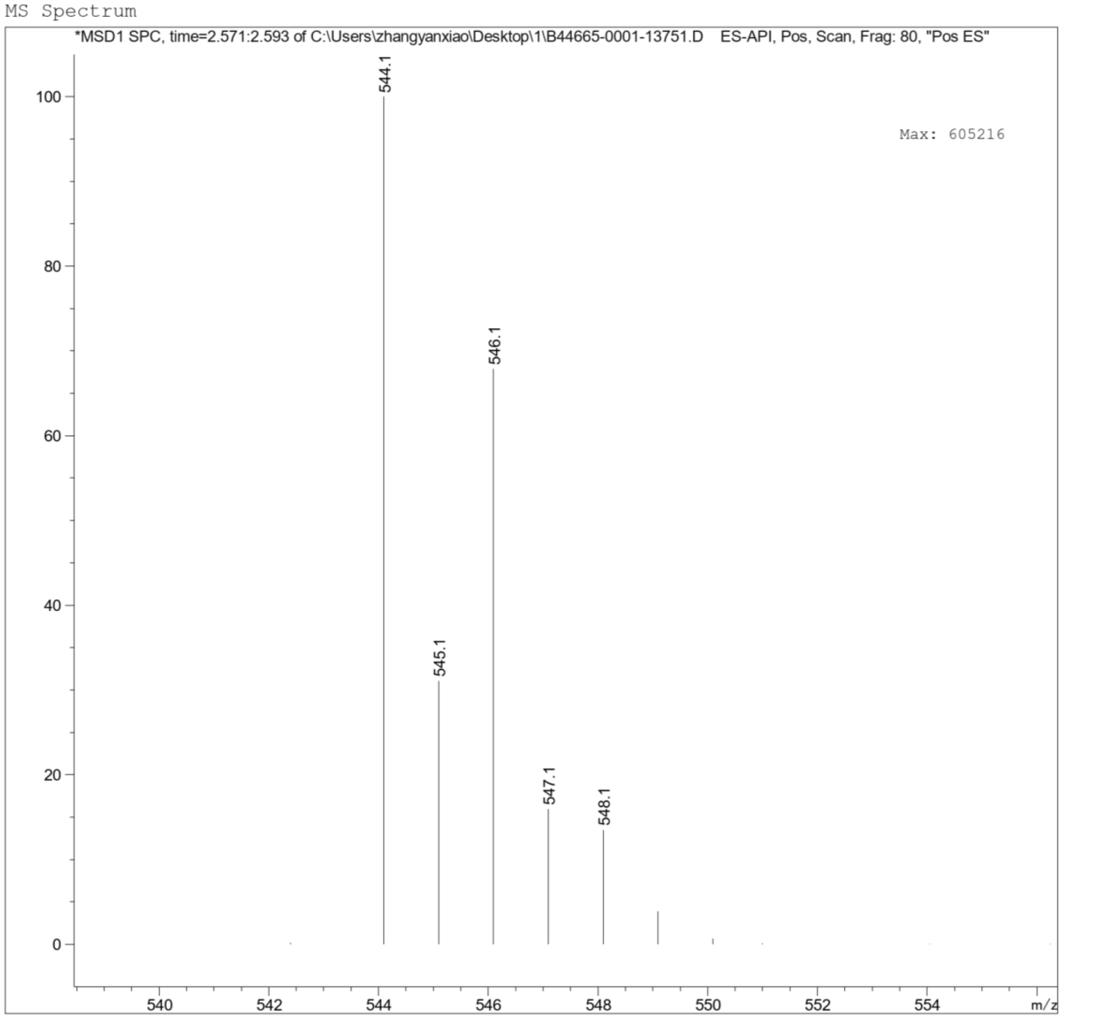

LC-MS of compound **H04**

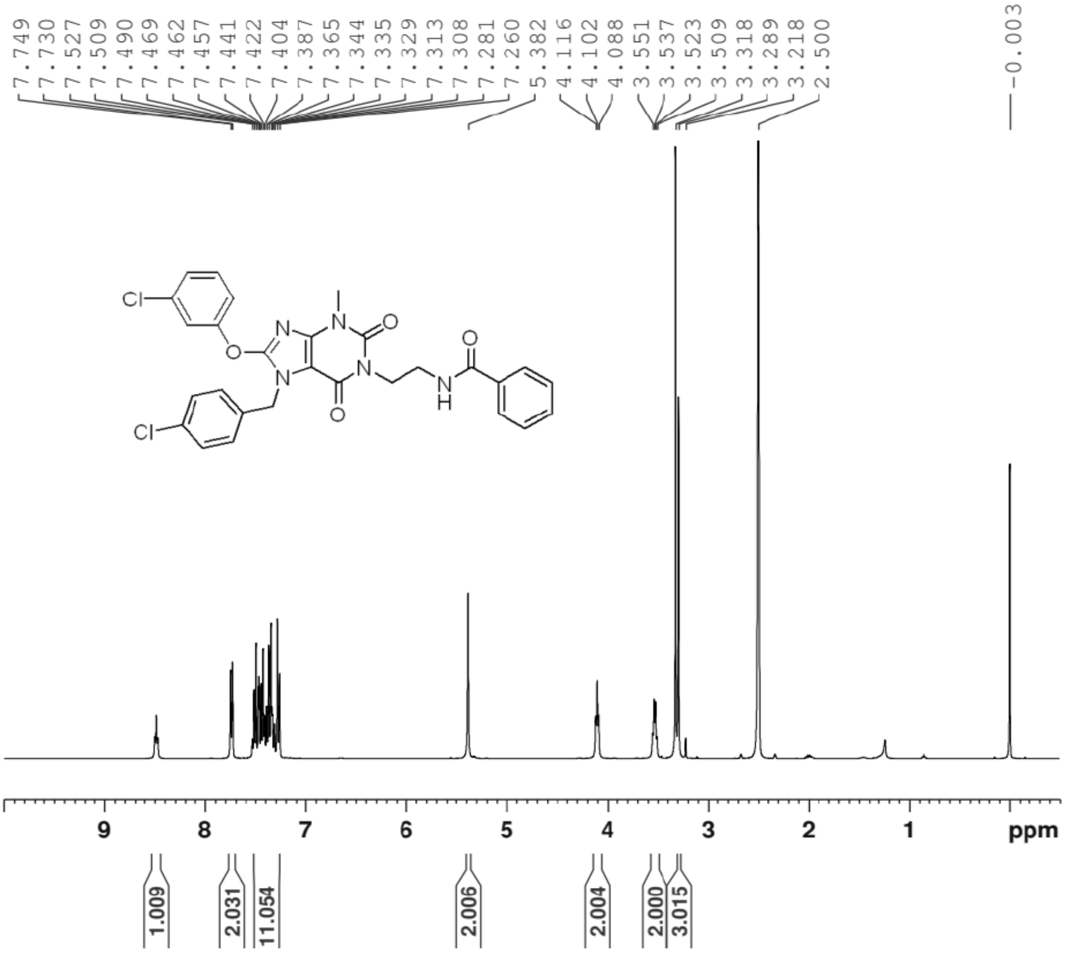

^{1}H NMR of compound **H05**

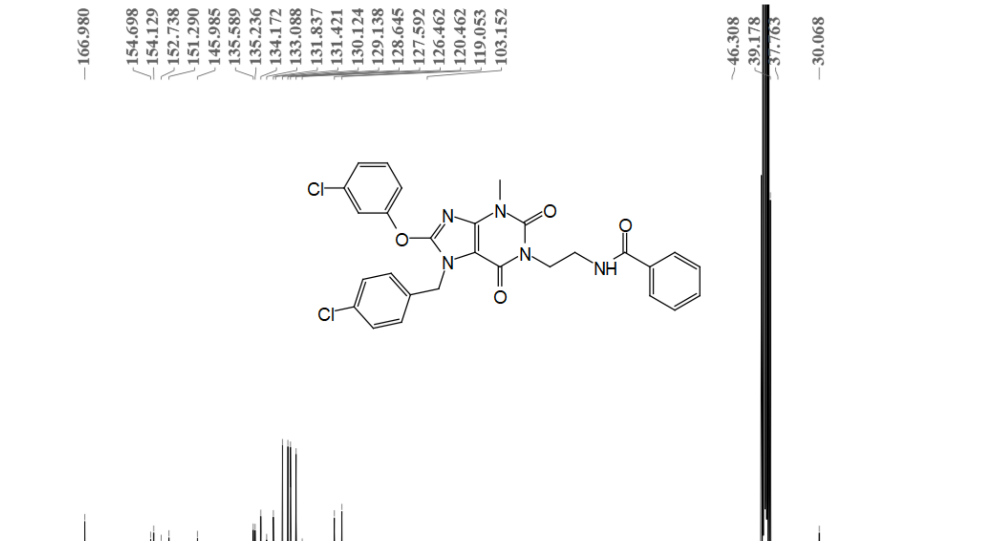

^{13}C NMR of compound **H05**

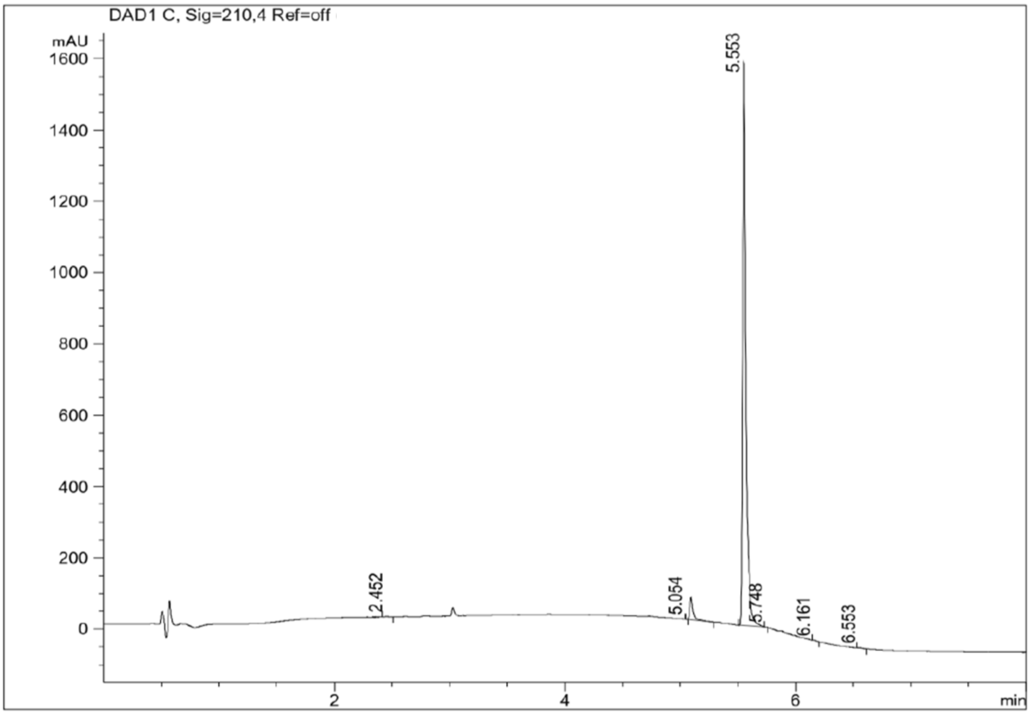

HPLC of compound **H05**

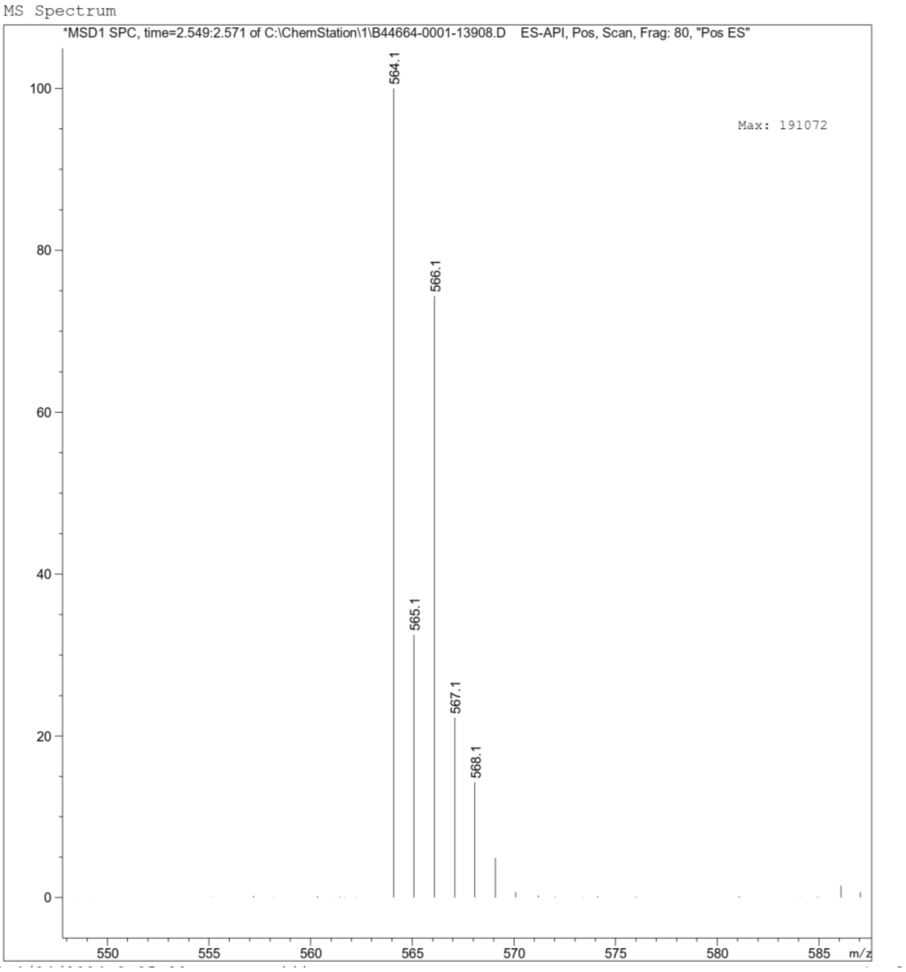

LC-MS of compound **H05**

482

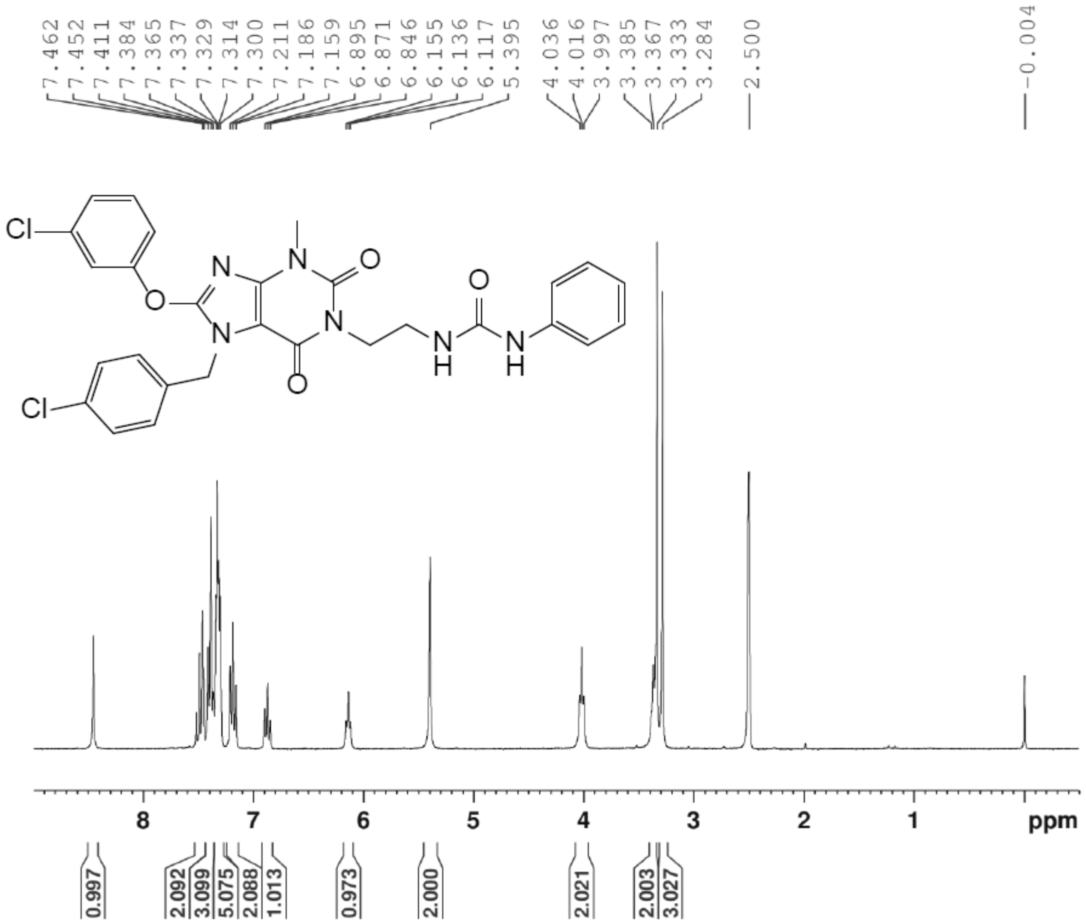

483 ^{1}H NMR of compound **H06**

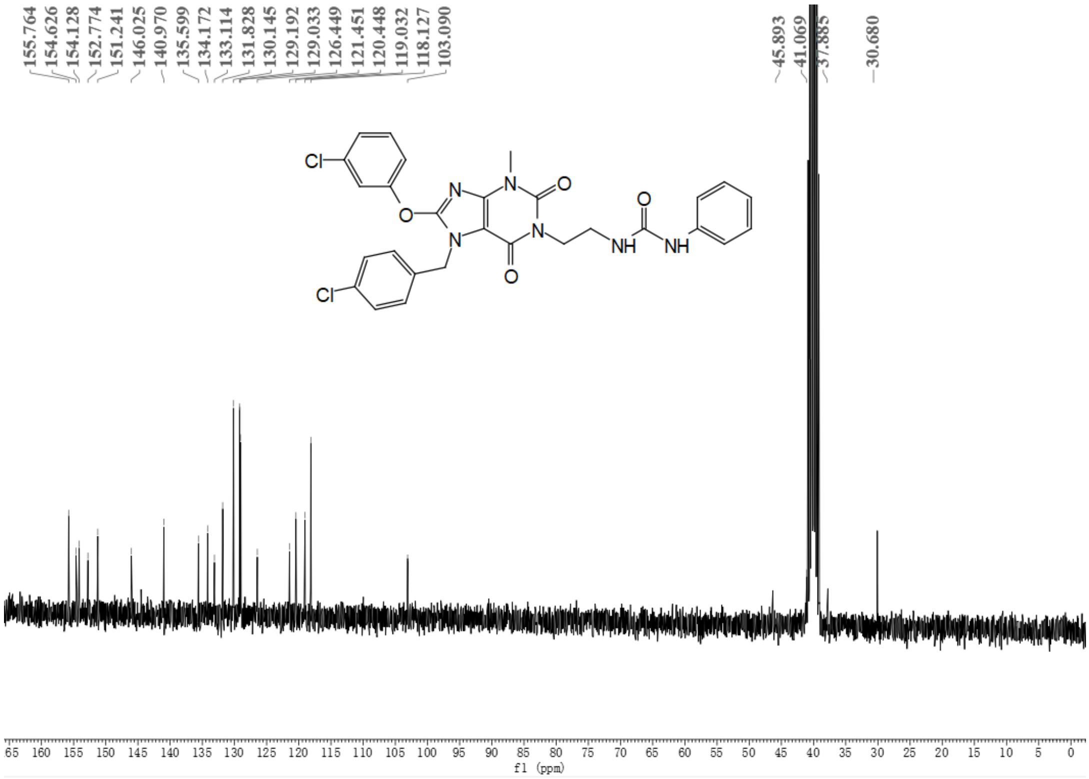

484

485 ^{13}C NMR of compound **H06**

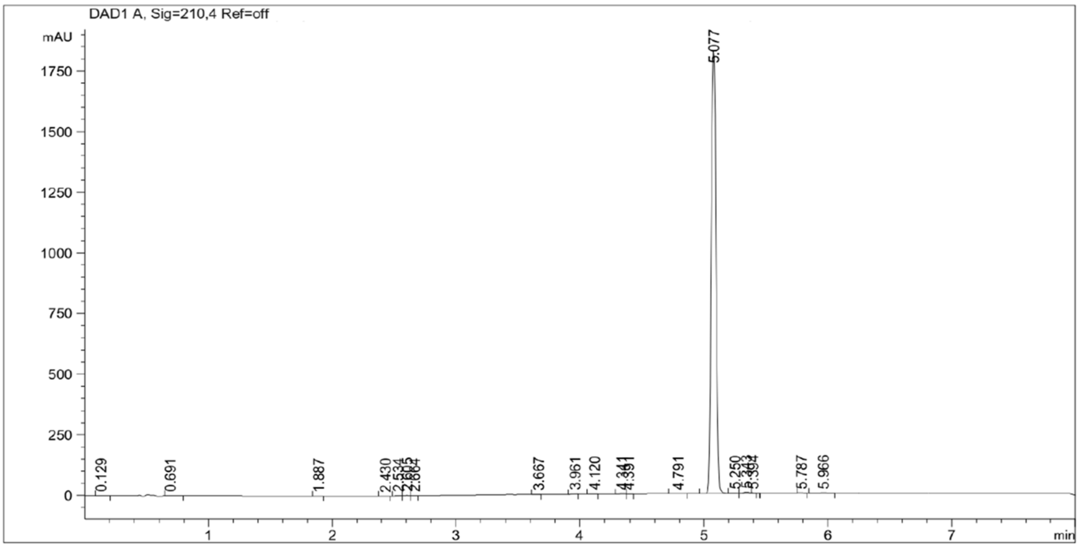

HPLC of compound **H06**

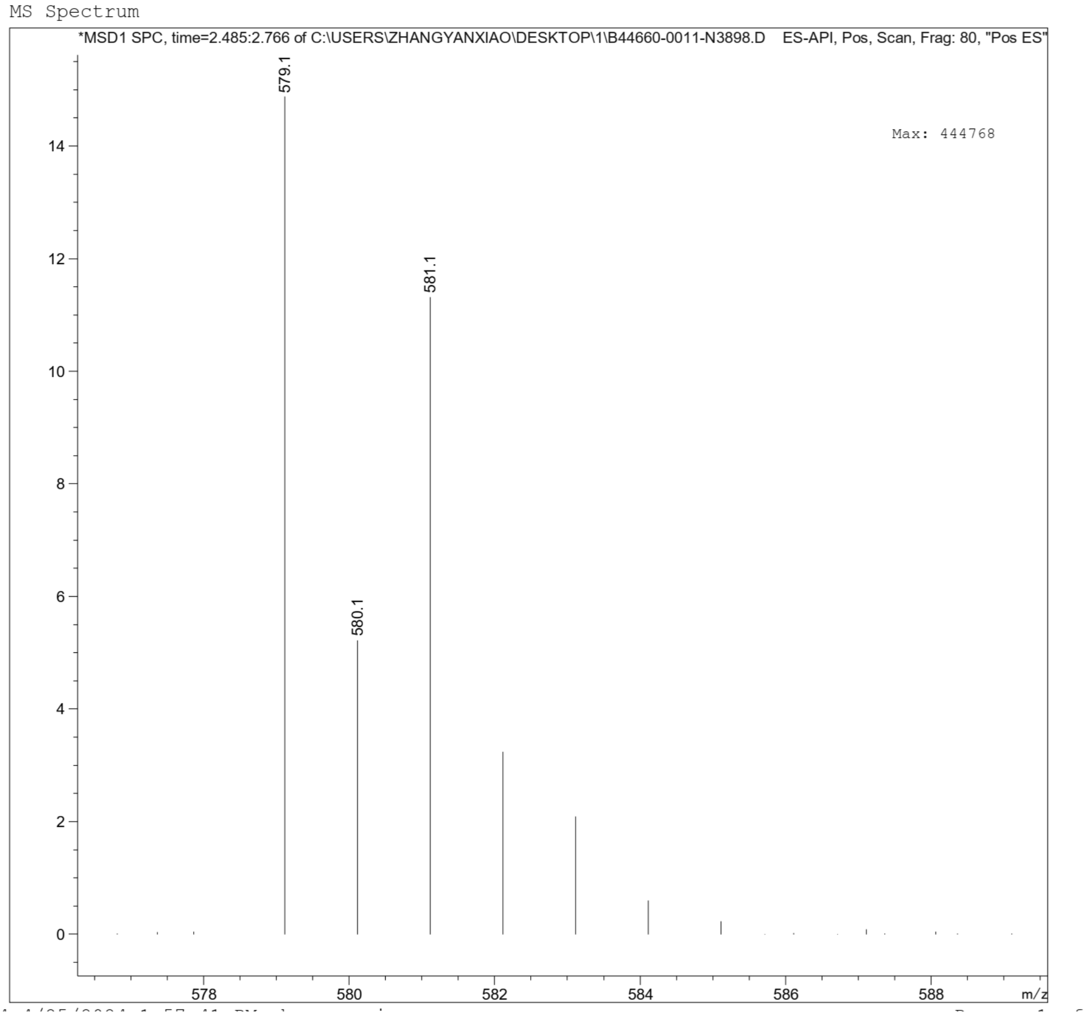

LC-MS of compound **H06**

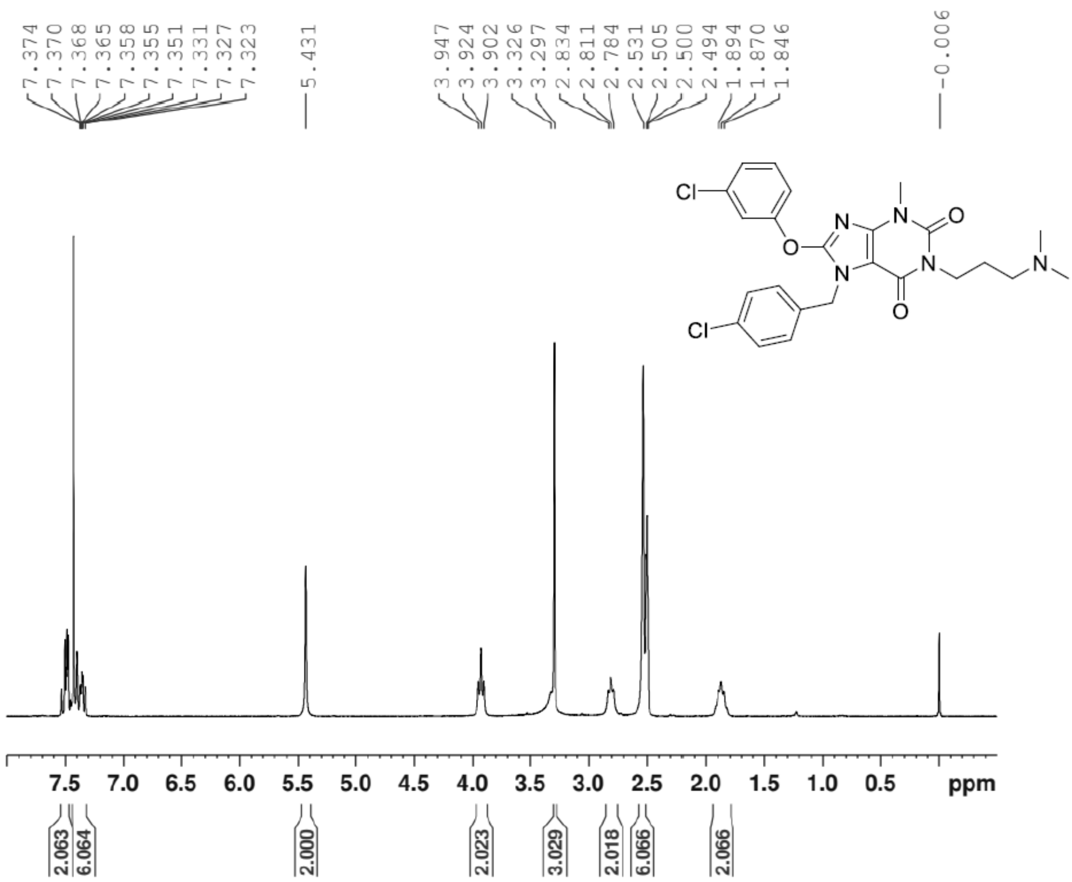

^{1}H NMR of compound **H07**

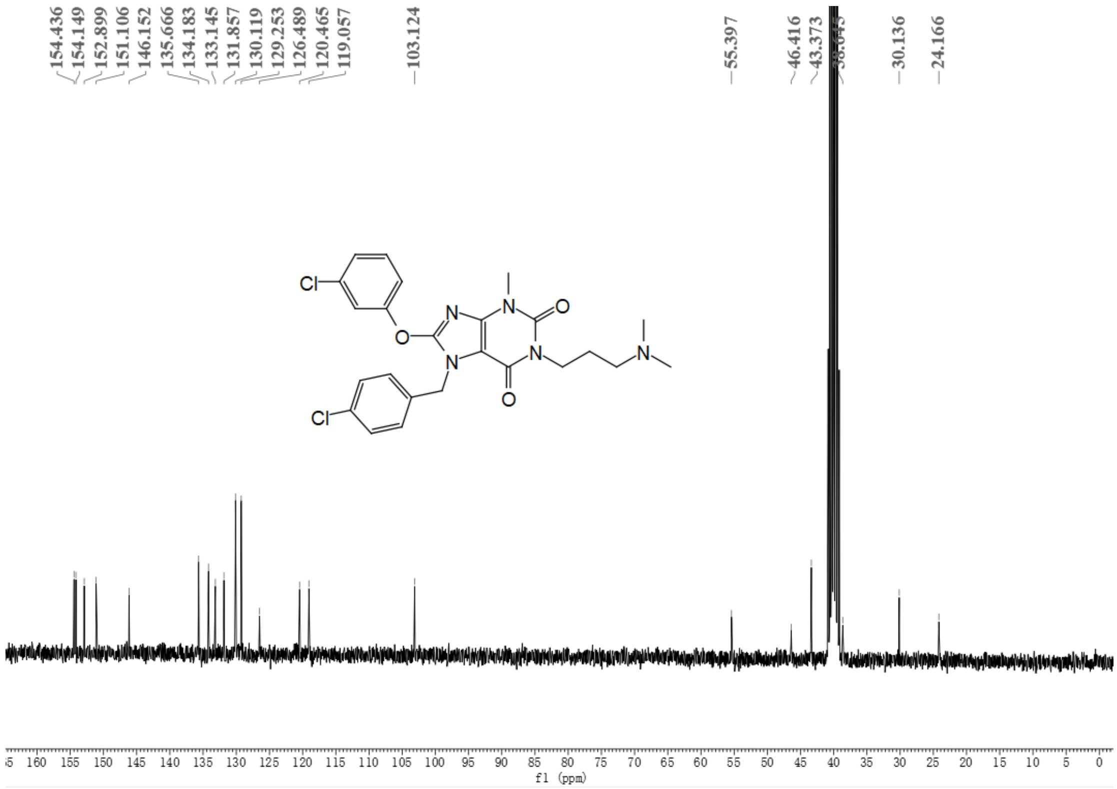

^{13}C NMR of compound **H07**

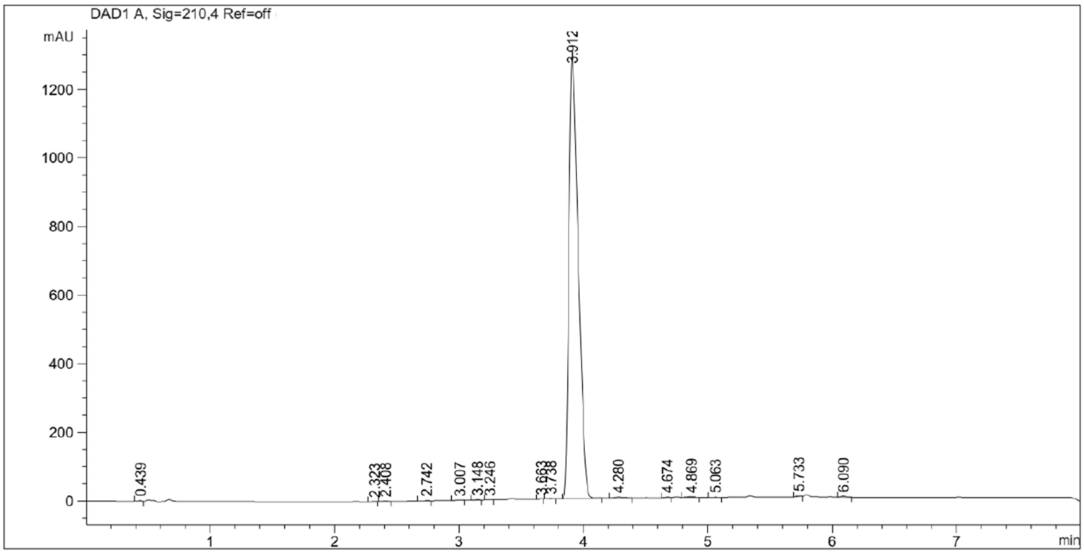

HPLC of compound **H07**

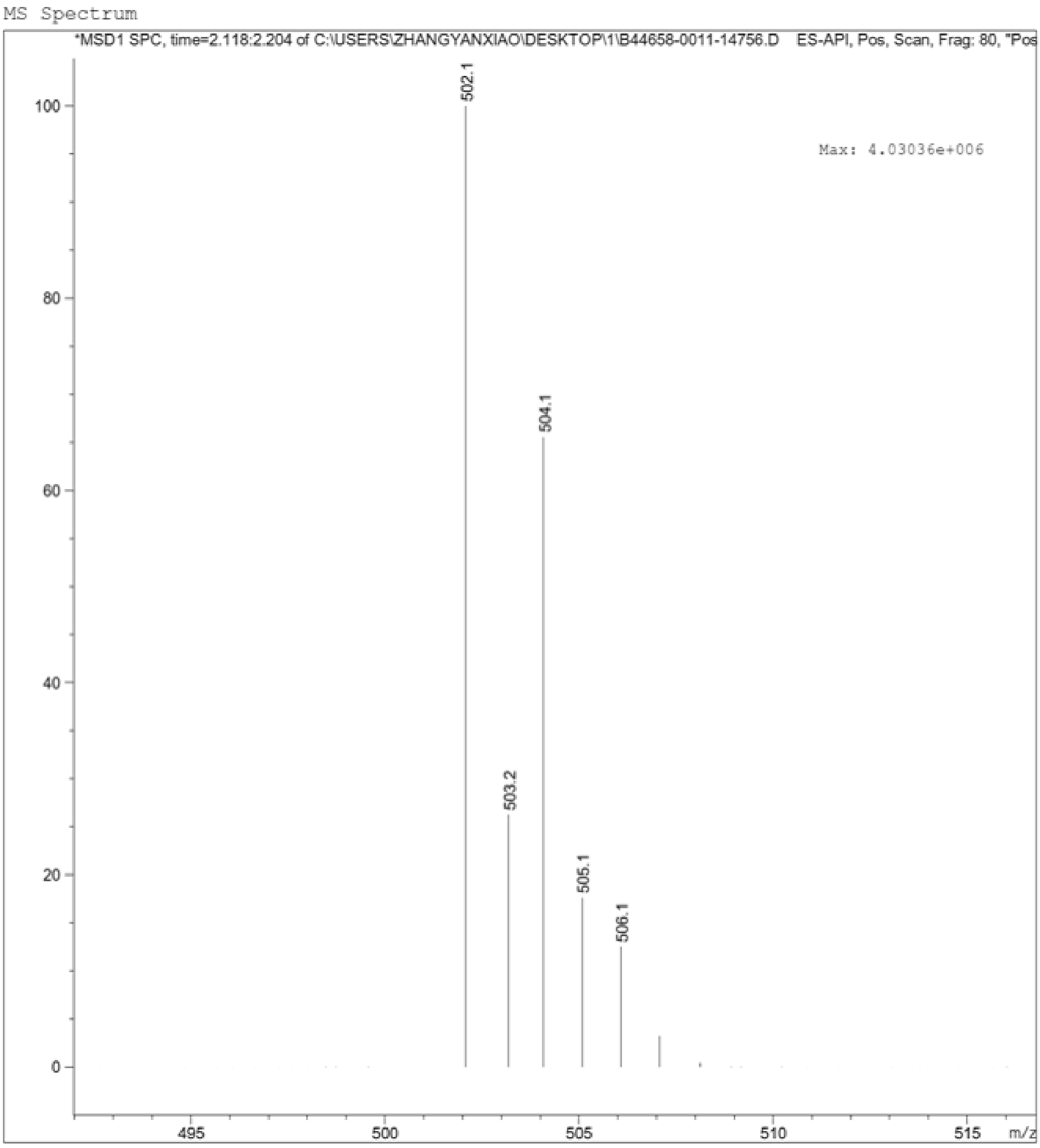

LC-MS of compound H07

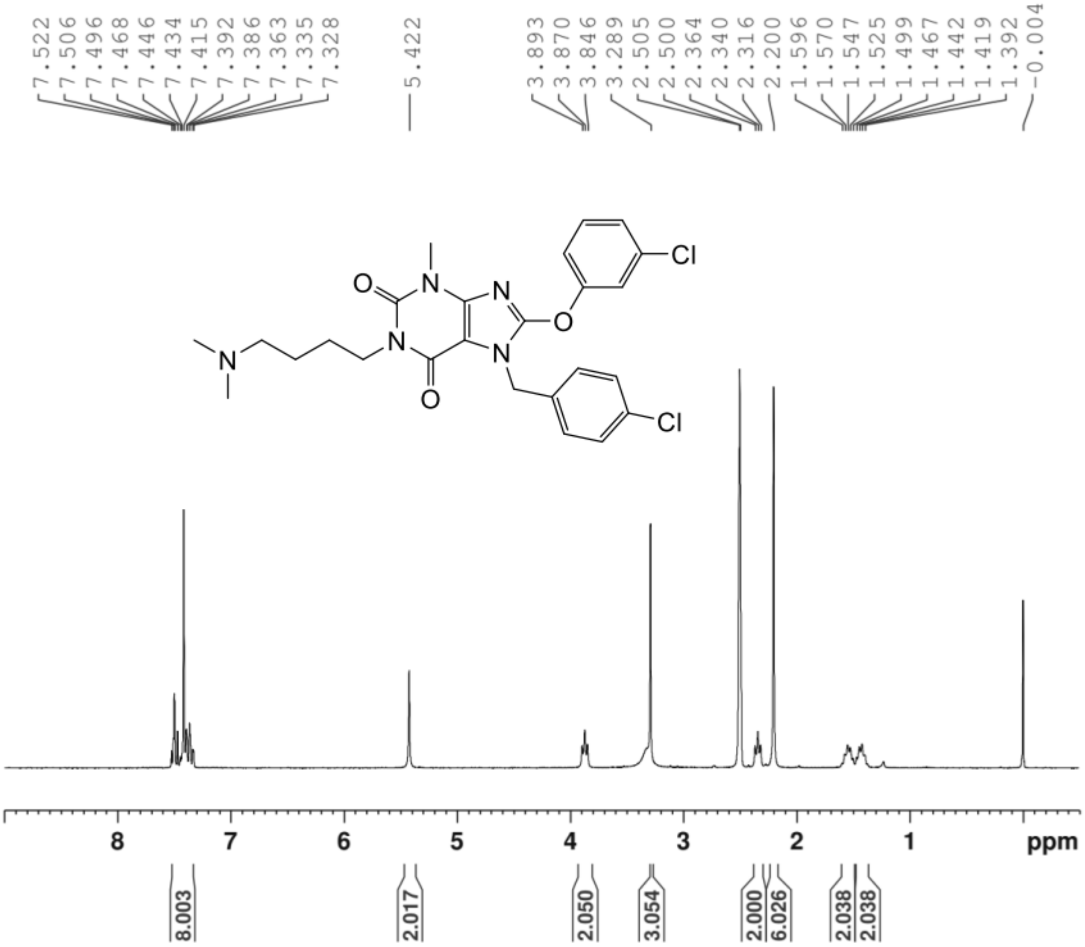

^{1}H NMR of compound **H08**

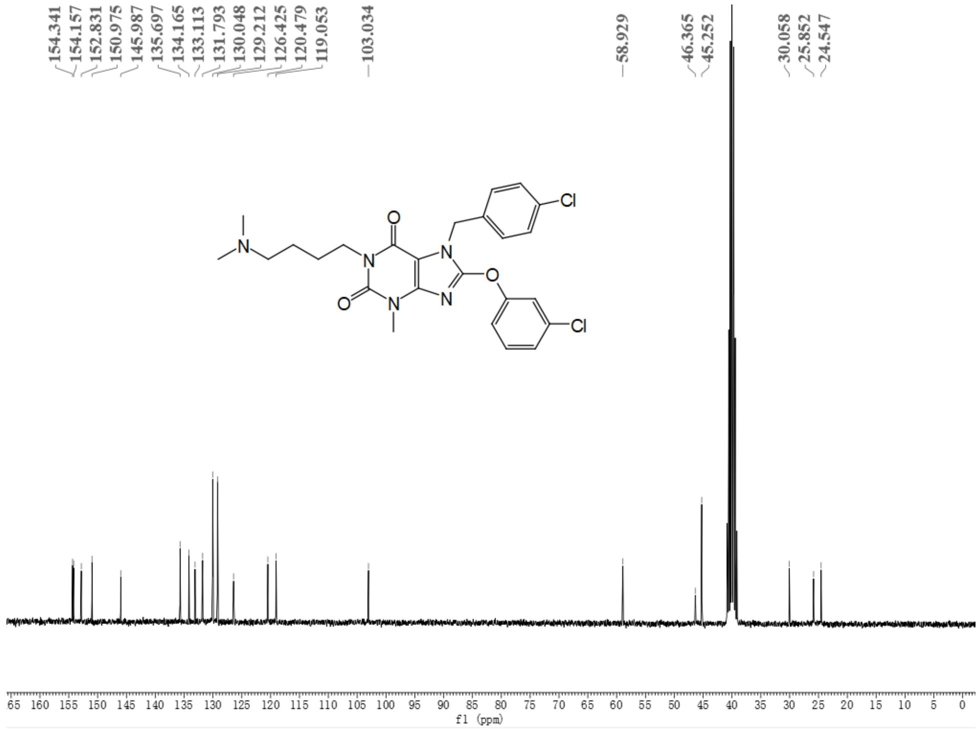

^{13}C NMR of compound **H08**

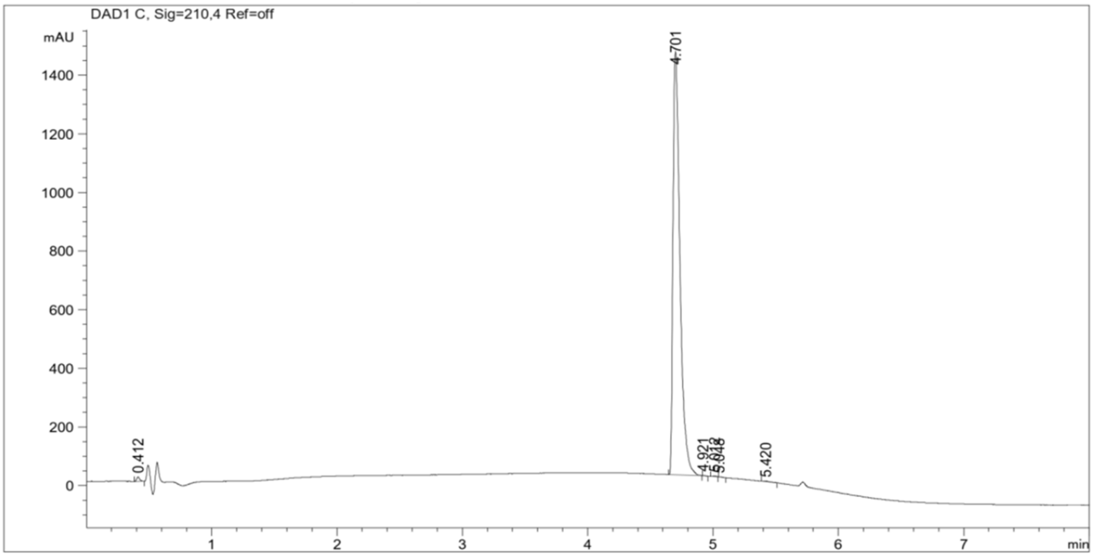

HPLC of compound **H08**

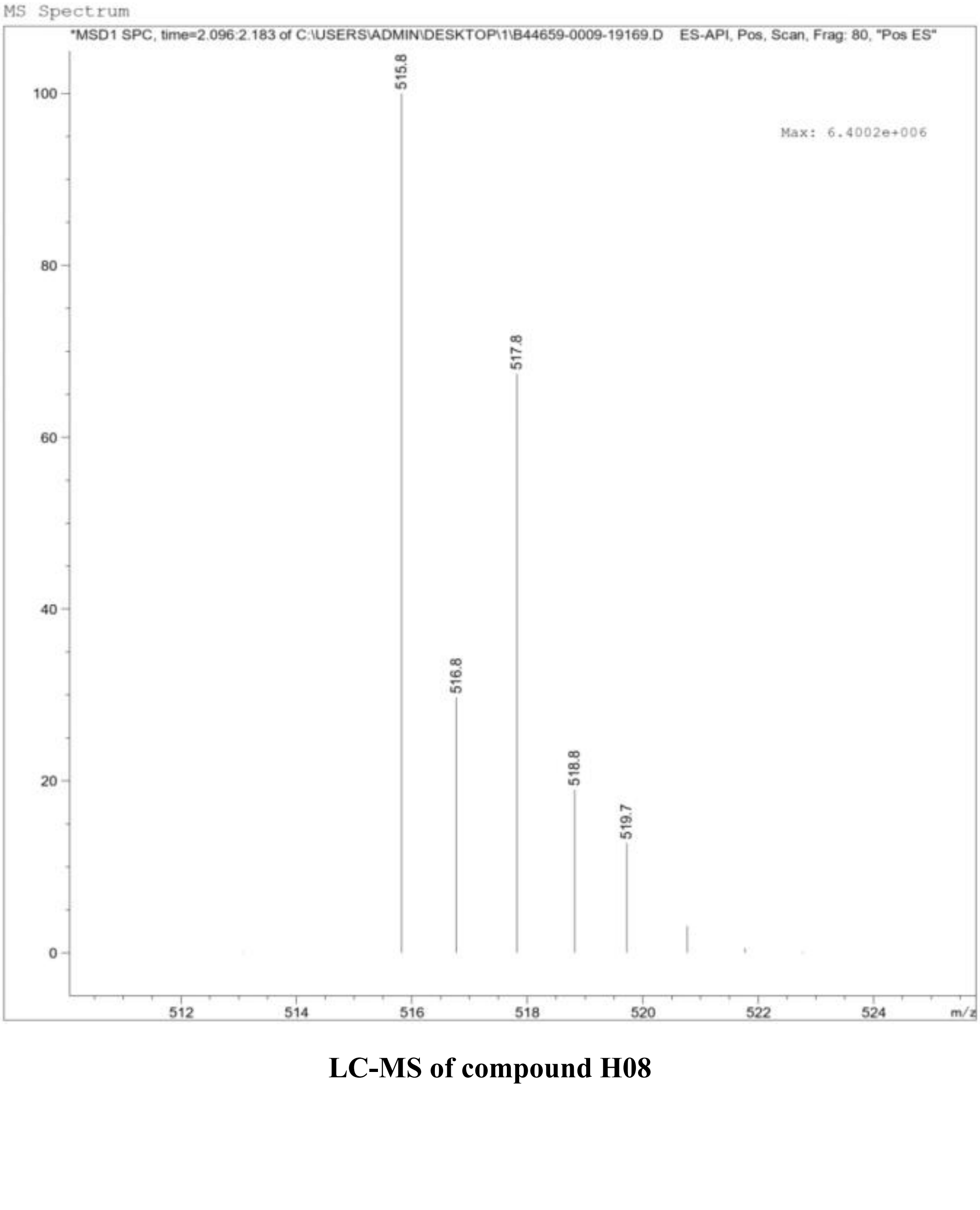

LC-MS of compound H08

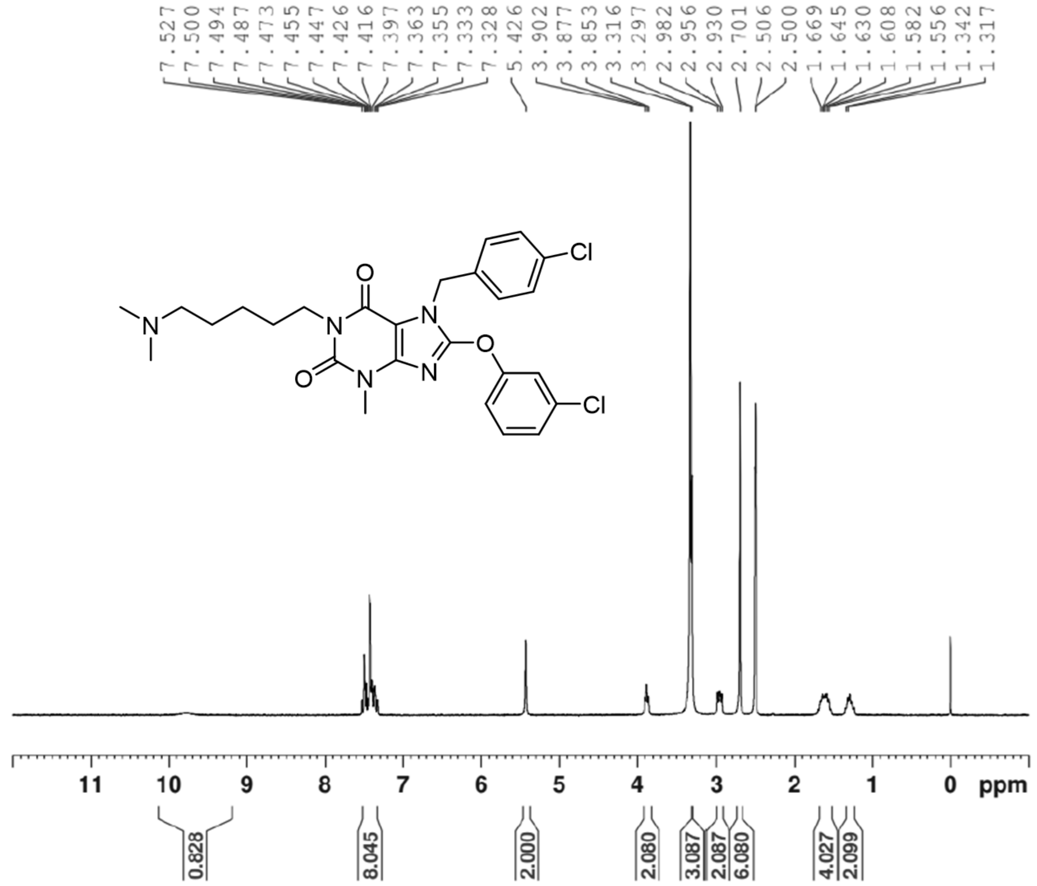

^{1}H NMR of compound **H09**

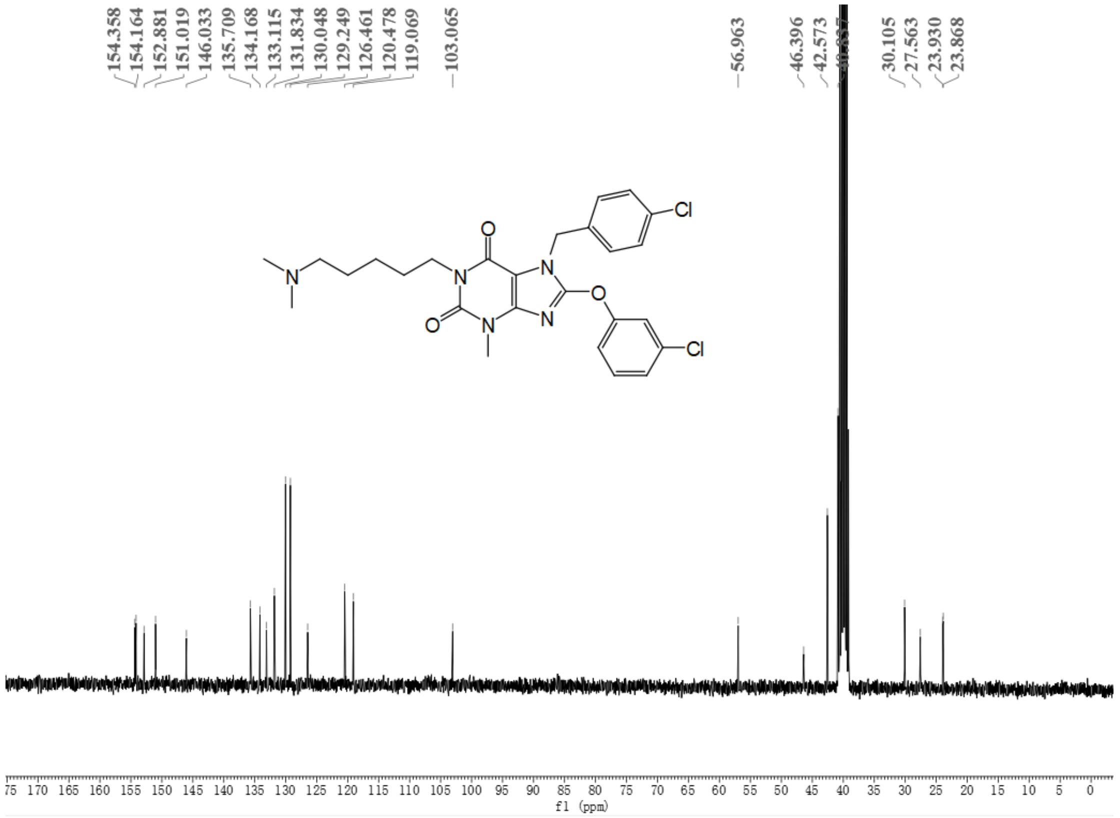

^{13}C NMR of compound **H09**

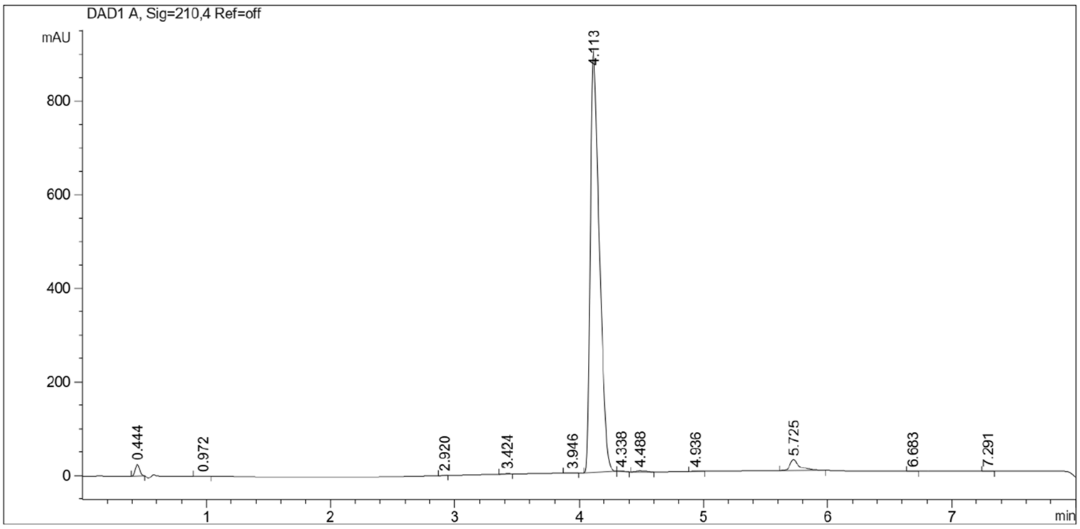

HPLC of compound **H09**

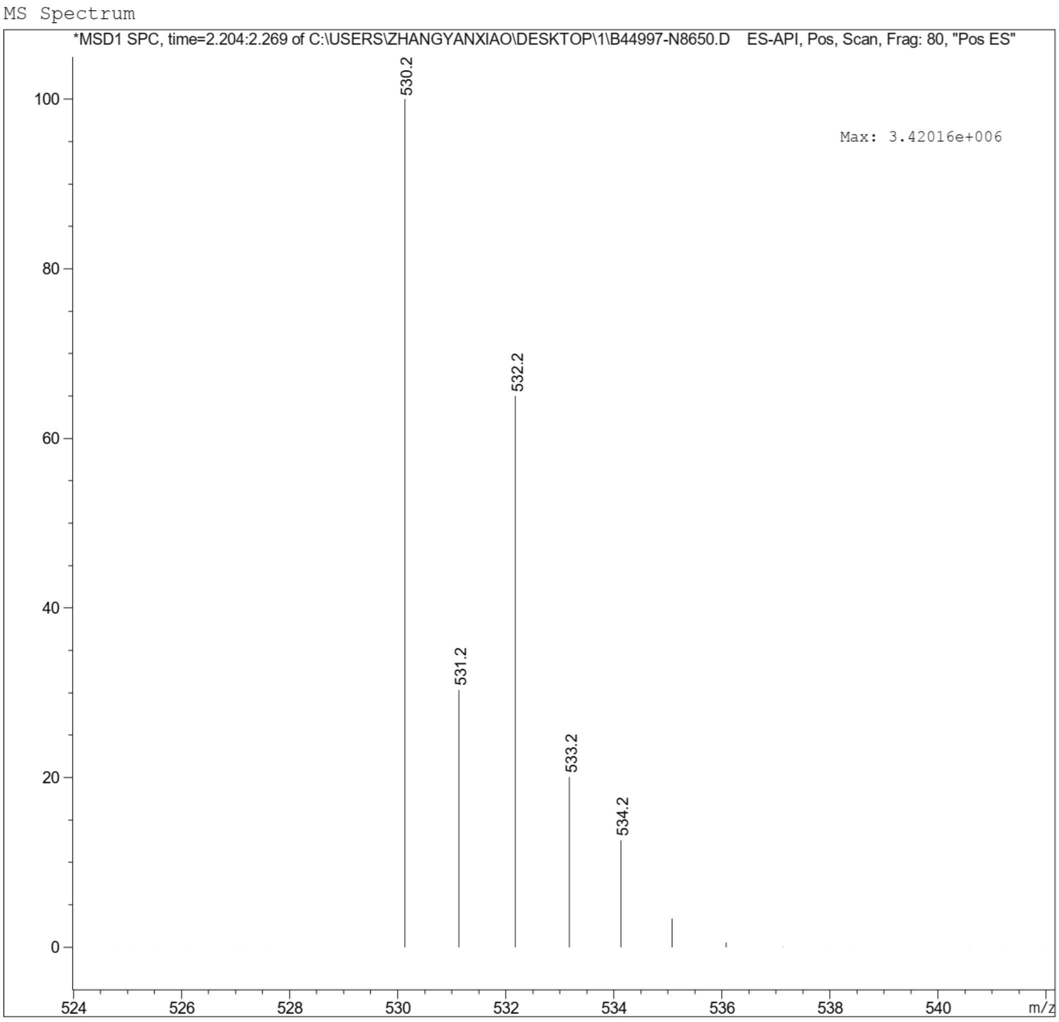

LC-MS of compound **H09**

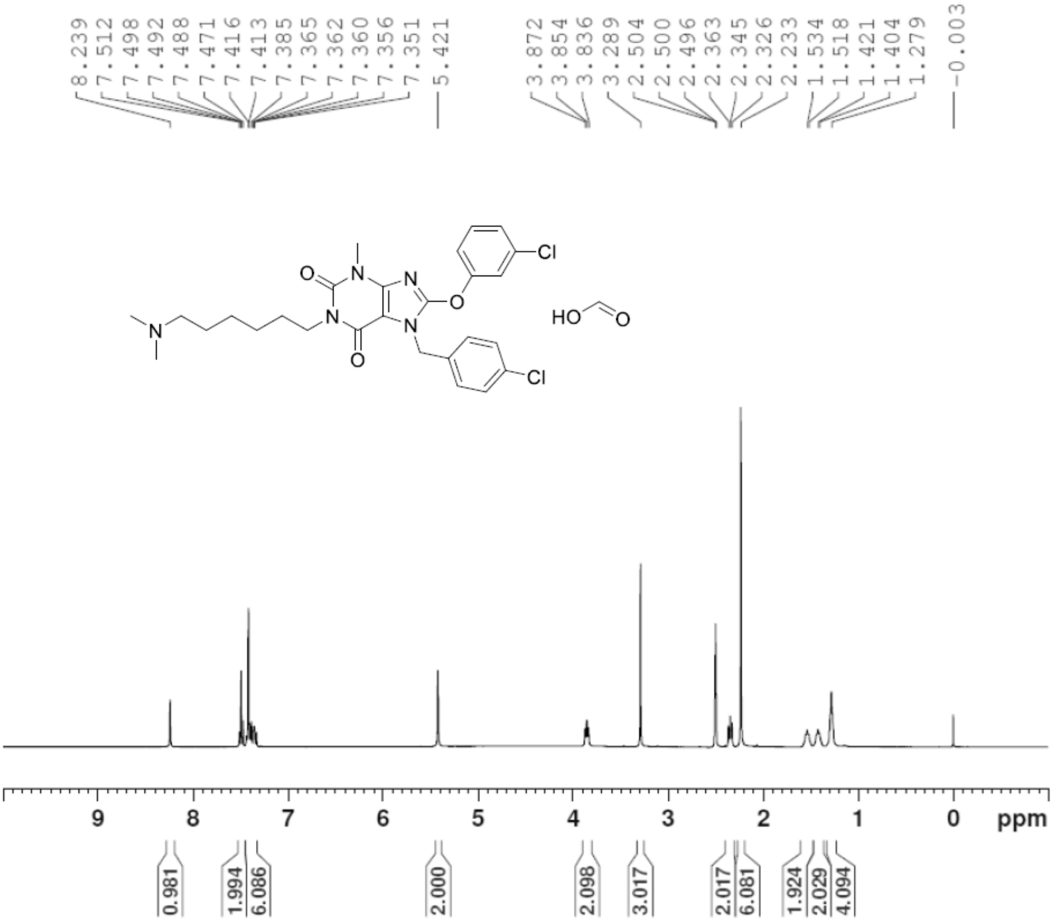

^{1}H NMR of compound **H10**

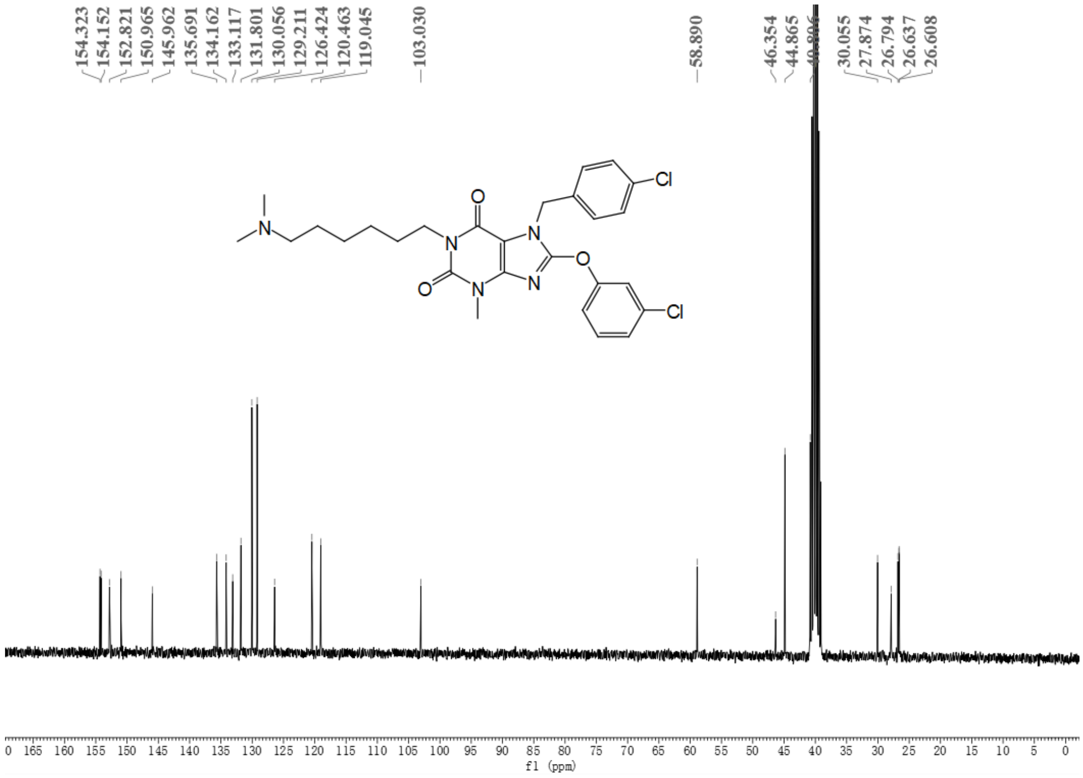

^{13}C NMR of compound **H10**

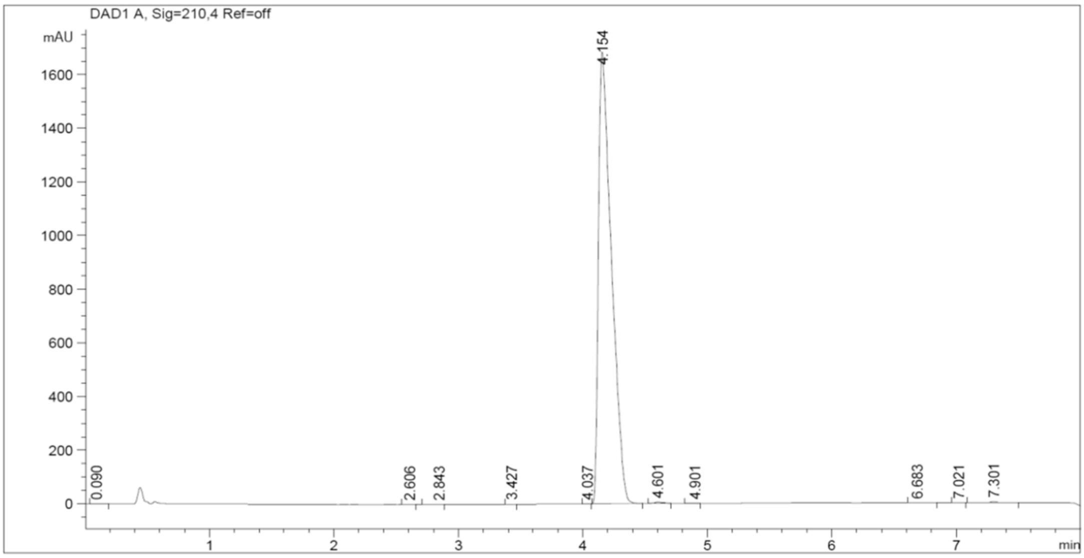

HPLC of compound **H10**

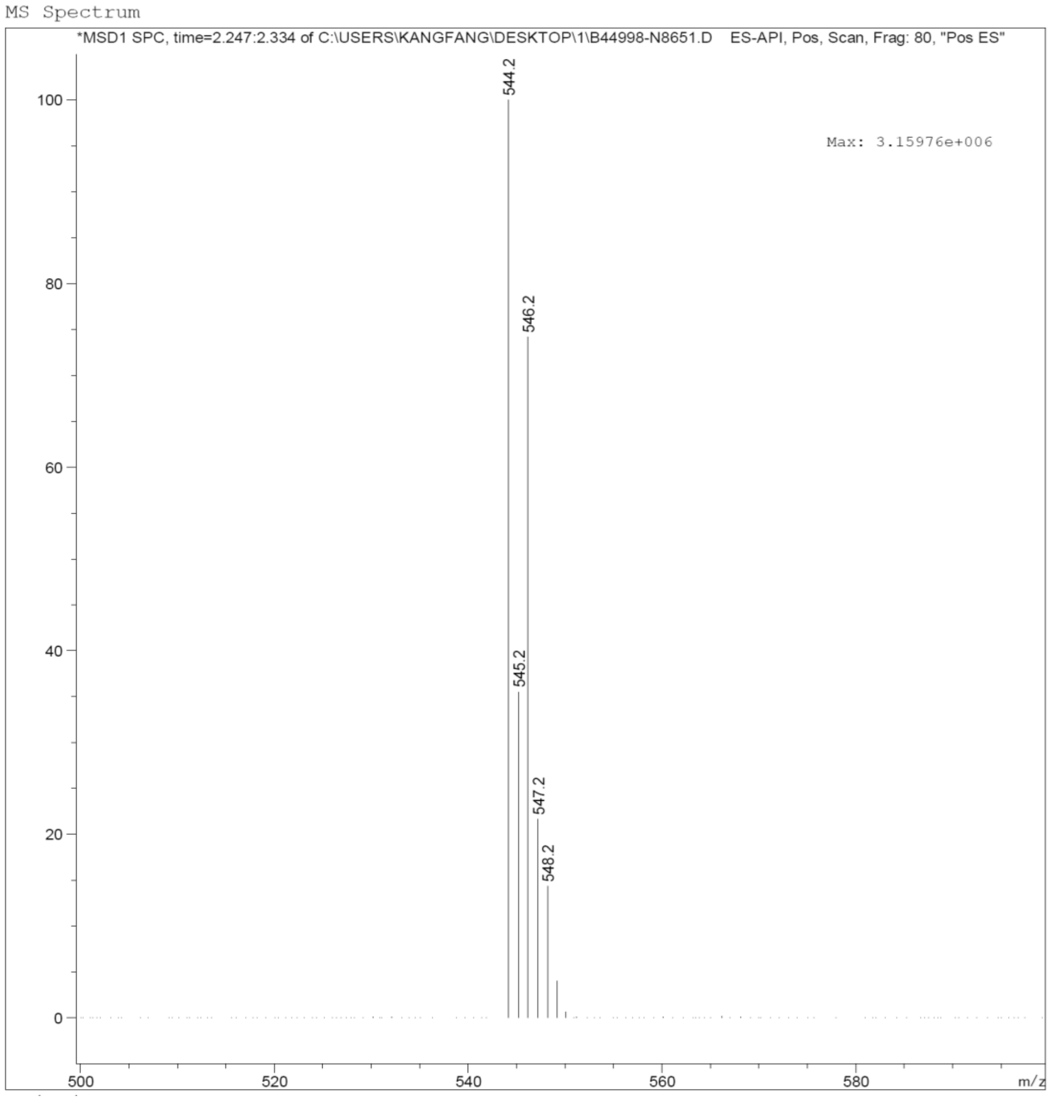

LC-MS of compound **H10**

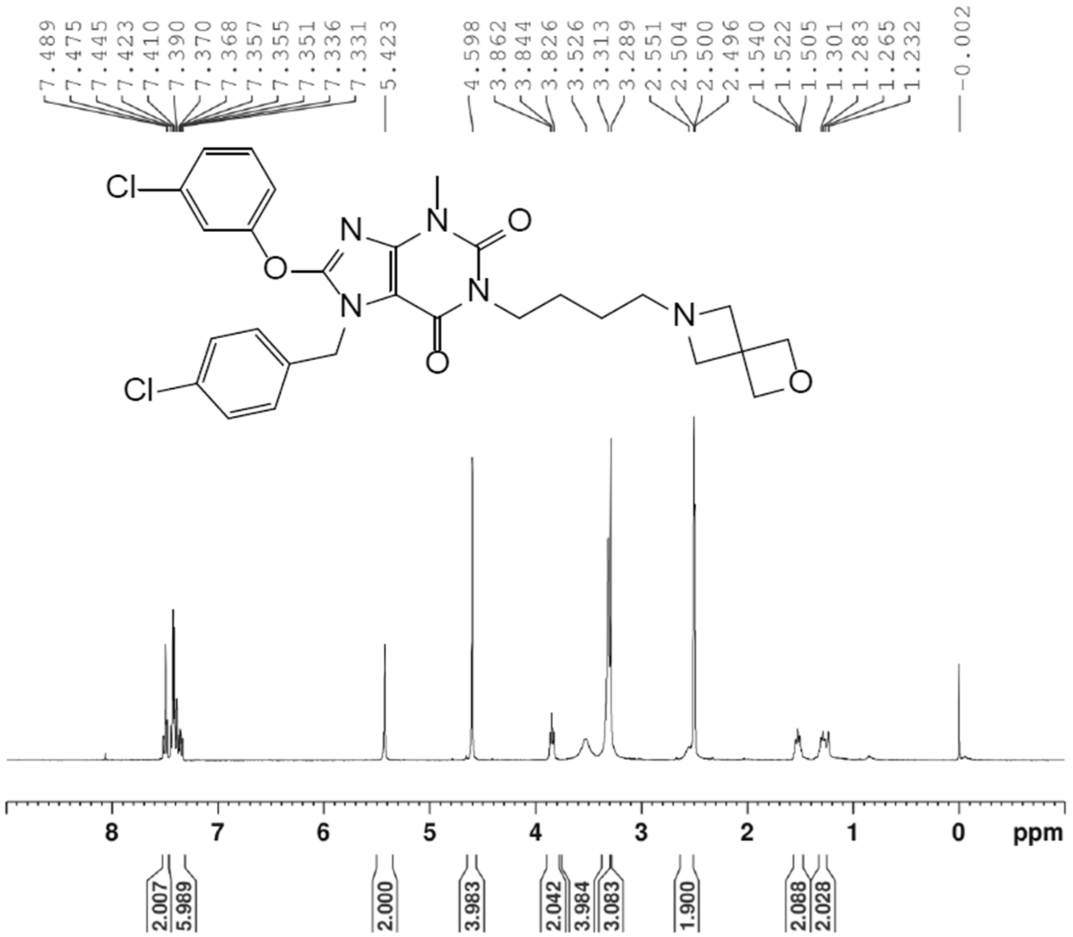

^{1}H NMR of compound **H11**

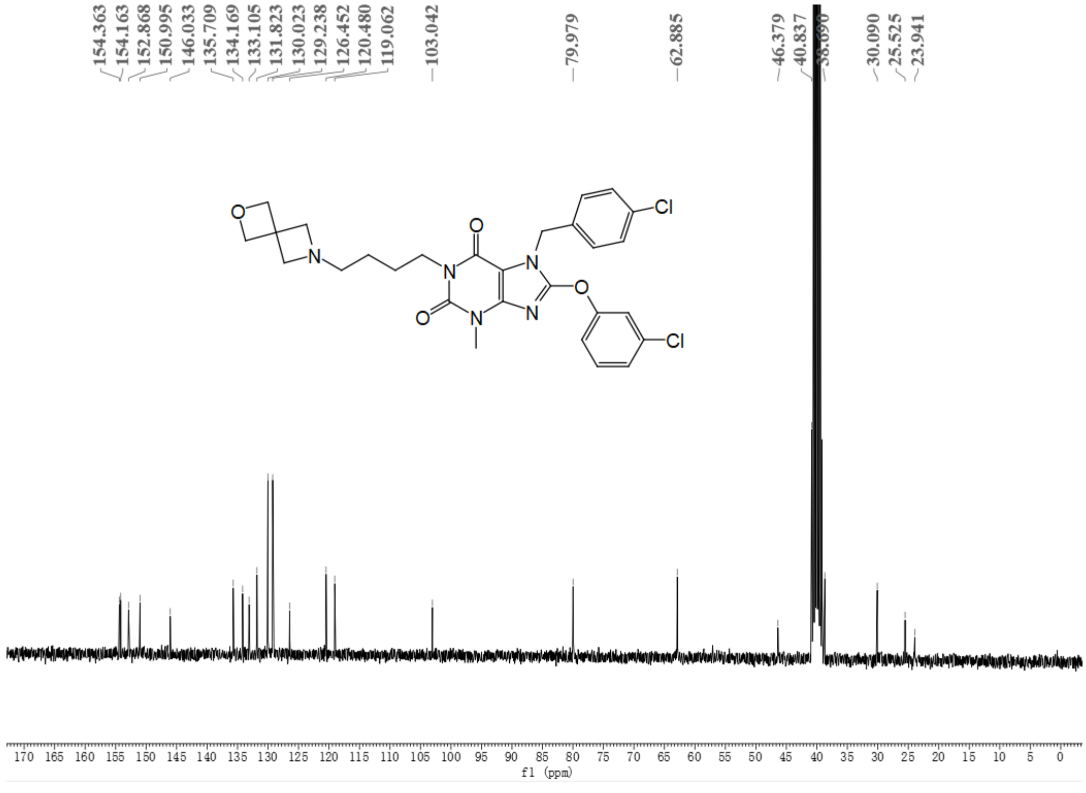

^{13}C NMR of compound **H11**

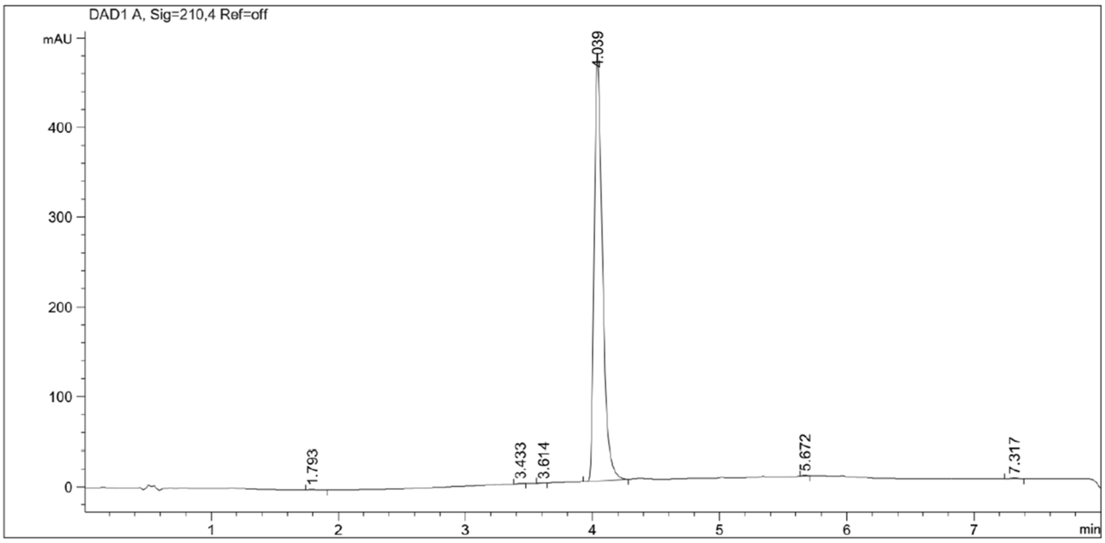

HPLC of compound **H11**

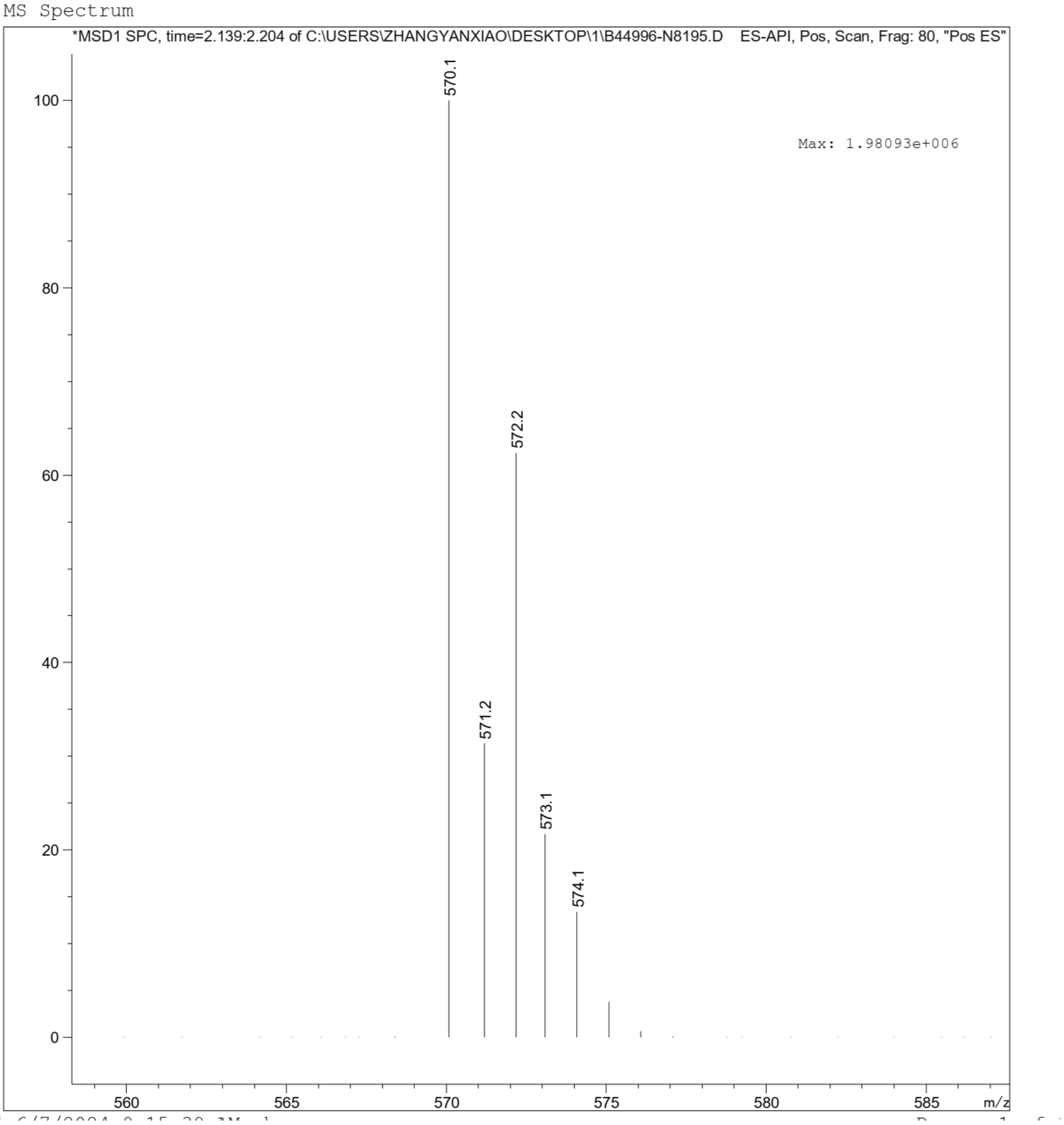

LC-MS of compound **H11**

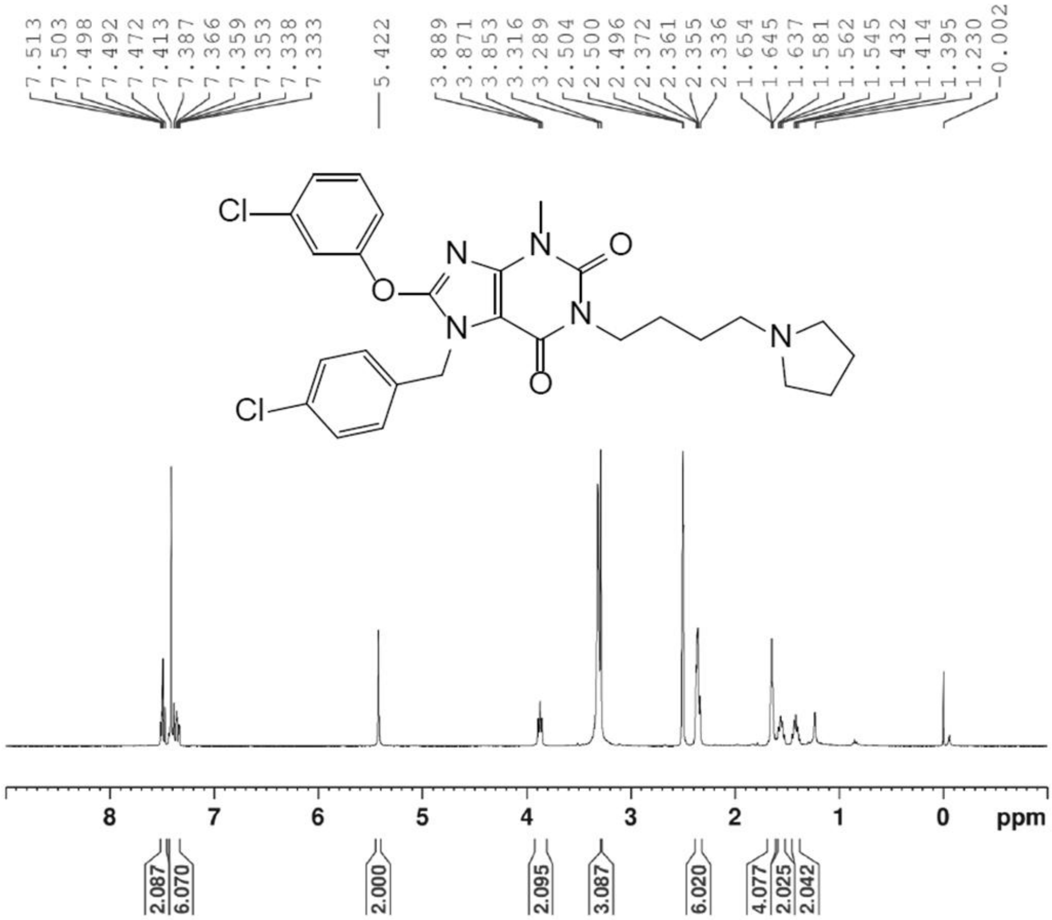

^{1}H NMR of compound **H12**

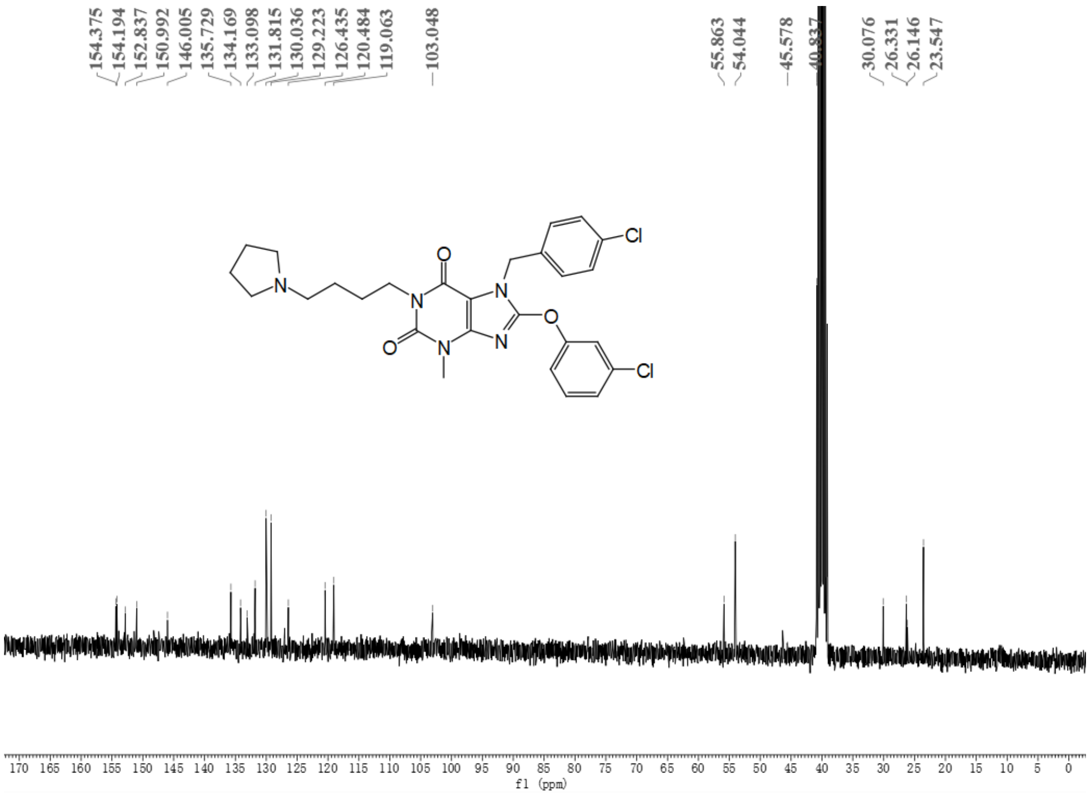

^{13}C NMR of compound **H12**

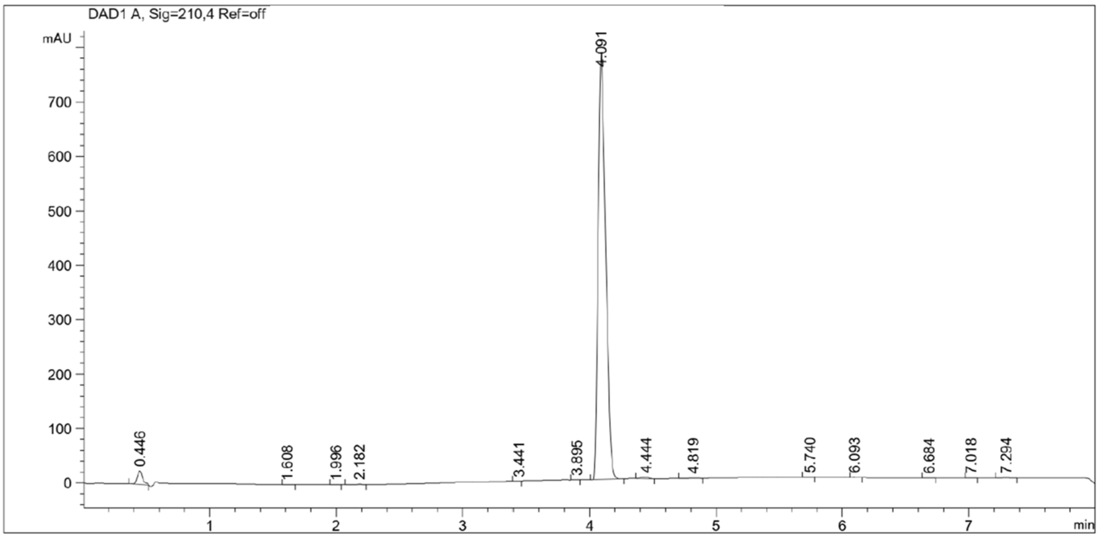

HPLC of compound **H12**

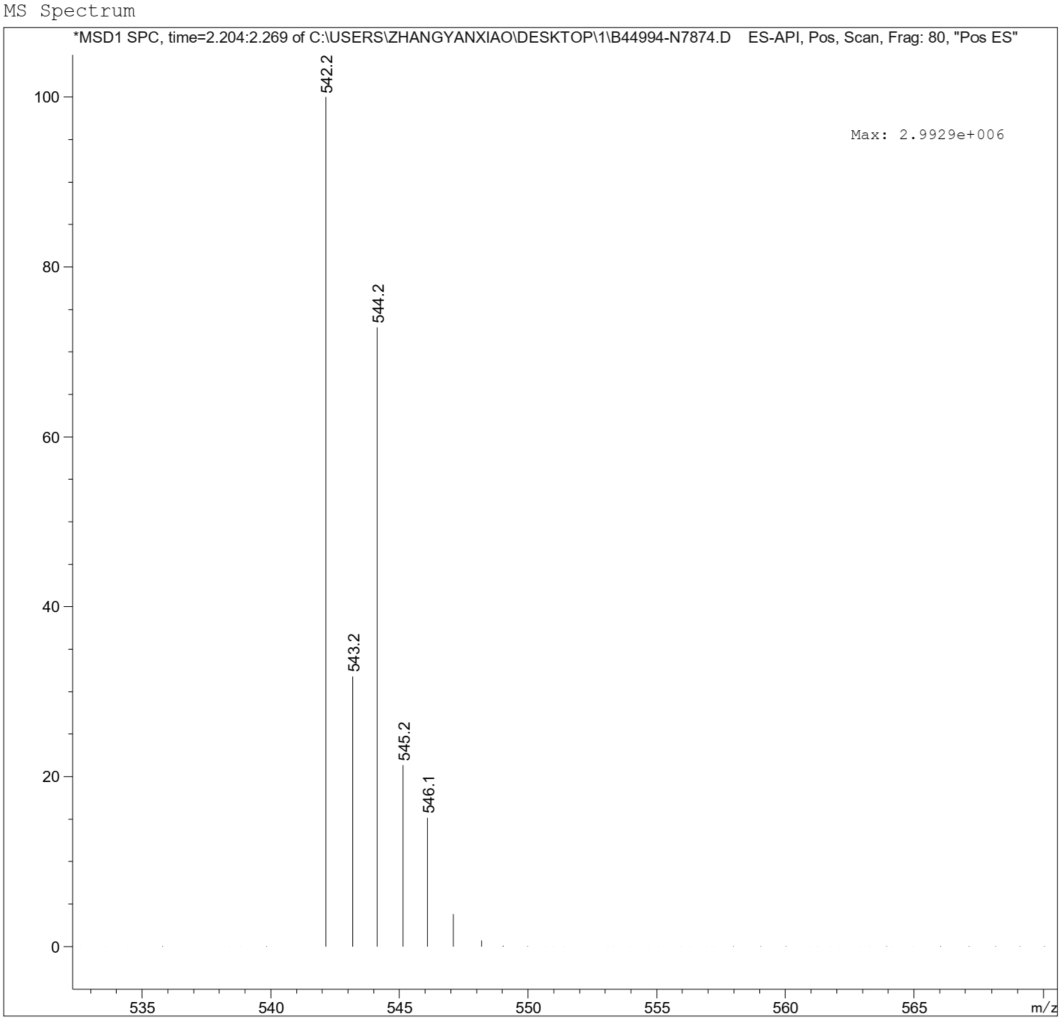

LC-MS of compound **H12**

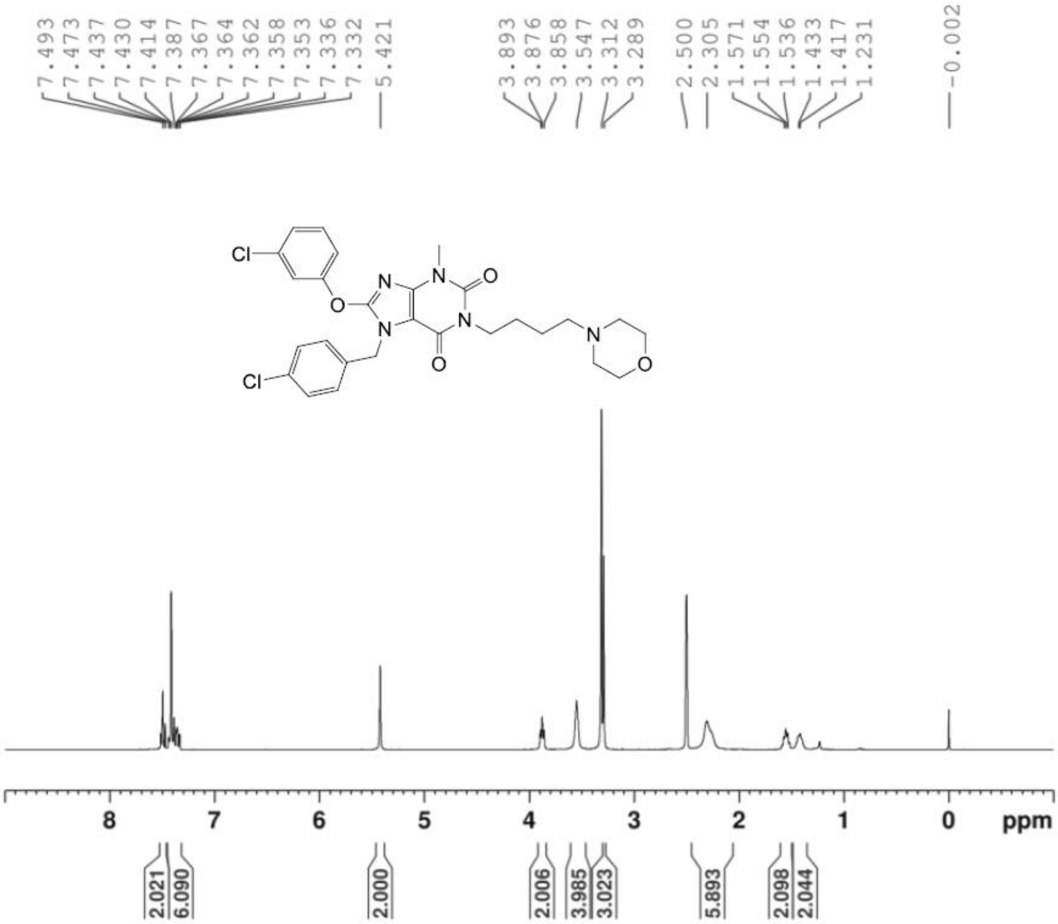

^{1}H NMR of compound **H13**

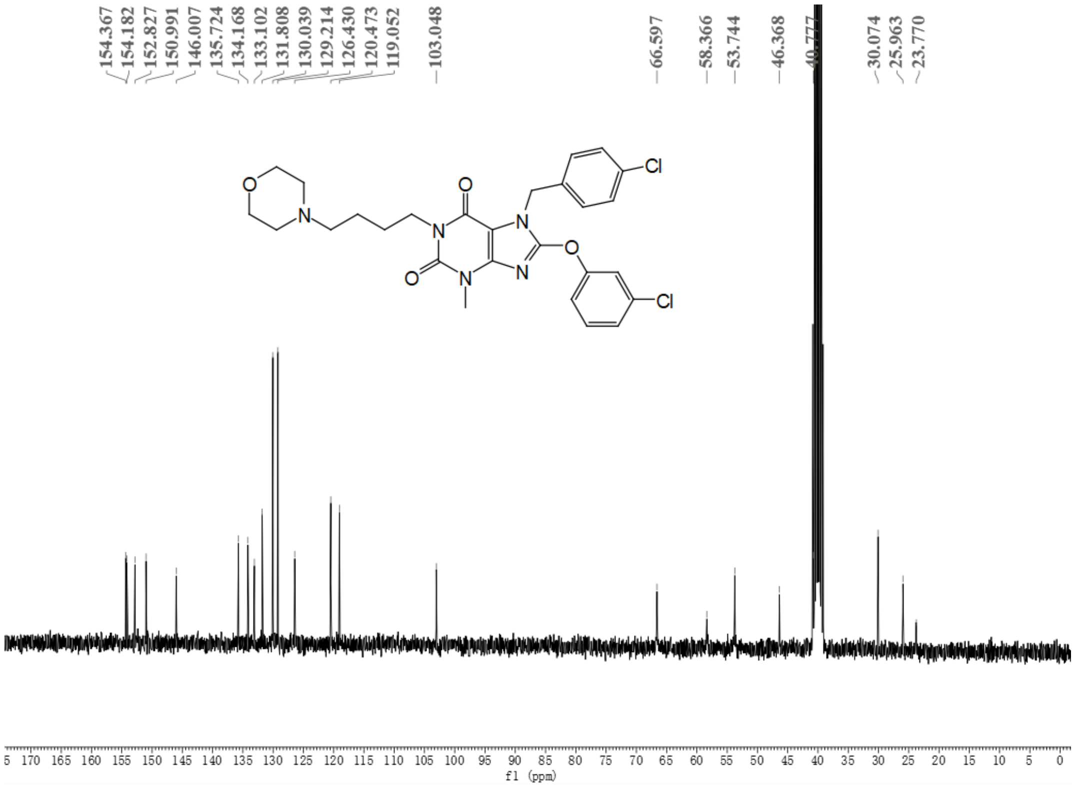

^{13}C NMR of compound **H13**

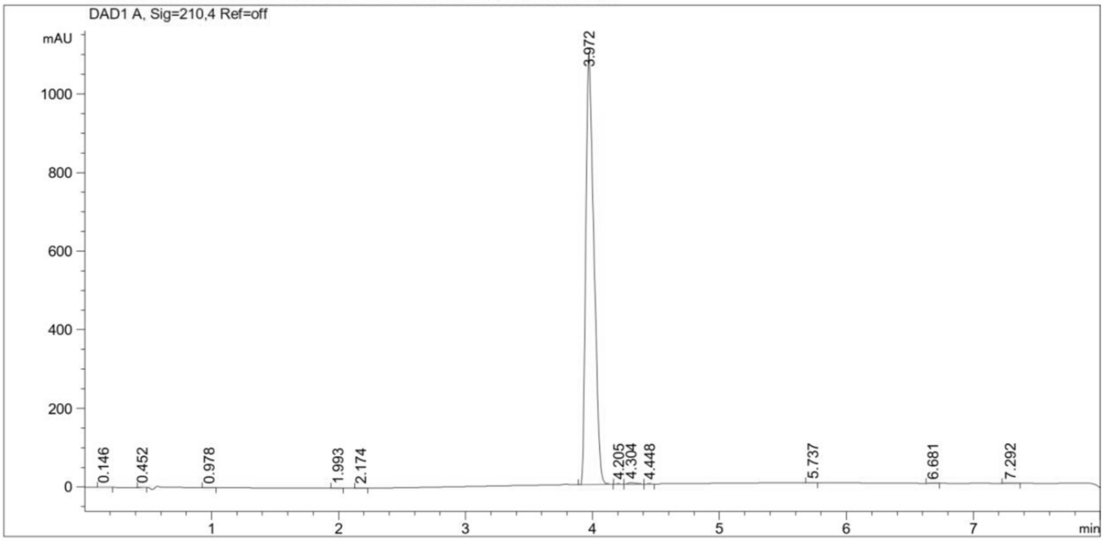

HPLC of compound **H13**

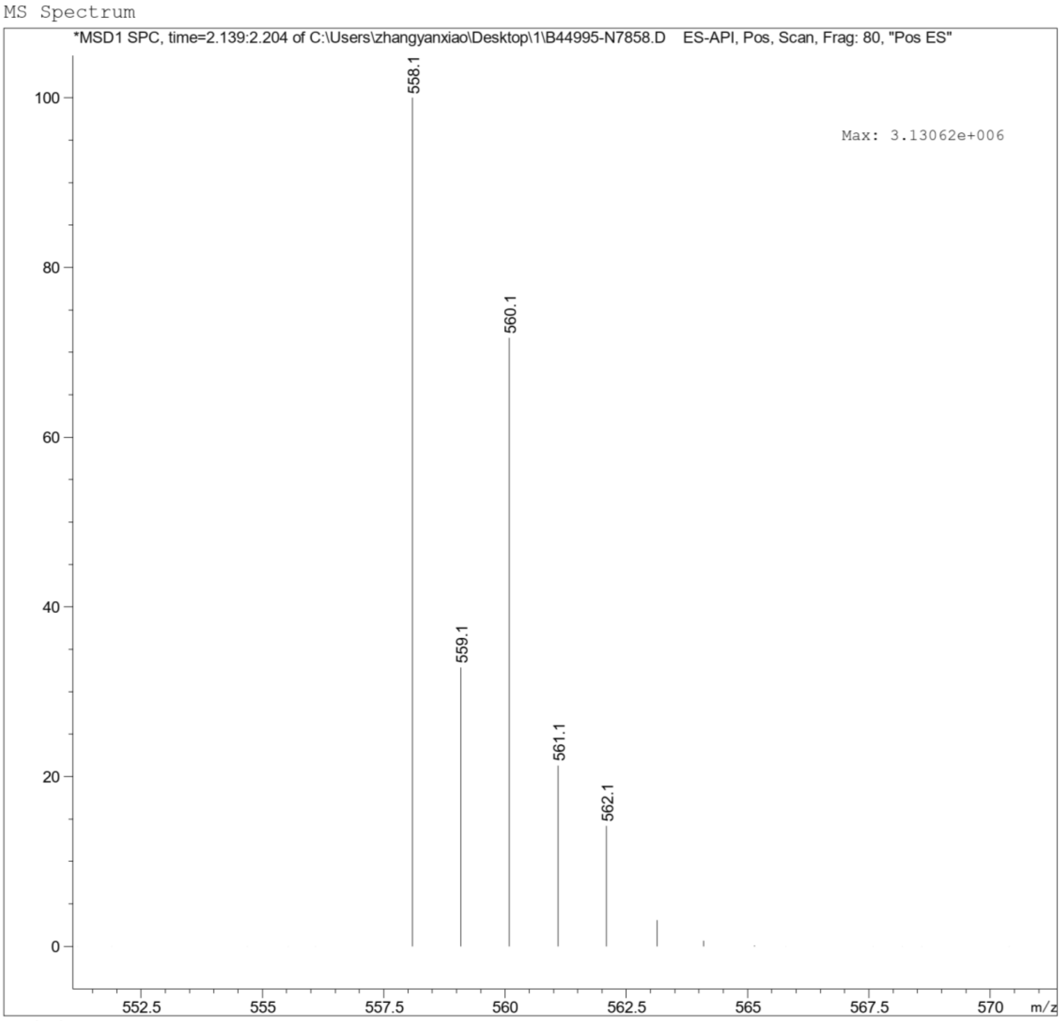

LC-MS of compound **H13**

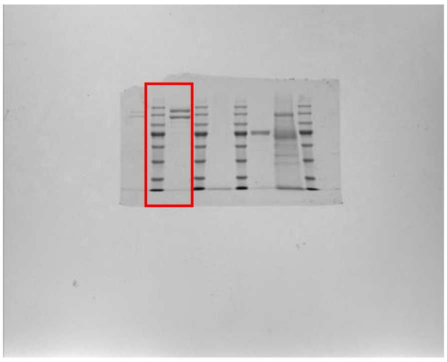

Uncropped scan of the gel shown in **Supplementary Figure 1**.
