# Supplementary material for: Cryo-EM Structure of the TRPC1/5 Heteromer Enables Design of Antidepressant and Anxiolytic Drug with Reduced Side Effects
Source: Nat Commun. 2026 May 23;17:6770. doi: 10.1038/s41467-026-73409-1 (PMC13385394; doi:10.1038/s41467-026-73409-1)
Supplement: Supplementary file 2 — Reporting Summary [file 41467_2026_73409_MOESM2_ESM.pdf]

nature portfolio

Corresponding author(s): DARP; Manuscript Number: NCOMMS-25-72449C

Last updated by author(s): Mar 6, 2026

Reporting Summary

Nature Portfolio wishes to improve the reproducibility of the work that we publish. This form provides structure for consistency and transparency in reporting. For further information on Nature Portfolio policies, see our Editorial Policies and the Editorial Policy Checklist.

Statistics

For all statistical analyses, confirm that the following items are present in the figure legend, table legend, main text, or Methods section.

n/a	Confirmed	
☐	☒	The exact sample size (*n*) for each experimental group/condition, given as a discrete number and unit of measurement
☐	☒	A statement on whether measurements were taken from distinct samples or whether the same sample was measured repeatedly
☐	☒	The statistical test(s) used AND whether they are one- or two-sided<br>*Only common tests should be described solely by name; describe more complex techniques in the Methods section.*
☒	☐	A description of all covariates tested
☒	☐	A description of any assumptions or corrections, such as tests of normality and adjustment for multiple comparisons
☐	☒	A full description of the statistical parameters including central tendency (e.g. means) or other basic estimates (e.g. regression coefficient) AND variation (e.g. standard deviation) or associated estimates of uncertainty (e.g. confidence intervals)
☐	☒	For null hypothesis testing, the test statistic (e.g. *F*, *t*, *r*) with confidence intervals, effect sizes, degrees of freedom and *P* value noted<br>*Give P values as exact values whenever suitable.*
☒	☐	For Bayesian analysis, information on the choice of priors and Markov chain Monte Carlo settings
☒	☐	For hierarchical and complex designs, identification of the appropriate level for tests and full reporting of outcomes
☒	☐	Estimates of effect sizes (e.g. Cohen's *d*, Pearson's *r*), indicating how they were calculated

Our web collection on statistics for biologists contains articles on many of the points above.

Software and code

Policy information about availability of computer code

Data collection	SerialEM 3.7 for cryo-EM, PatchMaster and pClamp 10.5 for electrophysiology, SoftMax Pro 7.1.2 for Calcium flux assay, ZEN 3.10 for Fluorescence resonance energy transfer (FRET) and 3DHistech (Pannoramic MIDI) system for RNAScope.
Data analysis	cryoSPARC v4.1.1 for cryo-EM. ChimeraX 1.8, WinCoot and Phenix for model building. MolProbity and Mtriage for Validation. UCSF Chimera 1.17.3, ChimeraX 1.8 and PyMOL for structural analysis and figure generation. PatchMaster, pClamp 10.5, Igor, GraphPad prism 10.1.2 and IBM SPSS Statistics 27 for electrophysiology. SoftMax Pro 7.1.2 and Excel for Calcium flux assay. IBM SPSS Statistics 27 and GraphPad prism 10.1.2 for FRET. CaseViewer for RNAscope. Schrödinger for molecular docking. IBM SPSS Statistics 27 and GraphPad prism 10.1.2 for animal studies.

For manuscripts utilizing custom algorithms or software that are central to the research but not yet described in published literature, software must be made available to editors and reviewers. We strongly encourage code deposition in a community repository (e.g. GitHub). See the Nature Portfolio guidelines for submitting code & software for further information.

Data

Policy information about availability of data

All manuscripts must include a data availability statement. This statement should provide the following information, where applicable:

- Accession codes, unique identifiers, or web links for publicly available datasets
- A description of any restrictions on data availability
- For clinical datasets or third party data, please ensure that the statement adheres to our policy

The atomic coordinates and cryo-EM maps generated in this study have been deposited in the Protein Data Bank (PDB) under accession code 9K4I (https://doi.org/10.2210/pdb9K4I/pdb) and the Electron Microscopy Data Bank (EMDB) under accession code EMD-62060 (https://www.ebi.ac.uk/emdb/EMD-62060). The map and model identifiers are detailed in Supplementary Table 1. Additional source data have been deposited in Figshare (https://doi.org/10.6084/m9.figshare.30171676) and are available under private embargo for peer review; the dataset will be made publicly available upon publication. Source data for all figures are also provided with this paper as a Source Data file.

Research involving human participants, their data, or biological material

Policy information about studies with human participants or human data. See also policy information about sex, gender (identity/presentation), and sexual orientation and race, ethnicity and racism.

Reporting on sex and gender	N/A
Reporting on race, ethnicity, or other socially relevant groupings	N/A
Population characteristics	N/A
Recruitment	N/A
Ethics oversight	N/A

Note that full information on the approval of the study protocol must also be provided in the manuscript.

Field-specific reporting

Please select the one below that is the best fit for your research. If you are not sure, read the appropriate sections before making your selection.

☒ Life sciences ☐ Behavioural & social sciences ☐ Ecological, evolutionary & environmental sciences

For a reference copy of the document with all sections, see nature.com/documents/nr-reporting-summary-flat.pdf

Life sciences study design

All studies must disclose on these points even when the disclosure is negative.

Sample size	Statistical methods were not used to determine sample size. Sample size for cryo-EM studies was determined by availability of microscope time and to ensure we obtain sufficient resolution for model building. For in vitro experiments (e.g., electrophysiology, calcium flux assays,FRET assays, binding assays), sample sizes (number of independent cells/replicates) were chosen based on established standards and common practices in the field and our prior experiences. These sample sizes have been consistently shown to be sufficient for detecting biologically relevant effects with statistical confidence in such experiments. For animal studies, the sample size (n = 10 or 6 per group was determined according to common practices in pharmacological research using the CUMS Model, the Anxiety Model and the Obesity Model, with the number of animals per group (n =10 or 6) being consistent with published studies that reliably demonstrate efficacy in this setting. Sex was considered in the study design. Only male mice were used, as the behavioral models (CUMS, anxiety) are best characterized in males and to reduce variability. Data are not disaggregated by sex as only males were studied.
Data exclusions	No data were excluded from the in silico or biochemical analyses. For electrophysiology and FRET, data points were only excluded based on pre-established technical criteria, such as loss of seal integrity during patch-clamp recordings or clear signs of cell death (e.g., excessive bleaching, lack of response to positive controls). These criteria were defined before data collection. For animal studies, during the acclimatization period, the general health condition of the animals was monitored daily. Any animals that were deemed unfit based on pre-established criteria (e.g., significant weight loss, signs of lethargy, or other health abnormalities) were excluded from the experiment.
Replication	Information on sample size is provided in figure legends throughout the manuscript.
Randomization	For animal studies, mice were randomly assigned to experimental groups (e.g., control vs. drug-treated) after baseline measurements were taken. Randomization was not applicable to in silico studies or in vitro experiments using recombinant systems/cell lines, as these do not involve organism-level variation.
Blinding	Blinding was not used in this study, because the knowledge of the sample doesn't affect the measurement of the datasets.

Reporting for specific materials, systems and methods

We require information from authors about some types of materials, experimental systems and methods used in many studies. Here, indicate whether each material, system or method listed is relevant to your study. If you are not sure if a list item applies to your research, read the appropriate section before selecting a response.

Materials & experimental systems

n/a	Involved in the study
☒	☐ Antibodies
☐	☒ Eukaryotic cell lines
☒	☐ Palaeontology and archaeology
☐	☒ Animals and other organisms
☒	☐ Clinical data
☒	☐ Dual use research of concern
☒	☐ Plants

Methods

n/a	Involved in the study
☒	☐ ChIP-seq
☒	☐ Flow cytometry
☒	☐ MRI-based neuroimaging

Eukaryotic cell lines

Policy information about cell lines and Sex and Gender in Research

Cell line source(s)	Sf9 cells was originally obtained from Thermo Fisher. HEK293S GnTI– and HEK293T cells were both from the American Type Culture Collection (ATCC).
Authentication	Cell lines used were not authenticated.
Mycoplasma contamination	Mycoplasma contamination was tested and found to be negative.
Commonly misidentified lines (See ICLAC register)	Commonly misidentified lines were not used in this study.

Animals and other research organisms

Policy information about studies involving animals; ARRIVE guidelines recommended for reporting animal research, and Sex and Gender in Research

Laboratory animals	Male C57BL/6J mice were used in this study. For CUMS and anxiety models: male C57BL/6J mice (7-8 weeks old) were obtained from Jinan Pengyue Laboratory Animal Breeding Co., Ltd. (Jinan, China). For obesity model: male C57BL/6J mice (14 weeks old) with high-fat diet-induced obesity were obtained from SPF (Beijing) Biotechnology Co., Ltd. (Beijing, China). All animals used were male C57BL/6J mice, with ages and sources specified for each experiment as above.
Wild animals	This study did not involve wild animals.
Reporting on sex	Male
Field-collected samples	This study did not involve samples collected from the field.
Ethics oversight	The experimental design and animal handling have been approved by the IACUC (Approval No: XZICE-IACUC-2024081602 and IACUC-B2024102-P-01) and will be strictly followed as per the approved protocol.

Note that full information on the approval of the study protocol must also be provided in the manuscript.

Plants

Seed stocks	N/A
Novel plant genotypes	N/A
Authentication	N/A
