# Supplementary material for: Cryo-EM Structure of the TRPC1/5 Heteromer Enables Design of Antidepressant and Anxiolytic Drug with Reduced Side Effects
Source: Nat Commun. 2026 May 23;17:6770. doi: 10.1038/s41467-026-73409-1 (PMC13385394; doi:10.1038/s41467-026-73409-1)
Supplement: Supplementary file 3 — Transparent Peer Review file [file 41467_2026_73409_MOESM3_ESM.pdf]

nature portfolio

Peer Review File

Cryo-EM Structure of the TRPC1/5 Heteromer Enables Design of Antidepressant and Anxiolytic Drug with Reduced Side Effects

Corresponding Author: Professor Jin Zhang

This file contains all reviewer reports in order by version, followed by all author rebuttals in order by version.

Version 0:

Reviewer comments:

Reviewer #1

(Remarks to the Author)
TRPC1/5 heteromer play many important physiological roles, yet their structure remains unknown. In this manuscript, Chen et al. determined the structure of the TRPC1/5 1:3 heterotetramer. Moreover, they developed small molecular inhibitor targeting C1/C5 heteromer to reduce the side effect of TRPC5 inhibitors. This work builds upon previous research on C1/C4 heteromer by the other groups (Won et al., Nat. Struct. Mol. Biol., 2024, PMID: 39478185) and is important to the TRPC community.

I have several questions regarding the findings presented in the manuscript:
1. In Figures 1D and 1E, I cannot locate the label corresponding to C1.
2. In Figure 1B, the mRNA of C1 and C5 may have different translational efficiencies. Additionally, C1 and C5 might exhibit distinct protein half-lives. Therefore, determining the stoichiometry solely based on the cDNA transfection ratio may not be accurate. I suggest revising the conclusion for Figure 1B to state that "co-expression of C1 shifts the EC_{50} value of EA on C5."
3. Since the authors have already resolved the structure of the C1/C5 heteromer, could they provide an explanation for why the C1/C5 heteromer exhibits a higher EC_{50} value for EA compared to the C5 homomer?
4. The authors have developed new inhibitors targeting the C1/C5 heteromer. The dose-response curve appears unusually steep compared to other compounds. It would be helpful if the authors could provide the Hill coefficient and offer an explanation for this observation.
5. What is the selectivity profile of the new inhibitors against the C1/C4 heteromer or the C4 homomer?
6. Please cite the recent Phase II clinical trial results for BI 1358894 (Shelton et al., Journal of Clinical Psychiatry, 2025; PMID: 40900110) to provide an updated reference.
7. It would be nice for the authors to provide pdb and maps during revision for quality assessment.

Reviewer #2

(Remarks to the Author)
This study presents cryo-electron microscopy (cryo-EM) analysis of the TRPC1/5 heteromer, resolving its high-resolution structure and elucidating unique ligand-binding and functional regulatory mechanisms. Based on these structural findings, the authors developed the small-molecule inhibitor JD03-02, demonstrating its efficacy in animal models of depression and anxiety with reduced side effects compared to conventional treatments. While the study provides valuable insights, the current manuscript lacks sufficient novelty and is premature and mechanistic depth for publication in a high-impact journal. The following major revisions are required to strengthen the work:

1) Structural Limitations of Cryo-EM Data
The current cryo-EM analysis is limited to the ligand-free conformation of TRPC1/5. To fully understand the functional dynamics, structural data on activated or ligand-bound states are essential. The authors should explicitly acknowledge this limitation in the Discussion and propose future experimental directions (e.g., cryo-EM of agonist-bound states or functional

assays).

2) Mechanistic Clarification in Behavioral Studies
The differences between JD03-02 and classical antidepressants (e.g., fluoxetine) in CUMS, FST, and SPT experiments require deeper mechanistic explanation.

3) The Discussion should address potential factors such as:
Target specificity (TRPC1/5 vs. monoaminergic systems)
Pharmacokinetic properties (e.g., half-life, distribution)
Blood-brain barrier permeability (quantitative comparisons if available)

4) Strengthening Mechanistic Interpretations
While a novel binding pocket at the TRPC1-TRPC5 interface is identified, the proposed "negative regulatory" role of TRPC1 in channel gating and calcium permeability remains speculative. Additional experiments are needed:
Electrophysiological recordings of TRPC1/5 vs. TRPC5 homomers
Molecular dynamics simulations of gating mechanisms
Calcium imaging studies with TRPC1 knockdown/knockout controls

5) Agonist Selection Rationale
The use of Englerin A as an agonist in TRPC1/5 stoichiometry studies is problematic, given its established preference for TRPC4/5. The authors must:
Justify this experimental choice with supporting literature
Include controls to rule out TRPC5 homomer contributions (e.g., TRPC1-specific knockdowns)
Consider alternative agonists (e.g., OAG) for validation

6) Technical Corrections
Standardize IC_{50} formatting (e.g., "IC_{50} = 6.7 nM")
Clarify the relevance of Figure 10 (H08's TRPC1/4 selectivity) to the TRPC1/5 focus
Provide high-resolution versions of all figures

7) Expanded Discussion Requirements
The Discussion must now include:
Comparative analysis of JD03-02's pharmacokinetics vs. reference compounds
Toxicity profile discussion (e.g., off-target effects)
Clinical translation potential (e.g., dosing considerations, therapeutic window)

8) Selectivity Data Gap
While JD03-02 shows potent inhibition of TRPC1/5 and TRPC5, selectivity against other TRPC subtypes (e.g., TRPC3, TRPC6) must be demonstrated experimentally. Include:
Dose-response curves for at least 3 additional TRPC subtypes
Selectivity index calculations

9) Figure Improvements
Simplify Extended Data figures with schematic summaries
Add a comparative diagram of TRPC1/5 vs. TRPC5 structural/functional features

Reviewer #3

(Remarks to the Author)
Based on high-resolution structural information about TRPC1/5 heterotetramers obtained at 2.8Å resolution, the authors designed a high-affinity antagonist, named JD03-02, that was highly selective in blocking TRPC1/5 complexes over TRPC5 homomeric channels. Specifically, the newly synthesized compound JD03-02 exhibited >10,000-fold selectivity for TRPC1/5 over TRPC5 homomers, thus allowing selective blockade of TRPC1/5 heteromers. At the functional level, the authors found that systemic administration of JD03-02 produced anxiolytic and antidepressant effects in mice. This is an interesting study providing a good example of how understanding the ion channels' molecular architecture could be used for structure-based drug design. More specifically, the reported findings suggest that TRPC channels with different subunit composition could differentially contribute to behavioral mechanisms. For the most part, the studies are well designed and results are interesting. In my view, however, there are certain issues with the present work which should be resolved before it could be recommended for publication.

The description of behavioral studies is somewhat confusing. It would be helpful if the authors could include a diagram showing at what time points relative to the behavioral testing the pharmacological interventions were performed. Although it's not immediately clear which experimental groups were compared. It should be explicitly stated in the manuscript and statistical results should be fully reported, including exact P and t values.

The sample sizes were relatively small, so t-test might be not appropriate if the data points are not normally distributed. Was the normalcy of the fata points distribution evaluated?

Another issue is that behavioral results are not fully reported. For the open field test both the number of entries to the center

and time spent in the center should be reported. In the EPM test, both number of open arm entries and time spent in open arms should be included.

It is implied in the manuscript that continuous (lasting for 11-14 days) daily treatment with orally-administered JD03-02 was more efficient in reducing CUMS-induced depressive-like behaviors in mice. However, there is no any discussion in the manuscript how this might work. Was the difference between behavioral effects of a single dose of JD03-02 statistically different from the effects of continuous drug delivery? How long-lasting was the blocking effect of JD03-02 on TRPC1/5 heteromers? What is actually evidence here that behavioral improvements following JD03-02 administration were exclusively due to the blockade of TRPC1/5 channels?

The manuscript does include any data on how the blockade of TRPC1/5 heteromers could affect the neuronal circuits controlling anxiety and/or depression. Does it lead to changes in neuronal excitability or synaptic transmission in specific pathways, or both?

The systemic delivery of JD03-02 in behavioral experiments might be suboptimal, and region-specific infusions of the antagonist could strengthen the study.

Version 1:

Reviewer comments:

Reviewer #1

(Remarks to the Author)
Q2: I am still not convinced that the shift in EC_{50} alone is sufficient to define the specific assembly of the TRPC1-C5 heteromer. Consider an alternative scenario: TRPC1 and C5 subunits assemble randomly, but TRPC1 homomers are non-functional. In this case, the observed TRPC1-C5 heteromeric population could exhibit a range of EA EC_{50} values, with the TRPC5 homomer (3.6 nM) at one extreme and a complex containing three TRPC1 subunits and one TRPC5 subunit (e.g., ~60 nM) at the other. Biasing the transfection ratio towards 3:1 or 6:1 (TRPC1:C5) would increase the proportion of complexes with a higher TRPC1 content, which aligns well with the results in Fig. 1b. For the current manuscript, the inclusion of the FRET results has greatly strengthened this section, and I have no further questions on this point.
Q3: The decreased affinity of EA at the TRPC1-C5 interface, inferred from structural data, correlates with its reduced functional potency. However, does this imply that EA must bind to all four subunits to open the channel? Could the authors provide the Hill coefficients for EA activation of TRPC5 homomers and TRPC1-C5 heteromers?
Furthermore, two other highly relevant studies should be cited:
https://www.biorxiv.org/content/10.1101/2025.07.09.663840v1
https://www.nature.com/articles/s41467-025-67024-9
Q4: The authors propose that JD03-02 (H08) binds with high affinity at the TRPC1-C5 interface but with low affinity at the TRPC5-C5 interface. If this model and negative cooperativity hold, the Hill coefficient for JD03-02 on the TRPC1/C5 heteromer should be close to 1, while its coefficient on the TRPC5 homomer should be similar to those of other inhibitors. The authors should re-examine and potentially repeat these experiments to verify this point.

Reviewer #2

(Remarks to the Author)
The authors have comprehensively addressed all reviewer concerns, and the manuscript is now suitable for publication in its current form.

Reviewer #3

(Remarks to the Author)
The authors responded well to the reviewers comments. The manuscript was adequately revised and is suitable for publication in its present form.

Version 2:

Reviewer comments:

Reviewer #1

(Remarks to the Author)
I have no more questions.

Open Access This Peer Review File is licensed under a Creative Commons Attribution 4.0 International License, which permits use, sharing, adaptation, distribution and reproduction in any medium or format, as long as you give appropriate credit to the original author(s) and the source, provide a link to the Creative Commons license, and indicate if changes were made.
In cases where reviewers are anonymous, credit should be given to 'Anonymous Referee' and the source.
The images or other third party material in this Peer Review File are included in the article's Creative Commons license, unless indicated otherwise in a credit line to the material. If material is not included in the article's Creative Commons license and your intended use is not permitted by statutory regulation or exceeds the permitted use, you will need to obtain permission directly from the copyright holder.
To view a copy of this license, visit https://creativecommons.org/licenses/by/4.0/

REVIEWER COMMENTS

Reviewer #1 (Remarks to the Author):

TRPC1/5 heteromer play many important physiological roles, yet their structure remains unknown. In this manuscript, Chen et al. determined the structure of the TRPC1/5 1:3 heterotetramer. Moreover, they developed small molecular inhibitor targeting C1/C5 heteromer to reduce the side effect of TRPC5 inhibitors. This work builds upon previous research on C1/C4 heteromer by the other groups (Won et al., Nat. Struct. Mol. Biol., 2024, PMID: 39478185) and is important to the TRPC community.

To Reviewer #1:

We sincerely thank the reviewer for the positive and insightful evaluation of our work and for recognizing its significance to the TRPC field. We greatly appreciate the constructive comments, which have helped us to improve both the clarity and rigor of the manuscript. As noted by the reviewer, heteromeric assembly is a common and functionally critical feature of ion channels, yet remains underexplored in drug discovery. We hope that our study not only advances understanding of TRPC1/5 heteromers but also provides a general framework for developing heteromer-selective ion channel therapeutics with reduced side effects.

We address each comment in detail below.

I have several questions regarding the findings presented in the manuscript:

1. In Figures 1D and 1E, I cannot locate the label corresponding to C1.

Response: We thank the reviewer for pointing out this labeling oversight. In the revised manuscript, we have carefully corrected Figures 1D and 1E (Figure 1d and 1e) and now explicitly label both the TRPC1 and TRPC5 subunits to ensure clarity. The updated figures have been inserted into the revised manuscript.

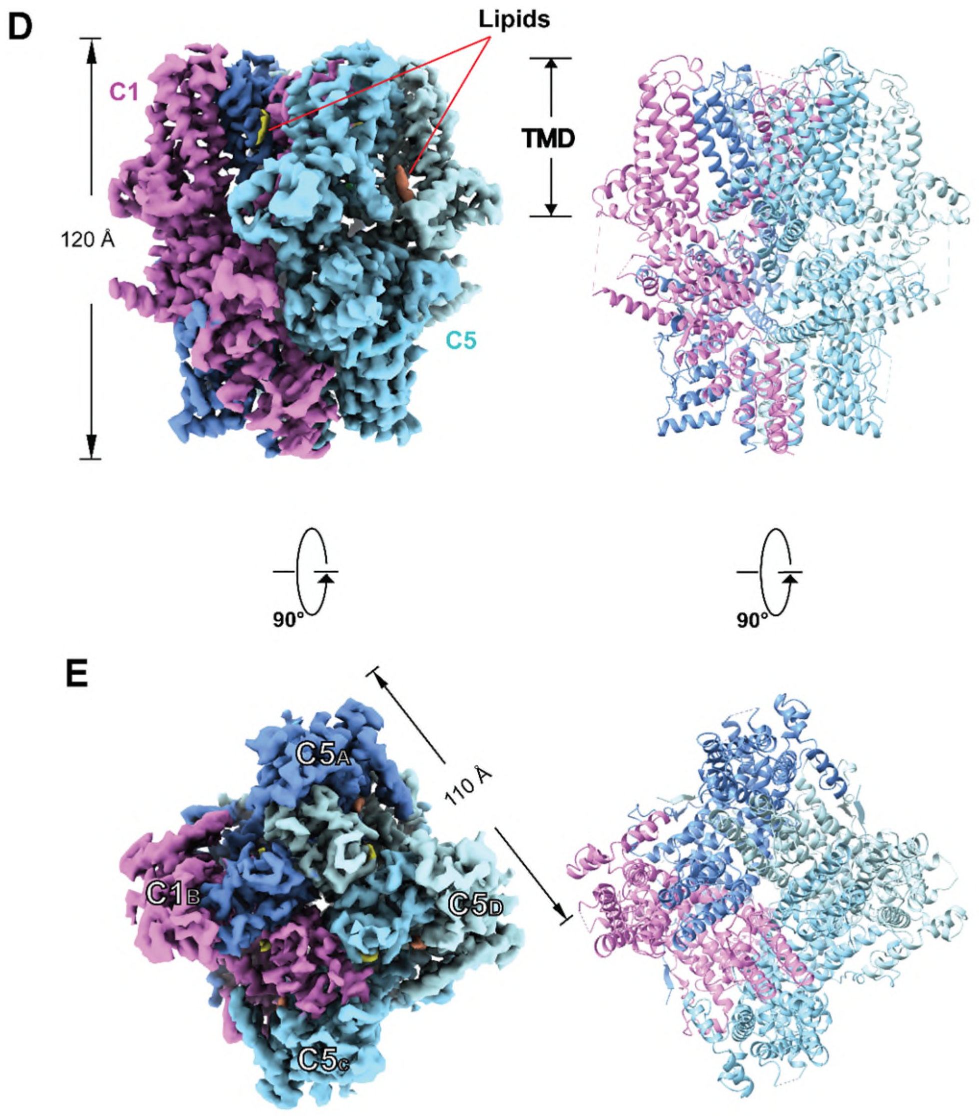

Figure 1d and 1e.

2. In Figure 1B, the mRNA of C1 and C5 may have different translational efficiencies. Additionally, C1 and C5 might exhibit distinct protein half-lives. Therefore, determining the stoichiometry solely based on the cDNA transfection ratio may not be accurate. I suggest revising the conclusion for Figure 1B to state that "co-expression of C1 shifts the EC_{50} value of EA on C5."

Response: We thank the reviewer for this insightful and important comment. We fully agree that inferring subunit stoichiometry solely from cDNA transfection ratios has inherent limitations, as TRPC1 and TRPC5 may differ in translational efficiency and protein stability.

Importantly, however, our primary observation is not the absolute expression level of each subunit, but rather the systematic and saturable shift in the EC_{50} of EA as the TRPC1 cDNA ratio is increased. Specifically, the EC_{50} value progressively shifts and reaches a stable plateau when the TRPC1:TRPC5 transfection ratio exceeds 6:1. Even if TRPC1 and TRPC5 are expressed at different efficiencies, the emergence of a clear plateau behavior strongly argues against random assembly. If heteromer formation were stochastic, one would expect a continuous, approximately linear drift in EC_{50} values rather than convergence to a stable limit.

Our experimental strategy is consistent with approaches widely used in classical heteromeric channel studies, such as GlyR α/β assembly (Yu et al., *Neuron*, 2021; PMID: 34473954), which face similar methodological constraints.

To further strengthen our conclusion, we performed FRET-based subunit interaction experiments (Figure 1c and Supplementary Fig. 2). These experiments show robust FRET between fluorescently tagged TRPC1 and TRPC5, with efficiencies comparable to those observed for TRPC5 homomers. In contrast, TRPC1–TRPC1 FRET is minimal in the presence of TRPC5. Together, these results provide independent and complementary evidence that the dominant heteromeric complex consists of one TRPC1 subunit and three TRPC5 subunits, consistent across both in vitro and cellular assays.

Following the reviewer's suggestion, we have revised the text to more precisely reflect the experimental conclusion. The revised statement now reads:

"Our dose-response experiments demonstrate that co-expression of TRPC1 induces a concentration-dependent rightward shift in the EC_{50} of EA for TRPC5-mediated currents. The EC_{50} value reached a plateau when the cDNA transfection ratio of TRPC1 to TRPC5 was increased to 6:1. This observation indicates that TRPC1 and TRPC5 assemble with a fixed, non-random stoichiometry in our HEK293 cell expression system." (Page 6, lines 160-165)

Figure 1c

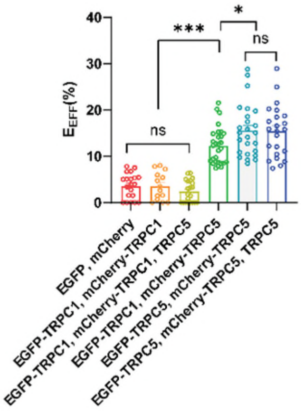

Supplementary Fig.2

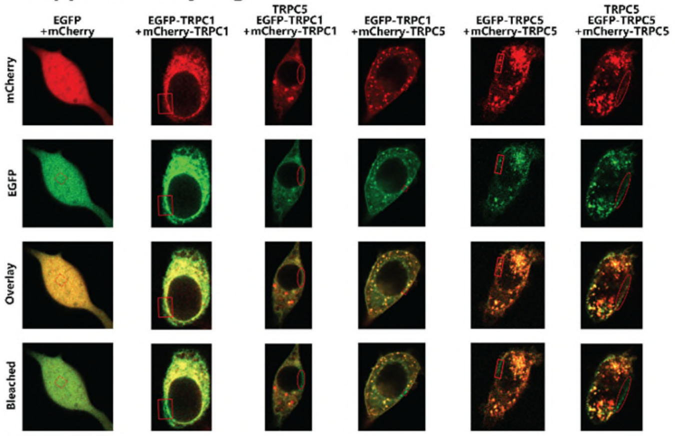

3. Since the authors have already resolved the structure of the C1/C5 heteromer, could they provide an explanation for why the C1/C5 heteromer exhibits a higher EC_{50} value for EA compared to the C5 homomer?

Response: We thank the reviewer for this excellent mechanistic question. To address this point, we performed molecular docking of EA into the TRPC1/5 heteromeric structure and compared the resulting binding mode with the EA–TRPC5 homomer complex previously resolved by us (Chen et al., *Protein Science*, 2025, PMID; 40671342).

This comparison reveals a key difference within the ligand-binding pocket. In the TRPC1/5 heteromer, residues F592 and W596 from the TRPC1 subunit replace Q573 and W577 from TRPC5, respectively. This substitution disrupts hydrogen bond interactions between the terminal hydroxyacetyl group of EA and Q573/W577 observed in the TRPC5 homomer. The loss of these stabilizing polar contacts likely weakens EA binding affinity, providing a structural explanation for the increased EC_{50} observed in the TRPC1/5 heteromer. The corresponding structural model and interaction analysis are shown in the revised figure.

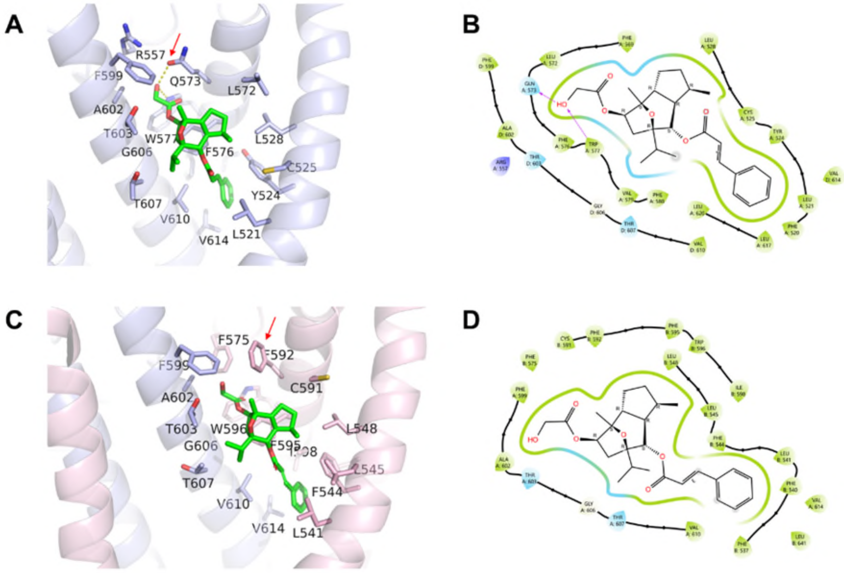

4. The authors have developed new inhibitors targeting the C1/C5 heteromer. The dose-response curve appears unusually steep compared to other compounds. It would be helpful if the authors could provide the Hill coefficient and offer an explanation for this observation.

Response: We appreciate this helpful suggestion. In the revised manuscript, we have now included Hill slope values for all relevant dose–response curves. JD03-02 exhibits minimal inhibition of TRPC5 homomers at low concentrations and only becomes inhibitory at higher concentrations (>5 μM). Correspondingly, the dose–response curve for TRPC5 homomers is steep, with a Hill slope >1, indicating a sharp transition in inhibitory effect over a narrow concentration range.

In contrast, the dose–response curves for TRPC1/5 heteromers display significantly lower Hill slopes (0.4–0.6), indicating a more gradual and progressive response. Notably, inhibition of the TRPC1/5 heteromer approaches saturation at relatively low concentrations, consistent with higher functional sensitivity and heteromer-selective engagement by JD03-02. These differences further support the distinct pharmacological profiles of the heteromer versus homomeric channels.

5. What is the selectivity profile of the new inhibitors against the C1/C4 heteromer or the C4 homomer?

Response: This important point is addressed in Supplementary Figure 9**.** JD03-02

inhibits the TRPC1/4 heteromer with an IC_{50} of 6.675 nM, whereas its IC_{50} for the TRPC4 homomer is 2385 nM**.** These results demonstrate strong selectivity for the heteromeric complex over the homomeric channel.

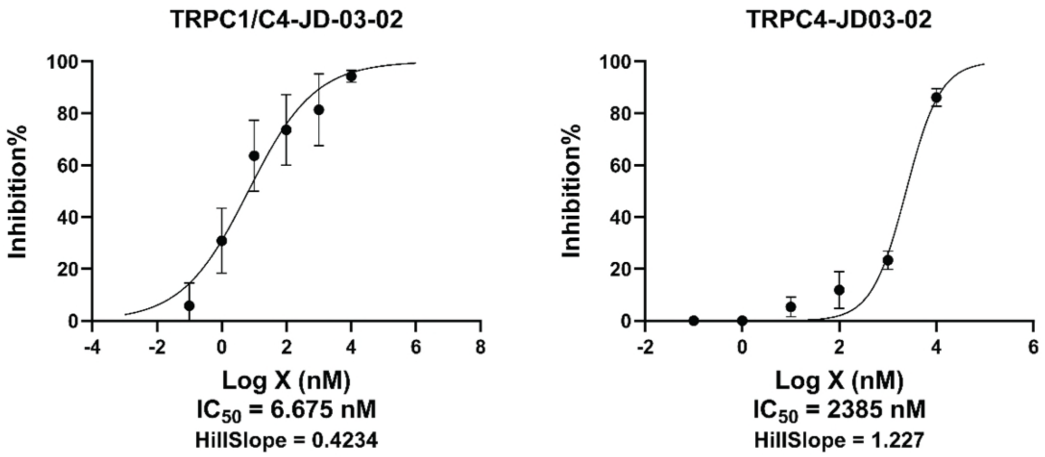

6. Please cite the recent Phase II clinical trial results for BI 1358894 (Shelton et al., Journal of Clinical Psychiatry, 2025; PMID: 40900110) to provide an updated reference.

Response: We thank the reviewer for this suggestion. The Phase II clinical trial results for BI 1358894 (Shelton et al., *Journal of Clinical Psychiatry*, 2025; PMID: 40900110) have now been cited in the revised manuscript.

7. It would be nice for the authors to provide pdb and maps during revision for quality assessment.

Response: We have now deposited the atomic coordinates and cryo-EM maps. The structure is available under PDB ID: 9K4I, and the corresponding maps have been uploaded to Figshare (DOI: 10.6084/m9.figshare.30171676)

Reviewer #2 (Remarks to the Author):

This study presents cryo-electron microscopy (cryo-EM) analysis of the TRPC1/5 heteromer, resolving its high-resolution structure and elucidating unique ligand-binding and functional regulatory mechanisms. Based on these structural findings, the authors developed the small-molecule inhibitor JD03-02, demonstrating its efficacy in animal models of depression and anxiety with reduced side effects compared to conventional treatments. While the study provides valuable insights, the current manuscript lacks sufficient novelty and is premature and mechanistic depth for publication in a high-impact journal. The following major revisions are required to strengthen the work:

To Reviewer #2:

We thank the reviewer for the positive assessment of our work and for the constructive and insightful comments. We appreciate the opportunity to revise the manuscript. The novelty of our study lies in the fact that, to our knowledge, no previous work has leveraged a TRP channel heteromeric structure to guide the development of heteromer-selective therapeutics. Given that heteromerization is widespread among ion channels and that most existing drugs lack such selectivity and therefore cause side effects, we believe our study may provide a general strategy for heteromer-targeted drug discovery. Following the reviewer's suggestions, we have made the revisions detailed below.

1) Structural Limitations of Cryo-EM Data

The current cryo-EM analysis is limited to the ligand-free conformation of TRPC1/5. To fully understand the functional dynamics, structural data on activated or ligand-bound states are essential. The authors should explicitly acknowledge this limitation in the Discussion and propose future experimental directions (e.g., cryo-EM of agonist-bound states or functional assays).

Response: We thank the reviewer for this important suggestion. We have now explicitly acknowledged this limitation in the Discussion. Although we have made multiple attempts to resolve the structure of TRPC1/5 in complex with JD03-02, the transmembrane region did not reach sufficient resolution to reliably model the small

molecule. We therefore state that future efforts will focus on determining agonist- and antagonist-bound TRPC1/5 structures, in combination with functional assays, to further elucidate the molecular mechanisms of channel modulation. In the Discussion section, we have added the following content: "A current limitation is the absence of TRPC1/5 structures in complex with small-molecule modulators. Future studies aimed at solving ligand-bound TRPC1/5 complexes, together with functional validation, will be essential for elucidating the molecular mechanisms of heteromer-selective modulation and for advancing structure-based drug design targeting TRPC heteromeric channels." (Page 17, lines 489-493).

2) Mechanistic Clarification in Behavioral Studies

The differences between JD03-02 and classical antidepressants (e.g., fluoxetine) in CUMS, FST, and SPT experiments require deeper mechanistic explanation.

Response: We thank the reviewer for this comment and have expanded the Discussion to clarify the mechanistic basis underlying the distinct behavioral profiles of JD03-02 and fluoxetine.

In brief, JD03-02 and fluoxetine act through fundamentally different mechanisms. Fluoxetine, as a selective serotonin reuptake inhibitor (SSRI), relies on gradual adaptive changes within monoaminergic systems and typically requires chronic administration to achieve antidepressant efficacy, consistent with our observation that a single dose of fluoxetine does not significantly improve sucrose preference in the CUMS model (Fig. 6b). In contrast, JD03-02 is a highly selective TRPC1/5 heteromer inhibitor (IC_{50} = 0.2 nM; >10,000-fold selectivity over TRPC5 homomers). TRPC1/5 channels are enriched in emotion-related brain regions such as the amygdala and hippocampus (Supplementary Fig. 10), where they directly regulate neuronal excitability and Ca^{2+} homeostasis. Accordingly, acute JD03-02 administration is sufficient to rapidly reverse anhedonia and behavioral despair in CUMS mice (Fig. 6b, d).

Importantly, JD03-02 also exhibits a more favorable side-effect profile. In contrast to non-selective TRPC inhibitors (e.g., HC-070), which induce weight gain, hyperphagia,

and hyperlocomotion through inhibition of TRPC5 homomers, JD03-02 shows no such effects, consistent with its heteromer-specific binding pocket revealed by our structural analysis (Fig. 5a, b).

Together, these results indicate that selective inhibition of TRPC1/5 enables rapid antidepressant efficacy with reduced side effects, distinguishing JD03-02 from classical monoaminergic antidepressants. These points have now been incorporated into the Discussion (Page 15-16, lines 435-468).

3) The Discussion should address potential factors such as:

Target specificity (TRPC1/5 vs. monoaminergic systems)

Pharmacokinetic properties (e.g., half-life, distribution)

Blood-brain barrier permeability (quantitative comparisons if available)

Response: We thank the reviewer for these detailed suggestions. We have now expanded both the Results and Discussion accordingly. RNAscope analyses demonstrate high co-expression of TRPC1 and TRPC5 in the amygdala, a key region for negative emotional regulation (Supplementary Fig. 10e). Ex vivo brain-slice recordings further show that JD03-02 selectively suppresses TRPC1/5-mediated neuronal hyperexcitability induced by CCK-4 (Supplementary Fig. 10a-d).

In addition, we have added comprehensive pharmacokinetic data (Tables 2–3), demonstrating that JD03-02 has favorable oral bioavailability (F = 49.8%) and good brain penetration (brain/plasma ratio = 0.67). These data support the conclusion that JD03-02 effectively engages central TRPC1/5 targets in vivo. The corresponding discussion has been added (Page 16, lines 450-468).

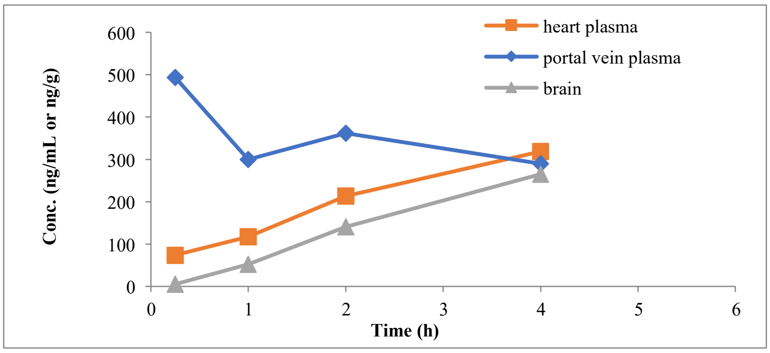

Pharmacokinetics curve of JD03-02 after 10mg/kg p.o administration in mouse

Table 2 Pharmacokinetics parameters of JD03-02 after p.o administration in mouse

Compound	Matrix	T_{max} (h)	C_{max} (ng/mL or ng/g)	AUC_{last} (h*ng/mL or h*ng/g)	AUC_{last} brain/AUC_{last} heart plasma	AUC_{last} heart plasma/AUC_{last} portal vein plasma	C_{last} brain/C_{max} heart plasma
JD03-02	heart plasma	4.00	318	778	0.67	0.58	0.83
	portal vein plasma	0.25	493	1332			
	brain	4.00	265	524			

Table 3 Pharmacokinetics parameters of JD03-02 after 10mg/kg p.o and 2mg/kg i.v. administration in mouse

Administration	Animal No.	$T_{1/2}$ (h)	T_{max} (h)	C_{max} (ng/mL)	AUC_{last} (h*ng/mL)	AUC_{INF_obs} (h*ng/mL)	CL_obs (mL/min/kg)	MRT_{INF_obs} (h)	Vss_obs (mL/kg)	F (%)
p.o.	1	2.86	1.00	256	1294	1299	-	4.73	-	49.8
	2	3.35	1.00	205	1532	1549	-	6.50	-	
	3	2.48	1.00	143	746	800	-	4.58	-	
	**Mean**	**2.90**	**1.00**	**201**	**1191**	**1216**	-	**5.27**	-	
	**SD**	**0.44**	**0.00**	**57**	**403**	**381**	-	**1.06**	-	
i.v.	4	3.52	-	-	385	469	71.0	4.455	18984	
	5	2.06	-	-	416	447	74.6	2.895	12955	
	6	3.69	-	-	634	640	52.1	4.460	13949	
	**Mean**	**3.09**	-	-	**478**	**519**	**65.9**	**3.937**	**15296**	
	**SD**	**0.90**	-	-	**135**	**105**	**12.1**	**0.902**	**3233**	

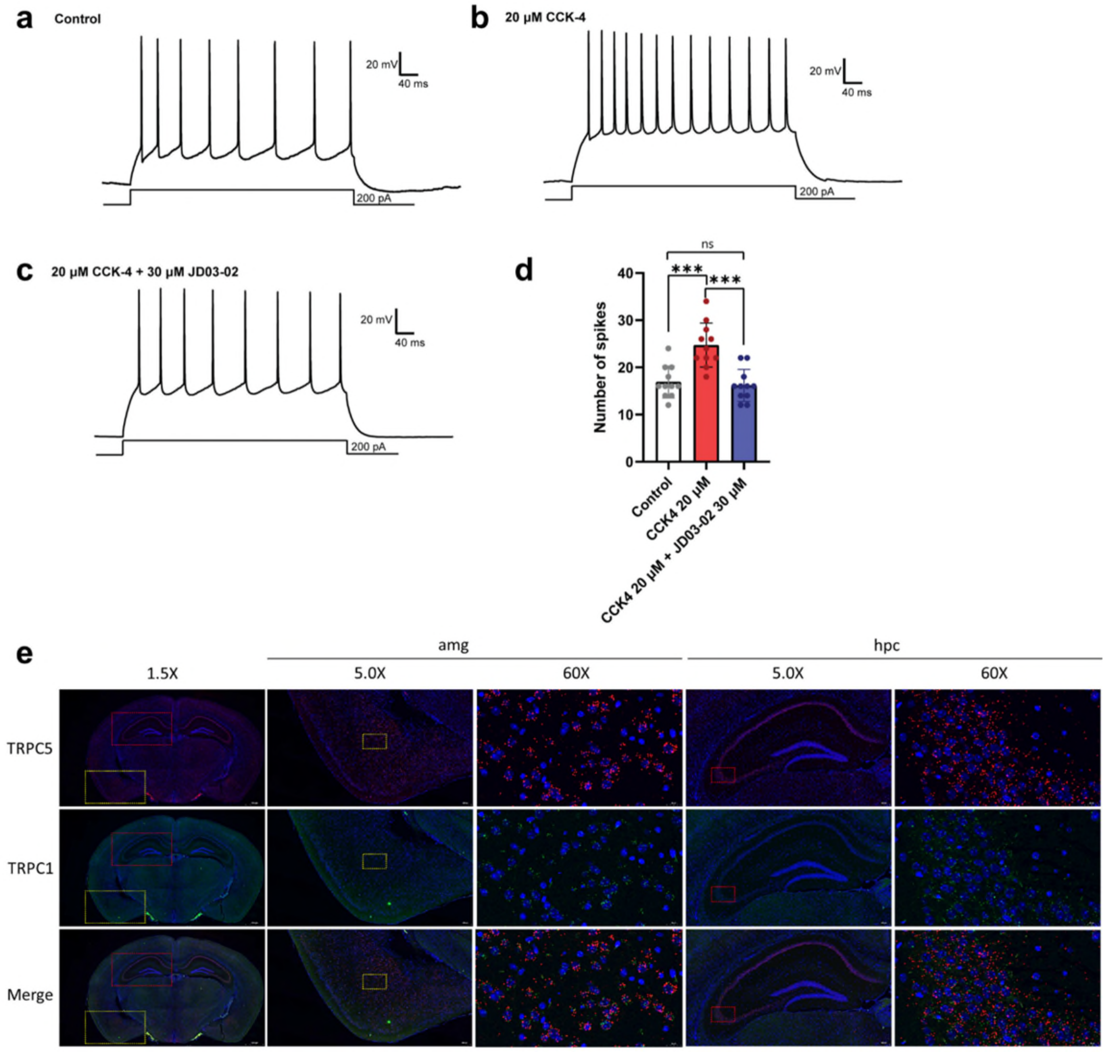

4) Strengthening Mechanistic Interpretations

While a novel binding pocket at the TRPC1-TRPC5 interface is identified, the proposed "negative regulatory" role of TRPC1 in channel gating and calcium permeability remains speculative. Additional experiments are needed:

Electrophysiological recordings of TRPC1/5 vs. TRPC5 homomers

Molecular dynamics simulations of gating mechanisms

Calcium imaging studies with TRPC1 knockdown/knockout controls

Response: We appreciate this thoughtful comment. Although previous studies have suggested that TRPC1 negatively regulates Ca^{2+} permeability in heteromeric channels (e.g., Storch et al., *J Bio Chem*, 2012. PMID: 22157757; Kim et al., *Pflugers Arch*, 2019. PMID: 31222490), we agree that our current data are insufficient to definitively establish this role in channel gating.

We therefore performed additional electrophysiological recordings comparing TRPC1/5 heteromers and TRPC5 homomers. Mutation of Ca^{2+}-binding residues in the three TRPC5 subunits significantly reduced current amplitudes in the heteromer ($p < 0.01$), whereas analogous mutations in the single TRPC1 subunit had no significant effect ($p > 0.05$; Fig. 5e). These results suggest that the absence of a canonical Ca^{2+}-binding site in TRPC1 may alter Ca^{2+} coordination and selectivity within the heteromeric channel.

To maintain rigor, we have revised the text to avoid overinterpretation and now state that TRPC1 may modulate Ca^{2+} binding and selectivity, rather than asserting a definitive negative regulatory role. In the Results section, we have added the following content: "Our structural and functional data indicate that, unlike TRPC5, the TRPC1 subunit lacks a functional, acidic Ca^{2+}-binding pocket, precluding Ca^{2+} coordination at this site. This structural divergence likely alters Ca^{2+} sensing and may contribute to differences in ion selectivity and gating behavior between TRPC1-containing heteromers and TRPC5 homomeric channels." (Page 10, lines 288-292)

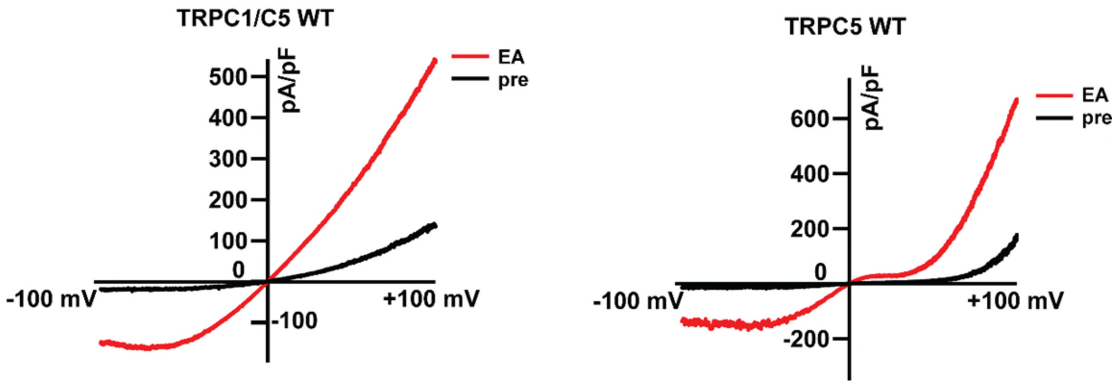

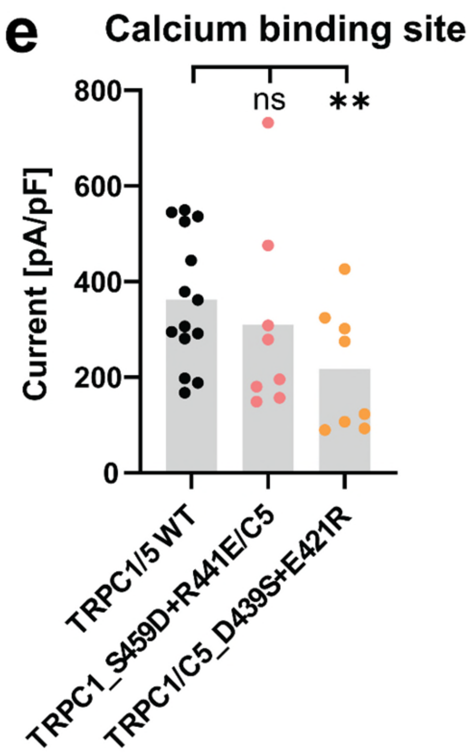

Figure 5e.

5) Agonist Selection Rationale

The use of Englerin A as an agonist in TRPC1/5 stoichiometry studies is problematic, given its established preference for TRPC4/5. The authors must:

Justify this experimental choice with supporting literature

Include controls to rule out TRPC5 homomer contributions (e.g., TRPC1-specific knockdowns)

Consider alternative agonists (e.g., OAG) for validation

Response: We thank the reviewer for this important point. EA was chosen because it is a well-established agonist not only for TRPC4/5 homomers but also for TRPC1/4/5 heteromers (Akbulut et al., *Angew Chem Int Ed Engl*, 2015. PMID: 25707820; Rubaiy et al., *Br J Pharmacol*, 2018. PMID: 29247460). In contrast, OAG primarily activates TRPC3/6 and is therefore less suitable for probing TRPC1/5 function.

To exclude contributions from TRPC5 homomers, we performed additional patch-clamp experiments using the TRPC5-selective agonist AM237, which robustly activates TRPC5 homomers but not TRPC1/5 heteromers. In cells co-transfected with

TRPC1 and TRPC5 at ratios of 3:1 or 6:1, AM237 failed to elicit detectable currents, whereas subsequent application of EA induced characteristic outwardly rectifying TRPC1/5 currents. These results confirm that the recorded currents predominantly arise from TRPC1/5 heteromers. To further strengthen our conclusion, we performed FRET-based subunit interaction experiments (Figure 1c and Supplementary Fig. 2). These experiments show robust FRET between fluorescently tagged TRPC1 and TRPC5, with efficiencies comparable to those observed for TRPC5 homomers. In contrast, TRPC1–TRPC1 FRET is minimal in the presence of TRPC5. Together, these results provide independent and complementary evidence that the dominant heteromeric complex consists of one TRPC1 subunit and three TRPC5 subunits, consistent across both in vitro and cellular assays.

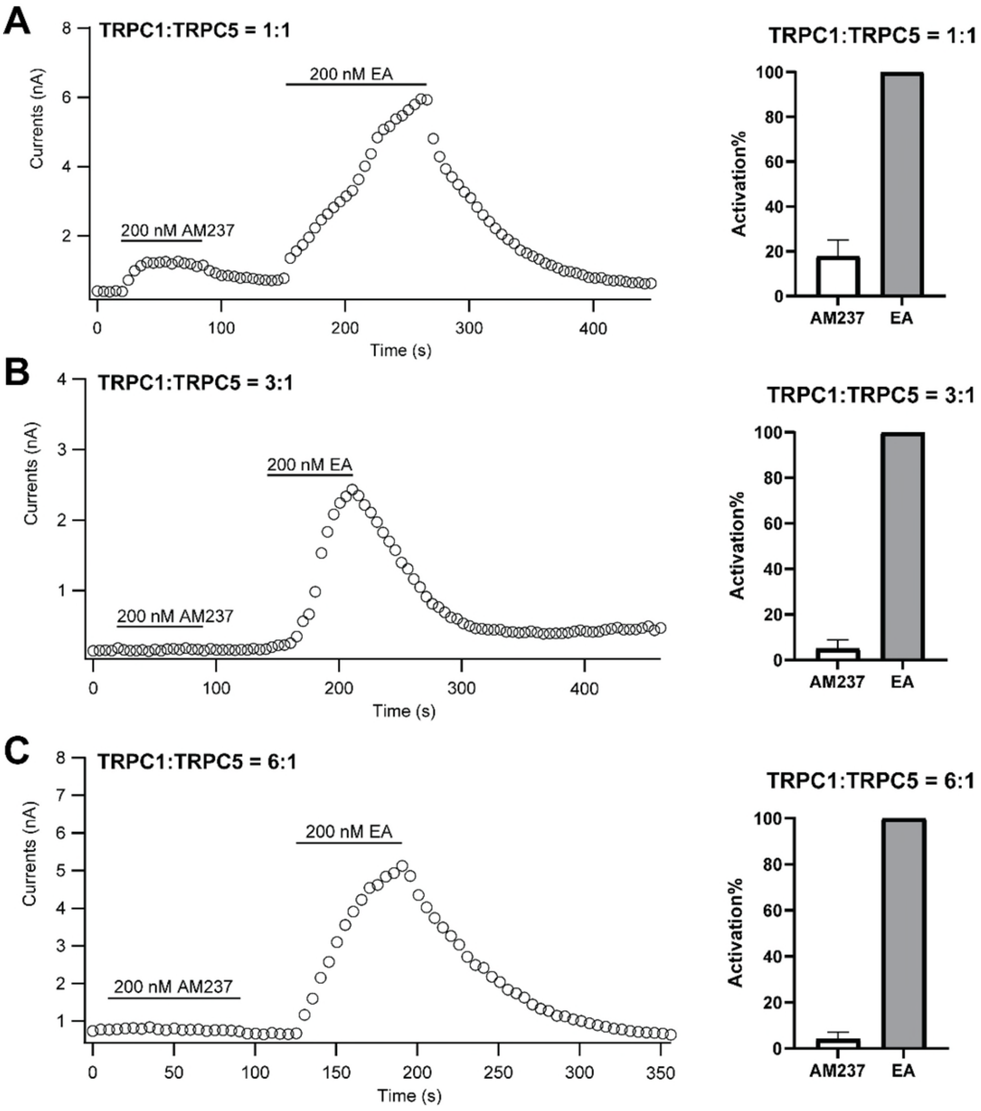

Figure 1c

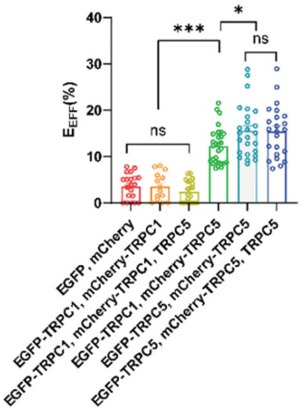

Supplementary Fig.2

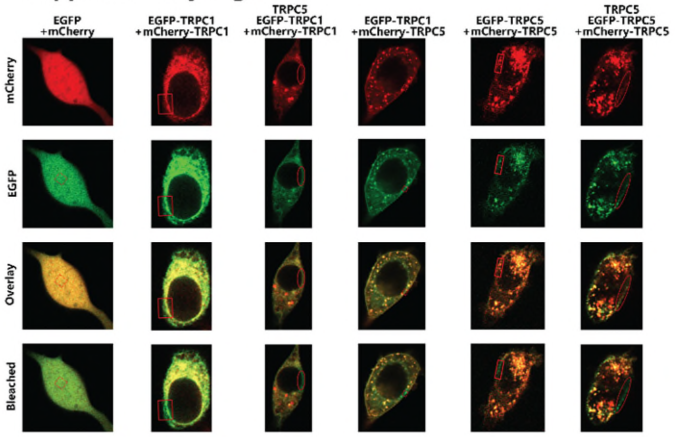

6) Technical Corrections

Standardize IC_{50} formatting (e.g., "IC_{50} = 6.7 nM")

Clarify the relevance of Figure 10 (H08's TRPC1/4 selectivity) to the TRPC1/5 focus

Provide high-resolution versions of all figures

Response: We have standardized IC_{50} formatting throughout the manuscript, clarified the relevance of H08 selectivity for TRPC1/4 in relation to TRPC1/5 (given the high transmembrane homology between TRPC4 and TRPC5), and provided high-resolution versions of all figures.

7) Expanded Discussion Requirements

The Discussion must now include:

Comparative analysis of JD03-02's pharmacokinetics vs. reference compounds

Toxicity profile discussion (e.g., off-target effects)

Clinical translation potential (e.g., dosing considerations, therapeutic window)

Response: We thank the reviewer for this constructive suggestion. In response, we have expanded the Discussion to include a comparative analysis of the pharmacokinetic properties, selectivity/toxicity profile, and clinical translation potential of JD03-02.

Specifically, we have performed comprehensive pharmacokinetic studies of JD03-02 following both oral (p.o.) and intravenous (i.v.) administration. As summarized in Table 2, JD03-02 exhibits favorable oral bioavailability (F = 49.8%), which represents a substantial improvement over previously reported TRPC inhibitors, such as HC-070 (F ≈ 13%) (Just et al., *PloS One*, 2018. PMID: 29385160). In addition, JD03-02 demonstrates efficient central nervous system exposure, with a brain-to-plasma ratio (B/P) of 0.67, supporting its suitability for targeting CNS TRPC1/5 channels.

To address potential toxicity and off-target effects, we systematically evaluated the activity of JD03-02 against other closely related TRPC family members. JD03-02 showed minimal inhibitory activity toward TRPC3, TRPC6, and TRPC7, with IC_{50} values exceeding 10 μM, indicating a high degree of target selectivity. Together with the absence of detectable effects on body weight and food intake during repeated dosing in vivo, these data suggest a favorable preliminary safety and tolerability profile.

Regarding clinical translation, the combination of high oral bioavailability, robust brain penetration, nanomolar potency, and pronounced selectivity for TRPC1-containing heteromers suggests that JD03-02 may achieve therapeutic efficacy at relatively low systemic exposures, potentially affording a wide therapeutic window. These properties, together with its rapid onset and sustained antidepressant efficacy in the CUMS model, support the translational potential of JD03-02 as a lead compound for further preclinical development. The Discussion section of the manuscript has been revised accordingly to incorporate these points (Page 16, Lines 450-468).

"JD03-02 exhibits favorable drug-like properties, including high oral bioavailability (49.8%) and efficient brain penetration (B/P = 0.67), representing a clear improvement over previously reported TRPC inhibitors. The compound shows high selectivity for TRPC1-containing heteromers, with minimal activity against other TRPC family members (IC_{50} > 10 μM), and demonstrates good tolerability upon repeated dosing. Together with its nanomolar potency and robust antidepressant efficacy in the CUMS model, these features support a potentially wide therapeutic window and position JD03-02 as a promising lead for further preclinical development.".

Table 2 Pharmacokinetics parameters of JD03-02 after p.o administration in mouse

**Compound**	**Matrix**	**T_{max} (h)**	**C_{max} (ng/mL or ng/g)**	**AUC_{last} (h*ng/mL or h*ng/g)**	**AUC_{last} brain/AUC_{last} heart plasma**	**AUC_{last} heart plasma/AUC_{last} portal vein plasma**	**C_{last} brain/C_{max} heart plasma**
JD03-02	heart plasma	4.00	318	778	0.67	0.58	0.83
	portal vein plasma	0.25	493	1332			
	brain	4.00	265	524			

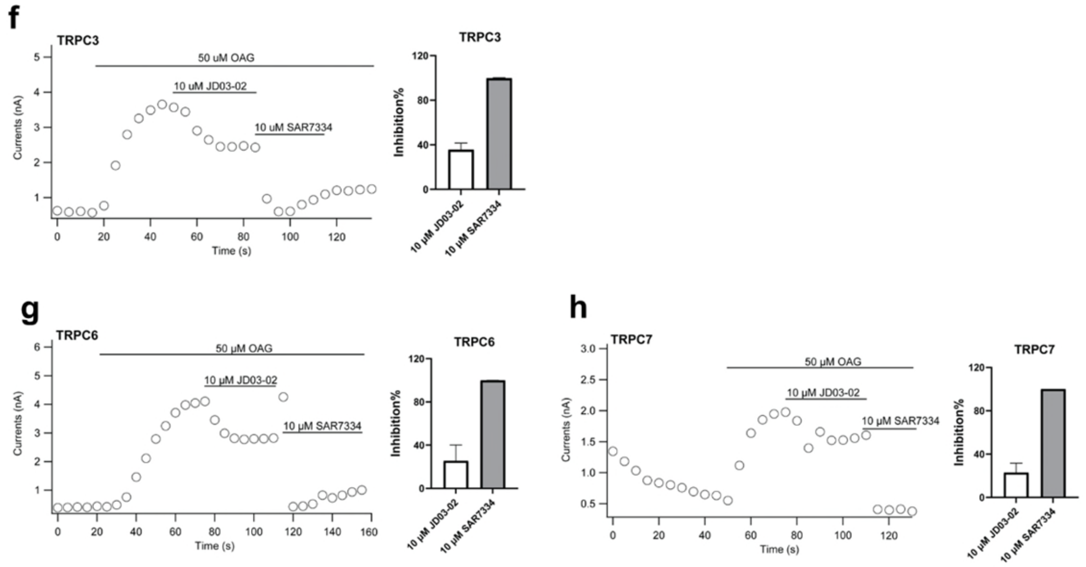

Supplementary Figure 9f-h.

8) Selectivity Data Gap

While JD03-02 shows potent inhibition of TRPC1/5 and TRPC5, selectivity against other TRPC subtypes (e.g., TRPC3, TRPC6) must be demonstrated experimentally. Include:

Dose-response curves for at least 3 additional TRPC subtypes

Selectivity index calculations

Response: We performed additional patch-clamp experiments on TRPC3, TRPC6, and TRPC7. At 10 μM, JD03-02 produced only modest inhibition (~30–40%) of these channels, demonstrating high selectivity for TRPC1/5 heteromers (Supplementary Fig. 9f–h).

Supplementary Figure 9f-h.

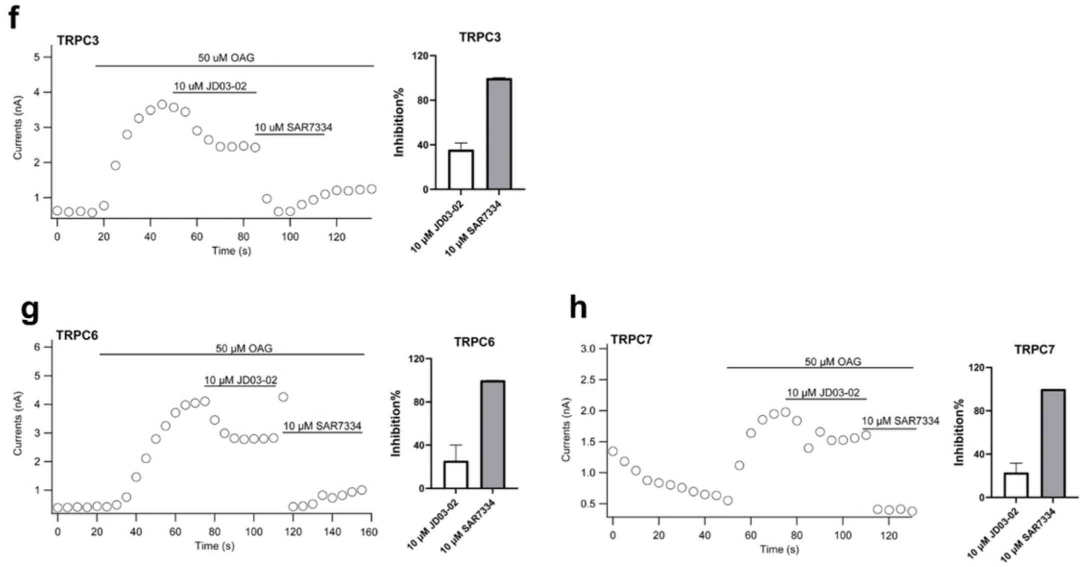

9) Figure Improvements

Simplify Extended Data figures with schematic summaries

Add a comparative diagram of TRPC1/5 vs. TRPC5 structural/functional features

Response: We have simplified the Extended Data figures (Supplementary figures) and added a comparative schematic highlighting key structural and functional differences between TRPC1/5 heteromers and TRPC5 homomers.

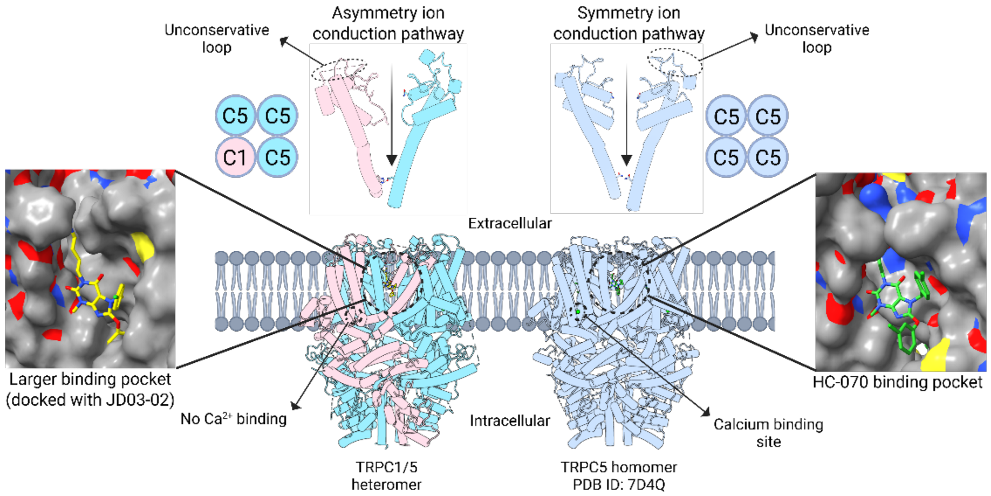

Reviewer #3 (Remarks to the Author):

Based on high-resolution structural information about TRPC1/5 heterotetramers obtained at 2.8Å resolution, the authors designed a high-affinity antagonist, named JD03-02, that was highly selective in blocking TRPC1/5 complexes over TRPC5 homomeric channels. Specifically, the newly synthesized compound JD03-02 exhibited >10,000-fold selectivity for TRPC1/5 over TRPC5 homomers, thus allowing selective blockade of TRPC1/5 heteromers. At the functional level, the authors found that systemic administration of JD03-02 produced anxiolytic and antidepressant effects in mice. This is an interesting study providing a good example of how understanding the ion channels' molecular architecture could be used for structure-based drug design. More specifically, the reported findings suggest that TRPC channels with different subunit composition could differentially contribute to behavioral mechanisms. For the most part, the studies are well designed and results are interesting. In my view, however, there are certain issues with the present work which should be resolved before it could be recommended for publication.

To Reviewer #3

We sincerely thank Reviewer #3 for the thoughtful and constructive evaluation of our manuscript, and for recognizing the conceptual advance of leveraging high-resolution structural information of TRPC1/5 heteromers for structure-based drug design. We particularly appreciate the reviewer's comments on the behavioral and mechanistic aspects of the study, which have helped us substantially improve the clarity, rigor, and interpretability of the manuscript. Below, we provide a detailed point-by-point response to each concern.

1. The description of behavioral studies is somewhat confusing. It would be helpful if the authors could include a diagram showing at what time points relative to the behavioral testing the pharmacological interventions were performed. Although it's not immediately clear which experimental groups were compared. It should be explicitly stated in the manuscript and statistical results should be fully reported, including exact P and t values.

Response: We thank the reviewer for this valuable suggestion. To clarify the experimental timeline, we have now added a schematic diagram illustrating the time points of pharmacological interventions relative to behavioral testing in the relevant figures. Furthermore, we have revised the figure legends to explicitly state the experimental groups being compared. As recommended, we have also provided the exact P and t values for all statistical comparisons within the main text describing these results.

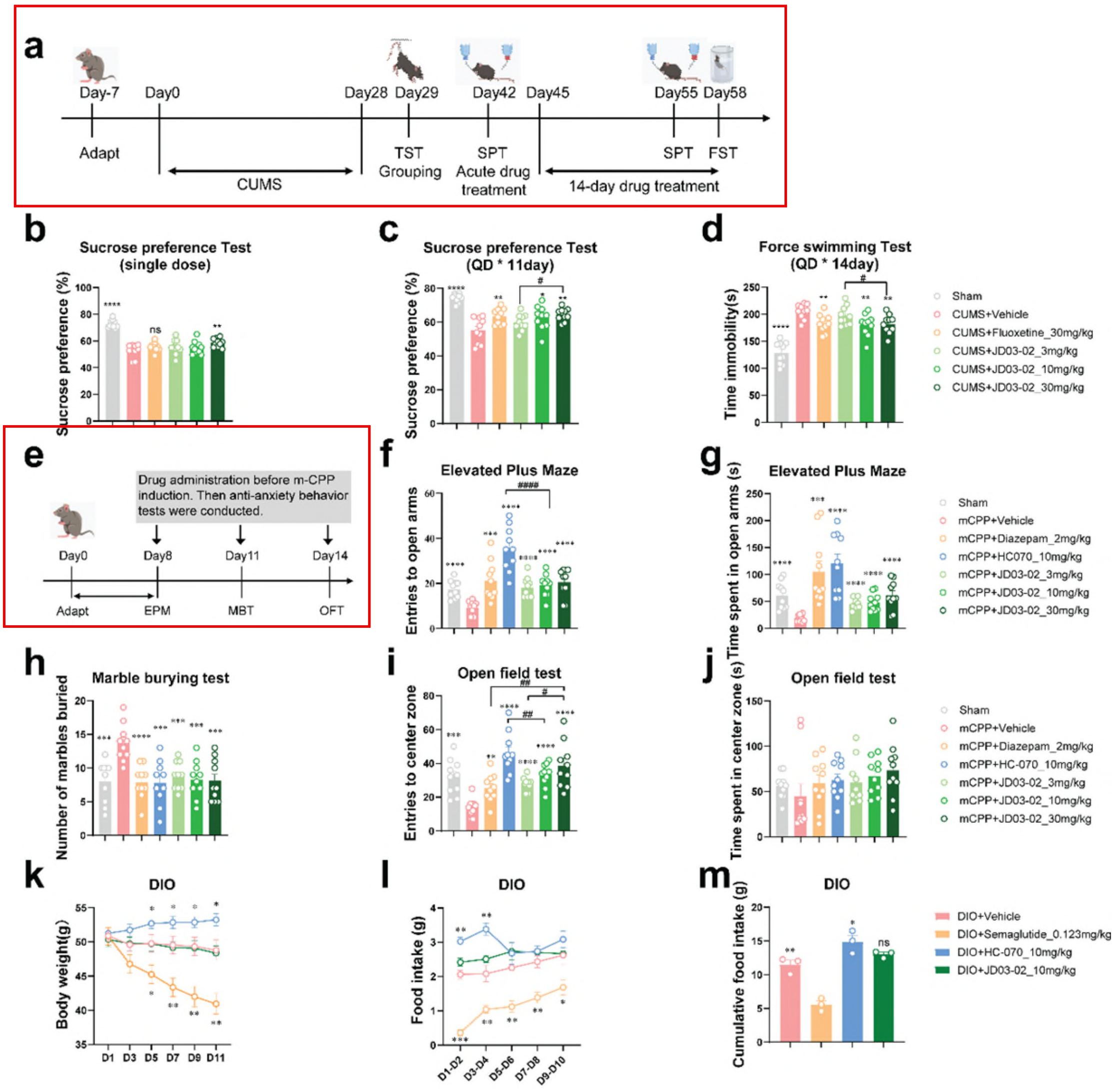

2. The sample sizes were relatively small, so t-test might be not appropriate if the data points are not normally distributed. Was the normalcy of the fata points distribution evaluated?

Response: We appreciate the reviewer's important point regarding statistical methodology. For all datasets in the animal experiments, we formally assessed the normality of distribution using the Shapiro-Wilk test in GraphPad Prism 9. The results confirmed that the majority of our data sets met the assumption of normality, justifying the use of parametric t-tests. For the few groups where data were not normally distributed, we employed the non-parametric Mann-Whitney U test. Detailed results of the normality tests and the corresponding P values are provided in the Supplementary Information.

Efficacy data in the CUMS-induced depression model.

		SPT After Acute Administration in the CUMS Model					
Origional data	Animal no.	Control	CUMS+Vehicle	CUMS+Fluoxetine_30mg/kg	CUMS+JD03-02_3mg/kg	CUMS+JD03-02_10mg/kg	CUMS+JD03-02_30mg/kg
	1	74.45	54.46	54.05	60.34	49.54	59.23
	2	69.87	43.88	55.79	55.56	55.29	57.76
	3	74.21	52.43	56.6	54.55	57.14	55.56
	4	69.18	56.78	56.48	53.7	49.6	62.07
	5	70.8	54	58.82	64.6	59.29	55.12
	6	75.89	44.29	49.04	55.24	55.24	61.61
	7	70.63	56.19	55.46	54.95	51.72	59.52
	8	71.34	57.14	55.36	45.19	52.17	63.48
	9	78.42	56.25	53.92	60.17	65.08	57.78
	10	70.86	52.83	61.4	50	53.91	53.85
Test for normal distribution(Shapiro-Wilk test)	W	0.8936	0.7842	0.9394	0.9586	0.9223	0.9626
	P value	0.186	0.0093	0.5462	0.7698	0.3762	0.8146
	Passed normality test	Yes	No	Yes	Yes	Yes	Yes

P value	Control	CUMS+Vehicle	CUMS+Fluoxetine_30mg/kg	CUMS+JD03-02_3mg/kg	CUMS+JD03-02_10mg/kg	CUMS+JD03-02_30mg/kg
Control		<0.0001				
CUMS+Vehicle		/				
CUMS+Fluoxetine_30mg/kg		0.1399				
CUMS+JD03-02_3mg/kg		0.1965				
CUMS+JD03-02_10mg/kg		0.4061				
CUMS+JD03-02_30mg/kg		0.0026	0.0290			

T value	Control	CUMS+Vehicle	CUMS+Fluoxetine_30mg/kg	CUMS+JD03-02_3mg/kg	CUMS+JD03-02_10mg/kg	CUMS+JD03-02_30mg/kg
Control		not applicable				
CUMS+Vehicle		not applicable				
CUMS+Fluoxetine_30mg/kg		not applicable				
CUMS+JD03-02_3mg/kg		not applicable				
CUMS+JD03-02_10mg/kg		not applicable				
CUMS+JD03-02_30mg/kg		not applic	2.0240			

		SPT After 11-Day Administration in the CUMS Model					
Origional data	Animal no.	Control	CUMS+Vehicle	CUMS+Fluoxetine_30mg/kg	CUMS+JD03-02_3mg/kg	CUMS+JD03-02_10mg/kg	CUMS+JD03-02_30mg/kg
	1	77.18	52.94	66.13	60	64.93	62.5
	2	76.81	46.67	67.48	51.91	58.04	70.31
	3	75.81	62.75	57.8	67.97	48.33	63.16
	4	75.51	57.8	58.04	63.77	64.04	58.4
	5	70.8	63.33	67.86	57.69	67.59	59.5
	6	73.15	53.92	65.22	58.18	72.93	67.5
	7	74.34	44.23	70.4	63.04	57.36	66.21
	8	74.6	60.42	59.7	54.84	60.48	66.4
	9	69.4	63.64	63.03	57.97	69.03	66.93
	10	75.34	45.95	58.73	63.57	64	63.19
Test for normal distribution(Shapiro-Wilk test)	W	0.904	0.8893	0.9091	0.9734	0.9623	0.9575
	P value	0.2421	0.1664	0.2749	0.9207	0.8117	0.7574
	Passed normality test (alpha=0.05)?	Yes	Yes	Yes	Yes	Yes	Yes

P value	Control	CUMS+Vehicle	CUMS+Fluoxetine_30mg/kg	CUMS+JD03-02_3mg/kg	CUMS+JD03-02_10mg/kg	CUMS+JD03-02_30mg/kg
Control		<0.0001				
CUMS+Vehicle						
CUMS+Fluoxetine_30mg/kg		0.0042				
CUMS+JD03-02_3mg/kg		0.0557	0.0542			
CUMS+JD03-02_10mg/kg		0.0165	0.3874	0.1562		
CUMS+JD03-02_30mg/kg		0.0014	0.3056	0.0149		

T value	Control	CUMS+Vehicle	CUMS+Fluoxetine_30mg/kg	CUMS+JD03-02_3mg/kg	CUMS+JD03-02_10mg/kg	CUMS+JD03-02_30mg/kg
Control		7.5960				
CUMS+Vehicle						
CUMS+Fluoxetine_30mg/kg		2.9550				
CUMS+JD03-02_3mg/kg		1.6740	1.6890			
CUMS+JD03-02_10mg/kg		2.3080	0.2893	1.04		
CUMS+JD03-02_30mg/kg		3.4710	0.5175	2.361		

		FST After 14-Day Administration in the CUMS Model					
Origional data	Animal no.	Control	CUMS+Vehicle	CUMS+Fluoxetine_30mg/kg	CUMS+JD03-02_3mg/kg	CUMS+JD03-02_10mg/kg	CUMS+JD03-02_30mg/kg
	1	156.4	215.9	212.2	199.6	168.6	208.3
	2	146.7	196.9	190.6	177	201.9	199.8
	3	120.5	188.3	181.5	218.3	189.6	180.6
	4	105.4	205.7	173.7	210.4	186.1	172.5
	5	108.4	213.6	161.4	179.9	207.8	189.2
	6	98.2	179.1	177.5	229.5	138	171.7
	7	140	220.5	182.5	195.8	183.7	149.8
	8	145.3	218.8	197.5	189.2	161	189.4
	9	144	207	193.1	188.7	179	165.6
	10	119.1	205.6	158.4	181.5	186.2	190.1
Test for normal distribution(Shapiro-Wilk test)	W	0.9142	0.9204	0.9766	0.9258	0.9429	0.9758
	P value	0.3111	0.3604	0.9447	0.408	0.5856	0.9391
	Passed normality test (alpha=0.05)?	Yes	Yes	Yes	Yes	Yes	Yes

P value	Control	CUMS+Vehicle	CUMS+Fluoxetine_30mg/kg	CUMS+JD03-02_3mg/kg	CUMS+JD03-02_10mg/kg	CUMS+JD03-02_30mg/kg
Control		<0.0001				
CUMS+Vehicle						
CUMS+Fluoxetine_30mg/kg		0.0019				
CUMS+JD03-02_3mg/kg		0.129				
CUMS+JD03-02_10mg/kg		0.0023	0.3757			
CUMS+JD03-02_30mg/kg		0.0017	0.4406	0.0325		

T value	Control	CUMS+Vehicle	CUMS+Fluoxetine_30mg/kg	CUMS+JD03-02_3mg/kg	CUMS+JD03-02_10mg/kg	CUMS+JD03-02_30mg/kg
Control		9.8930				
CUMS+Vehicle						
CUMS+Fluoxetine_30mg/kg		3.3220				
CUMS+JD03-02_3mg/kg		1.1650				
CUMS+JD03-02_10mg/kg		3.2410	0.3218			
CUMS+JD03-02_30mg/kg		3.3800	0.1515	1.966		

Efficacy data in the mCPP-induced anxiety model.

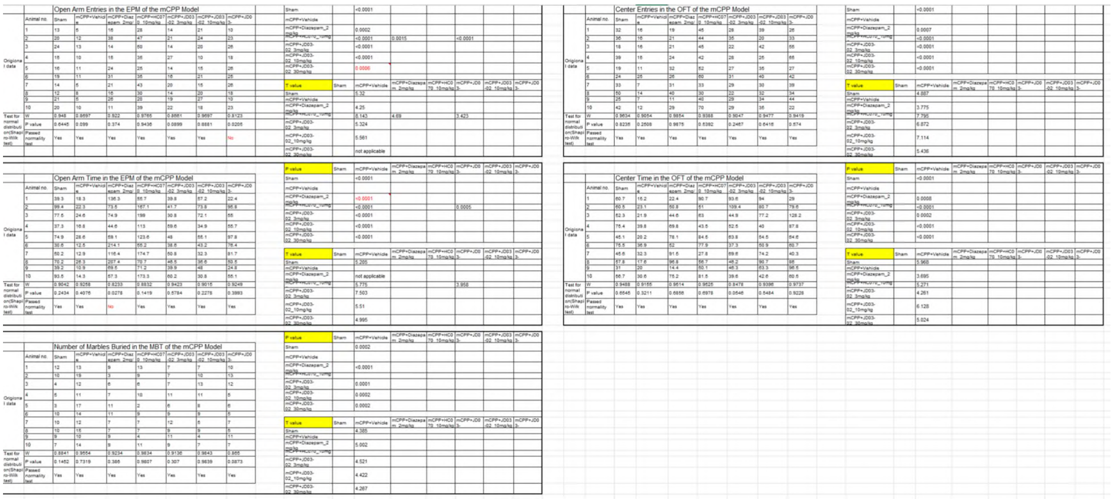

Efficacy data in the diet-induced obesity (DIO) model.

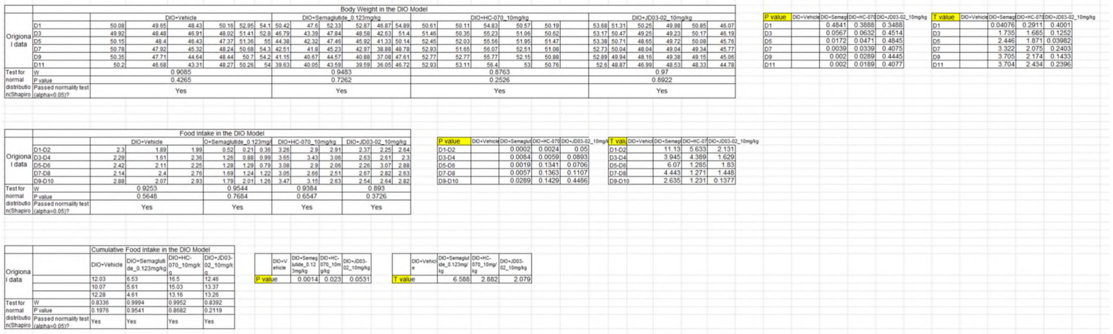

3. Another issue is that behavioral results are not fully reported. For the open field test both the number of entries to the center and time spent in the center should be reported. In the EPM test, both number of open arm entries and time spent in open arms should be included.

Response: We thank the reviewer for highlighting this oversight. We have now supplemented the figures to fully report all standard behavioral parameters. Specifically, we have added the *time spent in the center* for the Open Field Test (OFT) and the *time spent in the open arms* for the Elevated Plus Maze (EPM) test, alongside the originally reported entries.

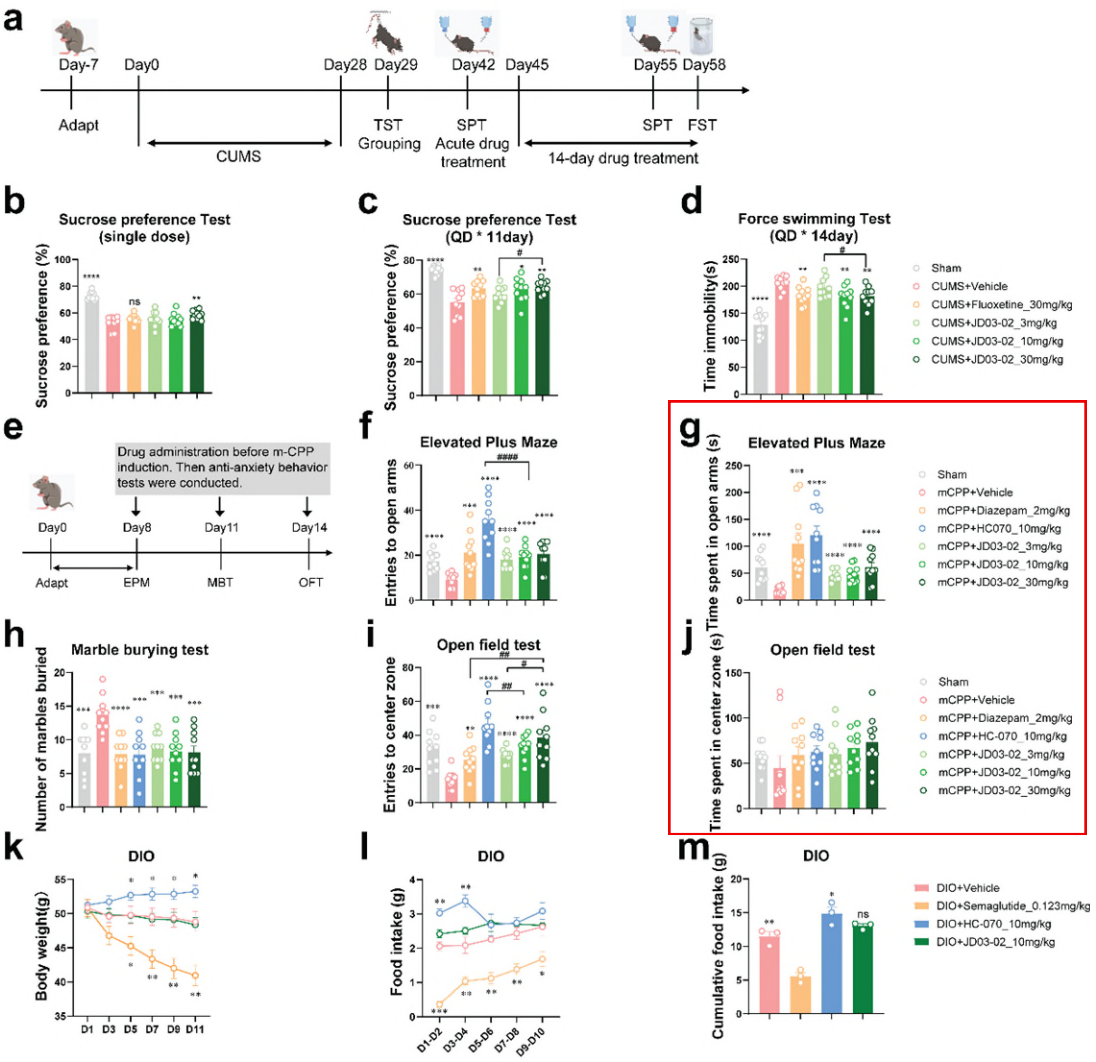

4. It is implied in the manuscript that continuous (lasting for 11-14 days) daily treatment with orally-administered JD03-02 was more efficient in reducing CUMS-induced depressive-like behaviors in mice. However, there is no any discussion in the manuscript how this might work. Was the difference between behavioral effects of a single dose of JD03-02 statistically different from the effects of continuous drug delivery? How long-lasting was the blocking effect of JD03-02 on TRPC1/5 heteromers? What is actually evidence here that behavioral improvements following JD03-02 administration were exclusively due to the blockade of TRPC1/5 channels?

Response:

We thank the reviewer for raising these critical points regarding the dosing regimen and mechanism of action.

- **Comparison of Dosing Regimens:** We have added a direct comparison between single and continuous administration of JD03-02 (now included as a new figure panel). Statistical analysis confirms that the antidepressant effect of JD03-02 is significantly enhanced after 11 days of continuous administration compared to a single dose ($p < 0.01$). This contrasts with fluoxetine, an SSRI which, consistent with its known mechanism requiring chronic adaptation, showed efficacy only after continuous, but not single, administration ($p < 0.001$ for chronic effect).
- **Duration of TRPC1/5 Blockade & PK/PD Relationship:** As an ion channel blocker, the inhibitory effect of JD03-02 on TRPC1/5 is expected to persist as long as its concentration at the target site remains above the effective level. Pharmacokinetic data show that a single 10 mg/kg oral dose of JD03-02 achieves a brain concentration of ~513 nM at 4 hours, which is substantially higher than its IC_{50} (0.2 nM) for TRPC1/5 heteromers, supporting a sustained pharmacodynamic effect aligned with its pharmacokinetic profile.
- **Evidence for TRPC1/5-Specific Action:** We agree that studies in TRPC1 knockout animals would provide the most direct genetic evidence, which remains a goal for future research. However, our current study provides strong pharmacological and anatomical support: 1) *In vitro*, JD03-02 potently and selectively inhibits TRPC1/5 heteromers but not TRPC5 homomers; 2) RNAscope analysis reveals co-expression of TRPC1 and TRPC5 in key limbic regions like the amygdala; 3) *Ex vivo*, JD03-02 specifically reverses CCK-4-induced neuronal hyperexcitability in amygdala slices. Collectively, these lines of evidence allow us to reasonably conclude that the behavioral improvements are mediated primarily via blockade of TRPC1/5 channels.

We have incorporated the following relevant descriptions into our discussion: " JD03-02 shows robust antidepressant and anxiolytic efficacy without these liabilities. These

distinctions likely stem from selective inhibition of TRPC1/5 heteromers while sparing TRPC5 homomers, which are implicated in oxytocinergic regulation of appetite and stress. Our findings support a growing hypothesis that TRPC1/5 heteromers, rather than TRPC5 alone, may serve as the more disease-relevant channel complex in limbic and hypothalamic circuits.

Given the limitations of current antidepressant and anxiolytic therapies—including delayed onset of action, incomplete efficacy, and frequent adverse effects—TRPC1/5-selective modulation represents a mechanistically novel and potentially safer therapeutic strategy. To assess the in vivo antidepressant potential of TRPC1/5 inhibition, we employed the chronic unpredictable mild stress (CUMS) model, a well-established paradigm that recapitulates key behavioral and physiological features of human depression and remains a critical benchmark in preclinical antidepressant development. Within this model, the TRPC1/5-selective inhibitor JD03-02 robustly reversed CUMS-induced depressive-like behaviors across multiple domains. Acute administration of JD03-02 rapidly alleviated anhedonia and behavioral despair, as evidenced by increased sucrose preference and reduced immobility in the forced swim test, whereas a single dose of fluoxetine was ineffective, consistent with the delayed onset of selective serotonin reuptake inhibitors (SSRIs). Upon repeated administration (11–14 days), JD03-02 produced greater and more sustained behavioral improvements than fluoxetine, with no evidence of tolerance.

Importantly, JD03-02 exhibited favorable drug-like properties, including high oral bioavailability (49.8%) and efficient central nervous system exposure, with a brain-to-plasma ratio (B/P) of 0.67 (Table 2). Chronic dosing did not affect body weight or food intake in diet-induced obesity mice, indicating good tolerability and minimal metabolic liability. At the mechanistic level, TRPC1 and TRPC5 are co-expressed in brain regions implicated in mood regulation, including the amygdala and hippocampus (Extended Data Fig. 10E). Consistent with this expression pattern, ex vivo electrophysiological recordings demonstrated that JD03-02 selectively suppresses CCK-4-induced hyperexcitability of lateral amygdala neurons (Supplementary Fig. 10a-d), supporting

a circuit-level mechanism whereby TRPC1/5 inhibition normalizes stress-induced neuronal overactivation. JD03-02 exhibits favorable drug-like properties, including high oral bioavailability (49.8%) and efficient brain penetration (B/P = 0.67), representing a clear improvement over previously reported TRPC inhibitors. The compound shows high selectivity for TRPC1-containing heteromers, with minimal activity against other TRPC family members (IC_{50} > 10 μM), and demonstrates good tolerability upon repeated dosing. Together with its nanomolar potency and robust antidepressant efficacy in the CUMS model, these features support a potentially wide therapeutic window and position JD03-02 as a promising lead for further preclinical development." (Page15-16, lines 429-468)

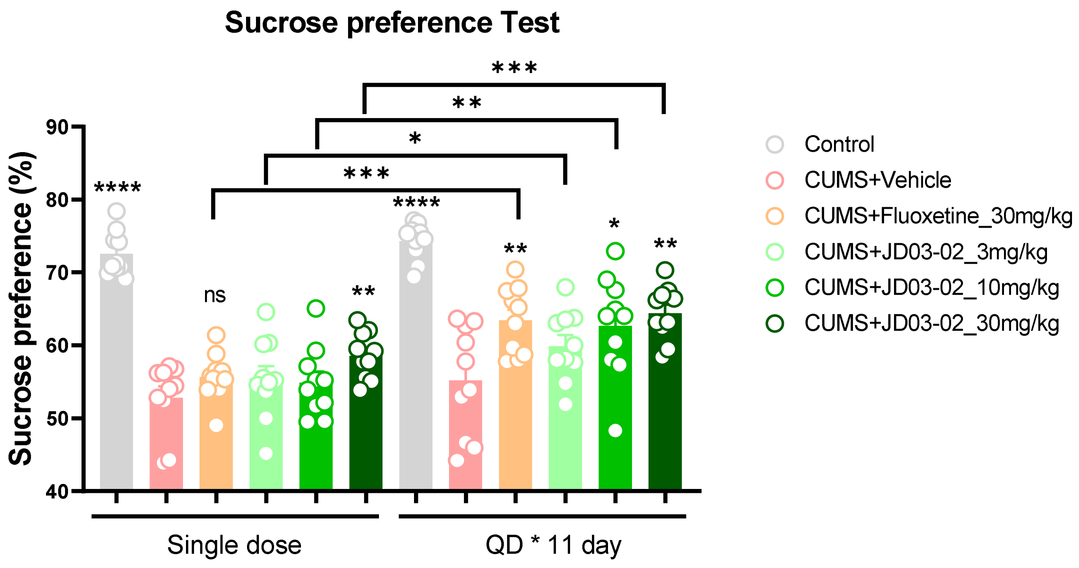

Figure. Comparison of the efficacy between acute and chronic administration of JD03-02 in the CUMS-induced mouse model of depression.

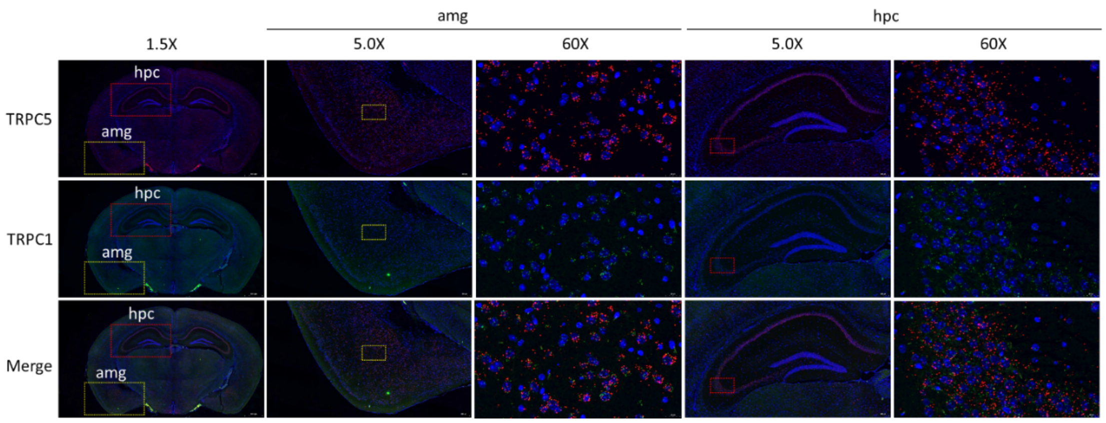

Supplementary Figure 10e Expression of TRPC5 and TRPC1 in mouse brain.

5. The manuscript does include any data on how the blockade of TRPC1/5 heteromers could affect the neuronal circuits controlling anxiety and/or depression. Does it lead to changes in neuronal excitability or synaptic transmission in specific pathways, or both?

Response: We appreciate this insightful question. To address it, we combined RNAscope expression mapping with ex vivo patch-clamp recordings.

Consistent with previous reports, RNAscope revealed substantial co-expression of TRPC1 and TRPC5 in the amygdala and hippocampus, regions implicated in anxiety and depression. We therefore focused on the lateral amygdala (LA).

Using CCK-4 to model anxiety-related neuronal activation (Zwanzger et al., *Depress Anxiety.*, 2012. PMID: 22553078; Riccio, *J Neurosci.*, 2014. PMID: 24599464), we observed that CCK-4 significantly increased action potential firing in LA neurons ($p < 0.001$). Importantly, application of JD03-02 in the presence of CCK-4 restored firing rates to control levels ($p > 0.05$), indicating that TRPC1/5 inhibition effectively counteracts pathological neuronal hyperexcitability.

While the present study primarily demonstrates an effect on neuronal excitability, we acknowledge that TRPC channels may also influence synaptic transmission. Previous work has shown that deletion of TRPC1/4/5 affects synaptic function (Broker-Lai et al., *EMBO J.*, 2017. PMID: 28790178). We therefore speculate that TRPC1/5 heteromers may also modulate synaptic transmission, a possibility that we explicitly acknowledge and propose to investigate in future studies.

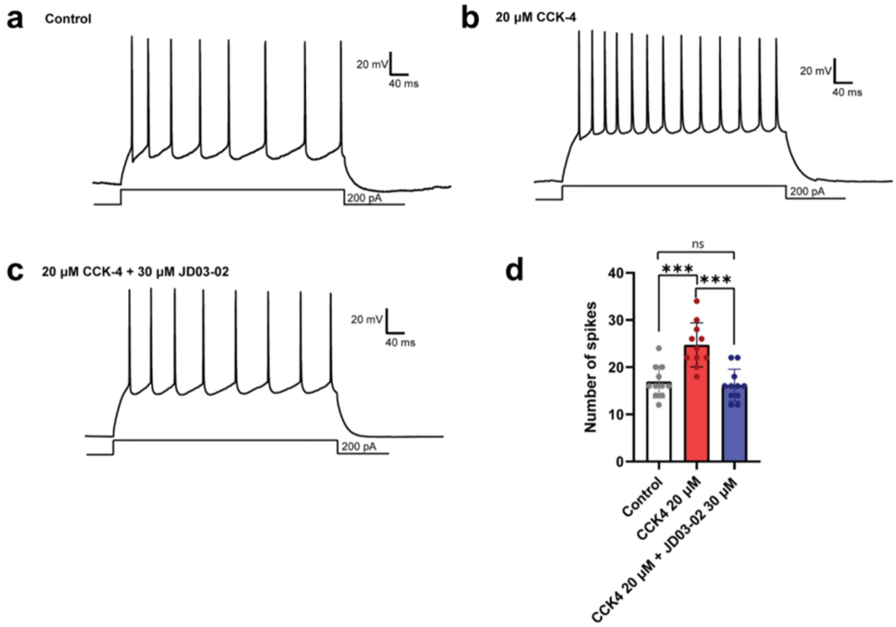

Supplementary Figure 10a-d

6.The systemic delivery of JD03-02 in behavioral experiments might be suboptimal, and region-specific infusions of the antagonist could strengthen the study.

Response: We fully agree with the reviewer that region-specific manipulation would be valuable. In an effort to address this, we conducted pilot stereotaxic intracranial infusion experiments. However, we found that the sham surgical procedures alone caused significant and persistent impairments in locomotion, introducing substantial confounds that precluded reliable interpretation of anxiety- and depression-related behaviors.

Given these limitations, we opted not to pursue this approach further at the current stage. Importantly, our ex vivo electrophysiological data already provide direct mechanistic evidence that JD03-02 acts on TRPC1/5 heteromers to modulate neuronal excitability within anxiety-related circuits. While in vivo region-specific delivery remains an important future direction, we believe the current dataset provides a robust and mechanistically grounded foundation.

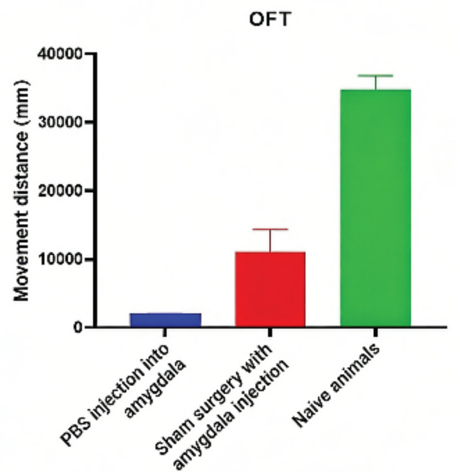
OFT
Movement distance (mm)
40000
30000
20000
10000
0
PBS injection into amygdala
Sham surgery with amygdala injection
Naive animals

REVIEWER COMMENTS

Reviewer #1 (Remarks to the Author):

We sincerely thank the reviewer for the careful evaluation of our revised manuscript and for the insightful and constructive comments, which have greatly helped us to further improve the quality and rigor of this study. Below, we provide point-by-point responses to the specific questions raised.

Q2: I am still not convinced that the shift in EC_{50} alone is sufficient to define the specific assembly of the TRPC1-C5 heteromer. Consider an alternative scenario: TRPC1 and C5 subunits assemble randomly, but TRPC1 homomers are non-functional. In this case, the observed TRPC1-C5 heteromeric population could exhibit a range of EA EC_{50} values, with the TRPC5 homomer (3.6 nM) at one extreme and a complex containing three TRPC1 subunits and one TRPC5 subunit (e.g., ~60 nM) at the other. Biasing the transfection ratio towards 3:1 or 6:1 (TRPC1:C5) would increase the proportion of complexes with a higher TRPC1 content, which aligns well with the results in Fig. 1b. For the current manuscript, the inclusion of the FRET results has greatly strengthened this section, and I have no further questions on this point.

Response: We fully agree with the reviewer that shifts in EC_{50} values alone are insufficient to define a specific heteromeric assembly. This consideration motivated us to incorporate FRET-based experiments in the previous revision, providing independent evidence for direct interactions between TRPC1 and TRPC5 subunits. We sincerely appreciate the reviewer's recognition that the inclusion of the FRET data has substantially strengthened this section. These suggestions have significantly enhanced the rigor and mechanistic clarity of our interpretation of the assembly process.

Q3: The decreased affinity of EA at the TRPC1-C5 interface, inferred from structural data, correlates with its reduced functional potency. However, does this imply that EA must bind to all four subunits to open the channel? Could the authors provide the Hill coefficients for EA activation of TRPC5 homomers and TRPC1-C5 heteromers? Furthermore, two other highly relevant studies should be cited:

https://www.biorxiv.org/content/10.1101/2025.07.09.663840v1

https://www.nature.com/articles/s41467-025-67024-9

Response: Our proposed model indeed suggests that, in the TRPC1–C5 heterotetramer, the markedly reduced affinity of EA at the TRPC1–C5 interface may require the occupation of all four inter-subunit binding sites (two TRPC1–C5 interfaces and two TRPC5–C5 interfaces) to reach an activation threshold comparable to that of the TRPC5 homotetramer. However, we also consider it likely that partial occupancy could still induce conformational changes sufficient to produce partial activation. We thank the reviewer for raising this critical mechanistic question, which substantially deepened our analysis of the channel gating process.

Based on our concentration–response curve fitting, the Hill coefficients for EA-mediated activation of both TRPC5 homomers and TRPC1–C5 heteromers are greater than 1 (see figure below), indicating pronounced positive cooperativity. This suggests that channel activation likely involves cooperative binding of multiple ligand molecules, a feature typical of ligand-gated ion channels.

Nevertheless, the specific question of whether all four subunits must be occupied by EA to achieve channel opening cannot be conclusively resolved based on our current structural data or the existing literature. Two recent highly relevant structural studies provide important context:

1. Kim et al. (Nat Commun, 2025) resolved the structure of the TRPC5 homomer bound to EA and found that, despite full occupancy of all four subunits, the channel remained in a closed conformation. This observation indicates that complete ligand binding alone is insufficient to guarantee channel opening, pointing to a more complex coupling mechanism between ligand binding and gating.
2. Porav et al. (bioRxiv, 2025) captured multiple intermediate conformations of TRPC5 bound to EA, including states in which only one to three subunits are occupied. These authors proposed that these structures may represent key intermediates along the activation pathway, supporting a model of progressive and cooperative gating.

Taken together, these studies suggest that EA binding and channel activation involve a highly dynamic and cooperative conformational landscape. Partial subunit occupancy may be sufficient to trigger early conformational transitions, whereas full occupancy may correspond to later or more stabilized states along the activation pathway, but is not necessarily the sole determinant of channel opening. Our functional data, showing Hill coefficients greater than 1, are fully consistent with such a cooperative gating model.

We have now cited these two important studies in the revised manuscript (Page 6, lines 149–152).

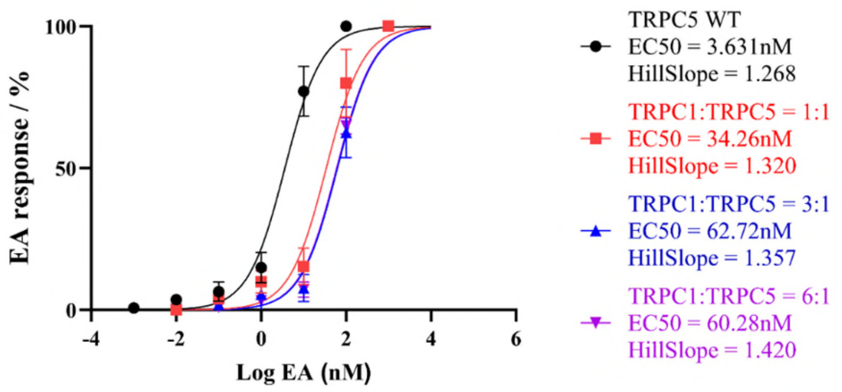

Q4: The authors propose that JD03-02 (H08) binds with high affinity at the TRPC1-C5 interface but with low affinity at the TRPC5-C5 interface. If this model and negative cooperativity hold, the Hill coefficient for JD03-02 on the TRPC1/C5 heteromer should be close to 1, while its coefficient on the TRPC5 homomer should be similar to those of other inhibitors. The authors should re-examine and potentially repeat these experiments to verify this point.

Response: We thank the reviewer for this insightful and important suggestion. The predicted Hill coefficients derived from our binding model and the assumption of negative cooperativity indeed provide a critical benchmark for evaluating the validity of this mechanism. We fully agree with this reasoning and therefore performed additional experiments and analyses to directly address this point.

Following the reviewer's suggestion, we reanalyzed the concentration–response relationship of JD03-02 on the TRPC1/C5 heteromer and supplemented the dataset with additional measurements at lower concentrations (0.01 nM and 0.001 nM). The combined fitting yielded a Hill coefficient of approximately 0.33, which remains markedly below 1 and is consistent with our previously obtained value of ~0.49 (See the figure below).

Although this value is lower than the ~1 predicted by a simplified two-site negative cooperativity model, we interpret this result as indicative of particularly strong negative cooperativity. Given the exceptionally high potency of JD03-02 toward the TRPC1/C5 heteromer (IC_{50} < 0.1 nM), binding of the first molecule to the high-affinity TRPC1–C5 interface may induce pronounced conformational coupling that substantially diminishes the apparent affinity of the second equivalent site, rendering it functionally inaccessible within the experimentally observable concentration range. Consequently, the concentration–response curve rapidly reaches a plateau at very low concentrations, resulting in an apparently reduced Hill coefficient. Such behavior is consistent with an extreme form of negative cooperativity, in which the first binding event strongly suppresses subsequent ligand binding.

Notably, a similar trend was observed for the non-selective inhibitor HC-070, which also exhibited a Hill coefficient <1 for inhibition of the TRPC1/C5 heteromer, further supporting the presence of negative cooperativity at the heteromeric interface.

As a critical control, we further reexamined the effect of JD03-02 on the TRPC5 homotetramer using both patch-clamp recordings and calcium flux assays. In contrast to its high potency toward the heteromer, JD03-02 displayed only very weak inhibition of the TRPC5 homomer, with detectable effects emerging only at 5 μM in patch-clamp recordings or 25 μM in calcium flux assays (See the figure below), producing ~20% inhibition. Due to this extremely low efficacy, reliable concentration–response fitting and Hill coefficient determination were not feasible. These results further demonstrate the strong selectivity of JD03-02 for the TRPC1/5 heteromeric interface and its minimal activity toward the TRPC5 homomer.

Together, these findings reinforce our model that JD03-02 preferentially targets the TRPC1/5 interface and induces strong negative allosteric coupling within the heteromeric complex. We have incorporated mechanistic interpretations into the revised manuscript and expanded the discussion accordingly (Page 15, lines 426–432). "Pharmacological analysis further supports a distinct mechanism of action. Inhibition of the TRPC1/5 heteromer by JD03-02 is characterized by a low Hill coefficient (~0.49), consistent with negative cooperativity, similar to that observed for the non-selective inhibitor HC-070, whereas its activity toward TRPC5 homomers is negligible. Together, these features are compatible with a model in which JD03-02 preferentially engages a binding pocket formed at the heteromeric interface, linking structural insights to selective pharmacological modulation."

New data

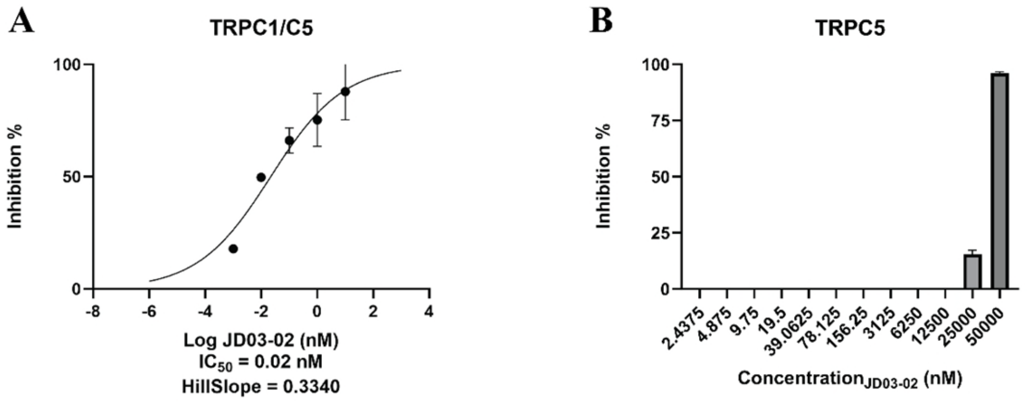

Original data

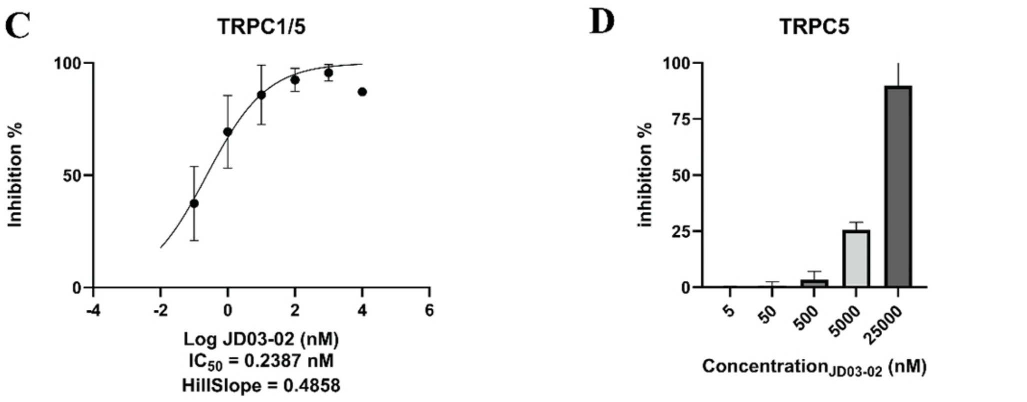

Fig. Selective inhibition of TRPC1/5 heteromers by JD03-02 revealed by electrophysiological and calcium imaging assays

A. Concentration–response relationship of JD03-02–mediated inhibition of the TRPC1/5 heteromer, measured by whole-cell patch-clamp recordings. Data are presented as mean ± SEM (n = 5).

B. Inhibitory effect of JD03-02 on the TRPC5 homomer, measured by calcium flux assays. Data are presented as mean ± SEM (n = 3).

C. Concentration–response relationship of JD03-02–mediated inhibition of the TRPC1/5 heteromer, measured by whole-cell patch-clamp recordings. Data are presented as mean ± SEM (n = 5).

D. Inhibitory effect of JD03-02 on the TRPC5 homomer, measured by whole-cell patch-clamp recordings. Data are presented as mean ± SEM (n = 4).
